# A systematic review and meta-analysis of the evidence for community-based HIV testing on men’s engagement in the HIV care cascade

**DOI:** 10.1177/09564624221111277

**Published:** 2022-07-03

**Authors:** Allison K Groves, Petra Stankard, Sarah L Bowler, Muhammad S Jamil, Luwam T Gebrekristos, Patrick D Smith, Caitlin Quinn, Ndoungou Salla Ba, Thato Chidarikire, Van Thi Thuy Nguyen, Rachel Baggaley, Cheryl Johnson

**Affiliations:** 1 Dornsife School of Public Health, 37412 Drexel University, Philadelphia, PA, USA; 2 Independent Consultant, Washington, DC, USA; 3 Department of HIV/AIDS, 3489 World Health Organization, Geneva, Switzerland; 4 HIV Prevention Programmes, National Department of Health, Johannesburg, South Africa

**Keywords:** Community, testing, HIV care continuum, HIV prevention, gender, men who have sex with men

## Abstract

**Objective:**

Men with HIV are less likely than women to know their status, be on antiretroviral therapy, and be virally suppressed. This review examined men’s community-based HIV testing services (CB-HTS) outcomes.

**Design:**

Systematic review and meta-analysis.

**Methods:**

We searched seven databases and conference abstracts through July 2018. We estimated pooled proportions and/or risk ratios (for meta-analyses) for each outcome using random effects models.

**Results:**

188 studies met inclusion criteria. Common testing models included targeted outreach (e.g. mobile testing), home-based testing, and testing at stand-alone community sites. Across 25 studies reporting uptake, 81% (CI: 75–86%) of men offered testing accepted it. Uptake was higher among men reached through CB-HTS than facility-based HTS (RR = 1.39; CI: 1.13–1.71). Over 69% (CI: 64–71%) of those tested through CB-HTS were men, across 184 studies. Across studies reporting new HIV-positivity among men (*n* = 18), 96% were newly diagnosed (CI: 77–100%). Across studies reporting linkage to HIV care (*n* = 8), 70% (CI: 36–103%) of men were linked to care. Across 57 studies reporting sex-disaggregated data for CB-HTS conducted among key populations, men’s uptake was high (80%; CI: 70–88%) and nearly all were newly diagnosed and linked to care (95%; CI: 94–100%; and 94%; CI: 88–100%, respectively).

**Conclusion:**

CB-HTS is an important strategy for reaching undiagnosed men with HIV from the general population and key population groups, particularly using targeted outreach models. When compared to facility-based HIV testing services, men tested through CB-HTS are more likely to uptake testing, and nearly all men who tested positive through CB-HTS were newly diagnosed. Linkage to care may be a challenge following CB-HTS, and greater efforts and research are needed to effectively implement testing strategies that facilitate rapid ART initiation and linkage to prevention services.

## Introduction

Globally, an estimated 37.7 million people are living with HIV, and approximately 680,000 AIDS-related deaths occurred in 2021.^1^ The Joint United Nations Programme on HIV/AIDS (UNAIDS) has introduced bold fast-track targets to reduce HIV incidence by ensuring that 95% of all people with HIV know their status, that 95% of people with a known HIV-positive status are receiving antiretroviral treatment (ART), and that 95% of people on ART achieve viral suppression.^2^ While these targets have galvanized scale-up of HIV testing and treatment, gaps remain, particularly among men. As of 2019, only 82% of HIV-positive men globally knew their status (vs 88% of women), 68% of HIV-positive men were on treatment (vs 79% of women), and 62% of HIV-positive men had achieved viral suppression (vs 72% of women).^1^ While gendered disparities in 95–95–95 outcomes persist across all regions, they are greatest in sub-Saharan Africa.^1^

Men face multiple barriers to facility-based HIV testing services (FB-HTS). In settings of generalized epidemics, barriers include individual factors (knowledge, fear of HIV positivity or disclosure),^3–5^ social factors (HIV-related stigma),^4,6^ and factors related to testing environments (long waits; perceptions that facility nurses are rude or unfriendly toward men seeking testing; and confidentiality concerns).^4,5,7–10^ Further, whereas FB-HTS is routinely available for women through existing clinical services (e.g. antenatal care),^11,12^ efforts to integrate HIV testing into clinical services sought by men are underexamined, despite some preliminary evidence that such models may contribute to higher HIV test uptake than standalone HIV testing services.^13–18^ Moreover, masculine norms which value strength, self-reliance and maintenance of traditional social roles may decrease access to FB-HTS.^4,7–10,19,20^

For men in key populations (e.g. men who have sex with men (MSM), male sex workers, and men who inject drugs), these barriers may be compounded by stigma and discrimination that restrict the availability, accessibility, and uptake of HIV testing and other health services.^21–25^ Social and structural factors (such as laws criminalizing same-sex relations) may restrict HIV testing accessibility for key populations,^26^ while also impacting the knowledge, attitudes, and practices of healthcare providers responsible for assessing HIV risk, offering testing, communicating results, and initiating treatment.^21,27,28^ Consequently, members of key populations may have heightened concerns regarding the confidentiality of their test results and sexual identity,^29,30^ particularly in settings with punitive laws related to HIV test results and/or sexual identity.^31^

The World Health Organization has recommended community-based HIV testing (CB-HTS) models to address such barriers and facilitate early HIV detection.^32^ The high uptake (>85%) among men who were offered home-based testing in six African countries suggests that, when offered community-based testing, men accept it^33^; a recent scoping review across sub-Saharan Africa reported similar findings.^34^ In two prior systematic reviews of testing in sub-Saharan Africa before 2015 (one which explicitly examined CB-HTS, the other which examined several HIV testing strategies, including CB-HTS, on men’s uptake), outreach testing models were the most likely community-based model to test men.^35,36^ A global systematic review of literature up to early 2013 also reported that workplace models tested high proportions of men (67% of those tested were men). In prior reviews, the majority of individuals tested within other CB-HTS models, like home-based testing, were women.^35–37^

While prior reviews report demographics of individuals accessing CB-HTS, no reviews describe global outcomes along the full HIV care cascade for all men (from both general and key populations), examine which CB-HTS models test high proportions of men, or examine whether outcomes differ for men in key population groups and general populations. Therefore, this global systematic review aims to describe how CB-HTS strategies impact men’s testing uptake and engagement within the HIV care cascade.

## Methods

We conducted a systematic review in accordance with PRISMA guidelines.^38^ Observational and experimental studies were included if they were published before 1 July 2018, included CB-HTS interventions (detailed definition in Table 1), provided data disaggregated by sex, and reported on any of the following outcomes: (1) HIV testing uptake, (2) proportion of those tested who are male, (3) proportion newly diagnosed with HIV, (4) proportion linked to care, (5) proportion who initiate ART, (6) report of retention in care or (7) report of viral suppression among those on treatment (detailed definitions in Supplemental Digital Content 1). No exclusion was placed on geographic region, language or population engaged (i.e. we did not restrict our search to any particular population or demographic characteristics). Surveillance studies and surveys were excluded as they did not report on CB-HTS as an intervention. HIV self-testing studies were excluded from the review because these are defined as separate from CB-HTS by the WHO.

**Table 1.** Testing approach definitions.

Testing approach	Description
Home-based testing	Testing offered at the household level, often in door-to-door campaigns
Index/partner notification	Community-level testing of the sexual partners, household members and children of recently identified HIV-positive individuals
Stand-alone community sites	Testing in fixed settings at community-level where HIV testing is exclusively offered. This may include voluntary counseling and testing centers, as well as the offices of community-based organizations
Outreach	Testing is done in non-clinical settings often in high-prevalence areas where populations are likely to congregate. This includes commercial settings (e.g. sex on premises venues), social service venues (e.g. needle exchange programmes), large events (e.g. gay pride parades), other venues (e.g. pharmacies) and street-based testing in mobile vans
Index testing for TB	This testing approach identifies individuals with tuberculosis at clinics or hospitals and offers home-based HIV testing to their household contacts
Workplace	Testing is offered in the workplace
School	Testing is offered in the school
Combination	Community-based testing may also use a hybrid of these approaches using one or more of the above models. For example, door-to-door home-based testing complemented with outreach campaigns

### Information sources and search strategy

Seven databases (see table, Supplemental Digital Content 2) were searched at two time points (1) February–March 2015 (for studies published before 31 December 2014); and (2) August–November 2018 (for studies published between 1 January 2015 and 1 July 2018). All studies published before 1 July 2018 were screened. Given our comprehensive search across seven databases, we did not manually search the reference lists of publications. However, to account for publication lags, we searched electronic conference abstracts from three HIV conferences between 2015–2018. All records were collated in Zotero and duplicates were removed prior to screening.

Search terms were adapted from a 2013 review by Suthar et al.^37^ (see table, Supplemental Digital Content 2). To search HIV-related conference abstracts, only terms “HIV” and “test” were used as keywords.

### Data screening and extraction

All abstracts were independently screened by two authors (AG and PS) for inclusion. Disagreements were resolved through discussion. Data were independently extracted by PS, AG, SB, SS, LN and KO. Extractors followed a strict protocol and held weekly meetings to ensure robust extraction.

**Figure 1.**
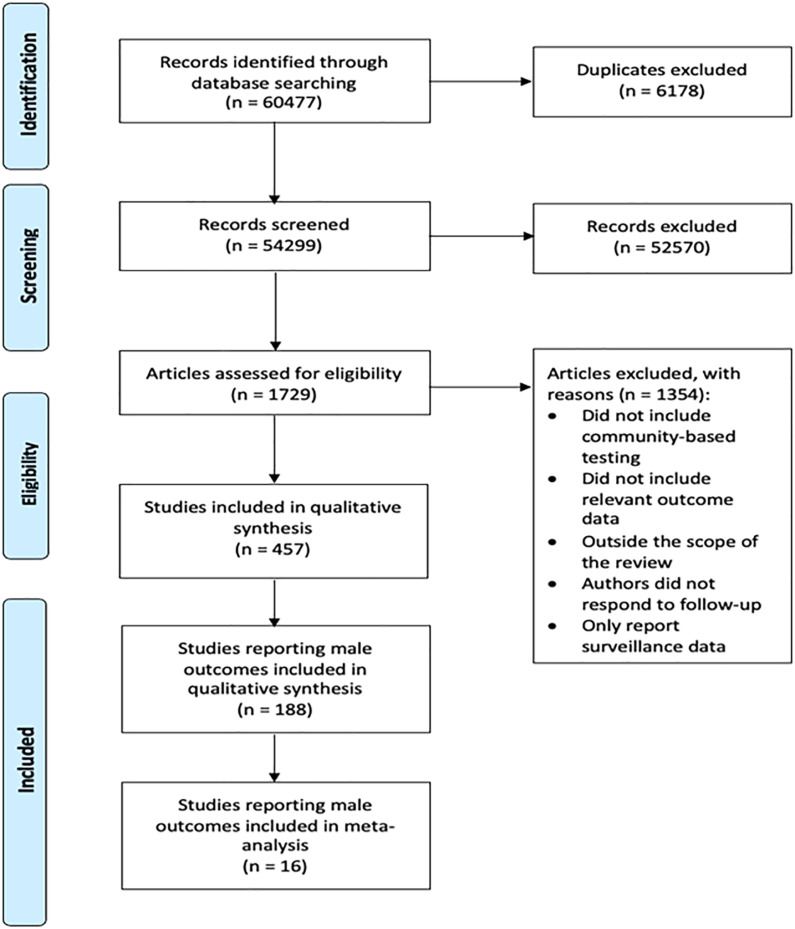
Consort diagram.

In total, 60,477 unique records were screened; full text from 1729 studies was assessed for eligibility (Figure 1). Ultimately, 457 studies met inclusion criteria. Full text was independently assessed for eligibility by two reviewers, which yielded 188 studies for inclusion (see table, Supplemental Digital Content 3).^39–195^ Most studies were cross-sectional (*n* = 115, 61.17%), followed by cohort (*n* = 31, 16.49%), quasi-experimental (*n* = 28, 14.89%) and randomized trials (*n* = 14, 7.45%). Two-thirds of included studies were published after 2013, and half of included studies were published after 2015 (see figure, Supplemental Digital Content 4).

Study quality was assessed using two tools (see tables, Supplemental Digital Content 5a and 5b). For randomized studies reporting male outcomes with a comparator arm, we used the Cochrane Collaboration’s ‘‘risk of bias’’ tool,^196^ which assesses potential for bias arising from randomization, missingness, outcome measurement, and selective reporting. This scale ranges from 0–6; higher scores indicate lower risk of bias. Eight experimental studies had sufficient data for evaluation; studies received a mean score of 3.5, indicating moderate risk of bias.

For non-randomized quasi-experimental studies reporting male outcomes, we used the Newcastle-Ottawa Quality Assessment Scale.^197^ This scale ranges from 0–8 and assesses study quality based on selection bias, potential for confounding, and measurement bias. Higher scores indicate lower risk of bias. 18 quasi-experimental studies had sufficient data for Newcastle-Ottawa Scale evaluation; studies received a mean score of 4.0, indicating average study quality.

### Statistical analysis

Random effects meta-analysis of single proportions was used to summarize results for each main outcome across all studies reporting the outcome. Proportions were stabilized using Freeman-Tukey transformation.^198^ We conducted a meta-analysis of studies that compared outcomes for participants who received CB-HTS to outcomes for participants who received FB-HTS, using random effects models with the metan package in Stata v18. We report pooled relative risks (RRs) and present forest plots where appropriate. The *I*^*2*^ statistic for the meta-analytic output was used to measure heterogeneity with values of 25%, 50% and 75% indicating low, moderate, high heterogeneity, respectively.^199^ Analyses were conducted with Stata v17 and SAS software.^200,201^

## Results

Of 188 studies that evaluated at least one CB-HTS outcome for men, over two-thirds focused on men from the general population (GP) (*n* = 131, 69.68%). Of the 57 remaining studies focusing on men from key populations (KP), over half were on MSM (*n* = 32, 56.14%). Other studies that reported on male outcomes within KP focused on people who inject drugs (PWID) (*n* = 7, 12.28%) or mixed populations (*n* = 18, 31.57%). (See graphs, Supplemental Digital Content 6a–6e and 7a–7e, for forest plots of outcomes by population).

The most common CB-HTS models identified in this review were outreach (*n* = 92, 48.94%), home-based (door-to-door) (*n* = 50, 26.60%), stand-alone community sites (*n* = 19, 10.11%), combination models (*n* = 14, 7.44%), and school-based models (*n* = 6, 3.19%). All other models (i.e. index testing (*n* = 3), TB index testing (*n* = 1), workplace models (*n* = 3)) had few studies reporting on male outcomes. Nearly 85% of GP studies used home-based (*n* = 50, 38.17%) or outreach models (*n* = 61, 46.56%), and over 80% of KP studies used outreach models (*n* = 31, 54.39%) or stand-alone community sites (*n* = 14, 24.56%) (see graphs, Supplemental Digital Content 8a–8j, for forest plots of outcomes by model).

Half of all included studies occurred in sub-Saharan Africa (*n* = 104, 55.32%) (see map, Supplemental Digital Content 9). The remainder occurred in the Americas (*n* = 43, 22.87%), Europe (*n* = 24, 12.77%), Western Pacific (*n* = 9, 4.79%) or Southeast Asia (*n* = 8, 4.26%). Most GP studies took place in Africa (*n* = 97, 74.05%) or the Americas (*n* = 20, 15.27%). KP studies were more geographically diverse: 42% occurred in the Americas (*n* = 24), 28% in Europe (*n* = 16), 12% in the Western Pacific Region (*n* = 7), 11% in Africa (*n* = 6) and 7% in Southeast Asia (*n* = 4). (see graphs, Supplemental Digital Content 10a–10i, for outcomes by region).

### Uptake of HIV testing

**Figure 2.**
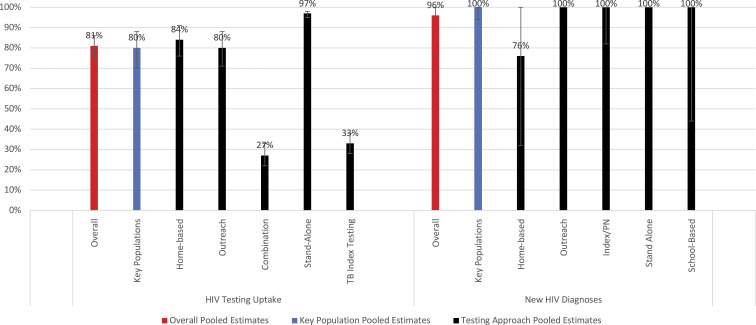
Uptake and new HIV diagnoses of men reached through CB-HTS.

**Figure 3.**
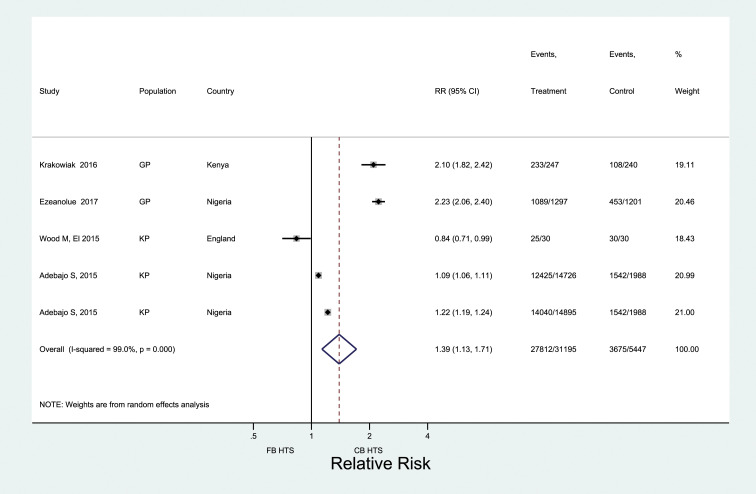
Comparing uptake of HIV testing for men reached through CB HTS and facility-based HTS.

Across 25 studies, 19 of which came from Africa, 400,632 men were offered testing, and 306,945 men received HIV testing (Figure 2). Pooled male testing uptake was 81% (95% CI: 75–86%); nearly half of all men (48%) were first-time testers. When restricted to five rigorous (i.e. experimental/quasi-experimental) studies, uptake was significantly higher among men offered CB-HTS than FB-HTS (RR: 1.39; 95% CI: 1.13–1.71) (Figure 3). High heterogeneity was observed across the five estimates (*I*^*2*^ = 99.0%).

Home-based (*n* = 14) and outreach testing (*n* = 7) models had high male uptake, with pooled testing uptake of 84% (95% CI: 76–91%) and 80% (95% CI: 71–88%), respectively. The sample size for specific outreach approaches (e.g. sex-on-premises venues, pharmacies, religious venues) is too small to report meaningful variation in uptake by outreach approach. Uptake was high among studies in Africa (*n* = 19): 82% (95% CI: 76–88%); sample sizes for other regions are too small to report meaningful regional variation in uptake.

### Proportion of males among those tested

**Figure 4.**
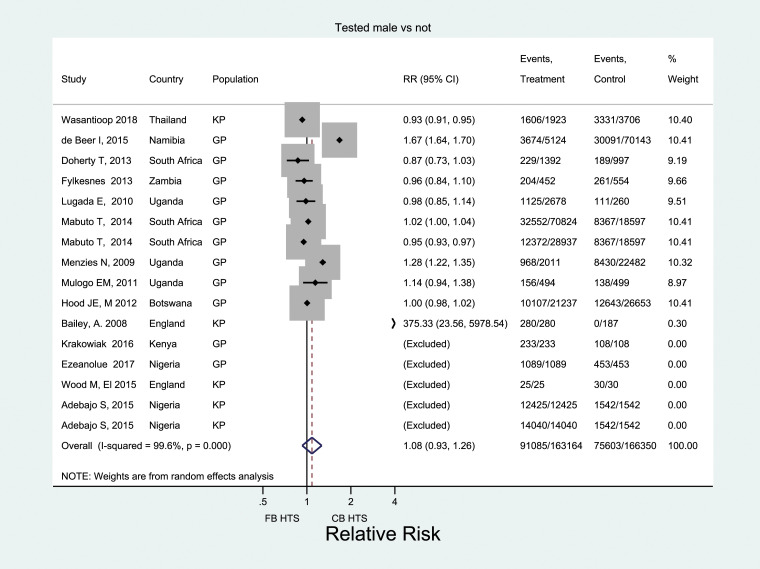
Comparing proportion of males among those tested through CB HTS and facility-based HTS.

Across 184 reporting studies that offered CB-HTS, over two-thirds (69%) of those tested were men (95% CI: 64–71%). Just over half (58%) of those tested in Africa were men (95% CI: 54–61%), whereas in Europe and the Western Pacific, an overwhelming majority of those tested were men: 87% (95% CI: 78–94%) and 93% (95% CI: 63–100%), respectively. When restricted to 11 rigorous studies, the percentage of male testers was 74% (95% CI: 58–87%) in CB-HTS and 71% (95% CI: 63–79%) in FB-HTS. This difference was not statistically significant (RR: 1.08; 95% CI: 93–1.26) and heterogeneity was high (*I*^*2*^ = 99.6%) (Figure 4).

Stand-alone community sites (*n* = 20) tested the highest percentage of males (89%) (95% CI: 75–98%). Combination (*n* = 13), workplace (*n* = 3), and outreach (*n* = 88) models also tested primarily men, at 91% (95% CI: 65–100%), 78% (95% CI: 51–95%), and 72% (95% CI: 67–76%), respectively.

### Proportion newly diagnosed with HIV

Across 18 studies, 6717 men who tested positive were newly diagnosed. The pooled prevalence of new HIV diagnosis was 96% (95% CI: 77–100%). Most studies reporting this outcome occurred in Africa (*n* = 11), where 90% of individuals were newly diagnosed (95% CI: 62–100%) or Europe (*n* = 5), where all individuals were newly diagnosed (95% CI: 100–100%). Stand-alone community sites (*n* = 2) and outreach testing models (*n* = 8) yielded the highest numbers of newly diagnosed men (100% new diagnoses) (Figure 2). Home-based (*n* = 6) testing models were more likely to include repeat testers than other models.

### Proportion linked to care

In total, 736 HIV-positive men were linked to care, across six studies spanning four continents. Pooled estimates indicate that 70% of HIV-positive men were linked to care (95% CI: 36–103%) (Table 2). Outreach and combination approaches had the highest linkage rates. One home-based testing study from 2013 reported the lowest linkage to care (22%).^202^ This study, along with three others demonstrating higher linkage (75%,^130^ 80%,^54^ 97%^114^), were conducted before 2016 recommendations regarding immediate ART initiation.^14^

**Table 2.** Males linkage and ART initiation.

Author	Pub year	Country	Key population	Testing approach	Testing setting	Linkage to care	Linkage definition	ART initiation
Floyd	2017	Zambia	n/a	Home-based	Home-based	n/a	n/a	64%
Medley	2013	Kenya	n/a	Home-based	Home-based	22%	Enrolled or registered in care	5%
Williams	2018	Eswatini	n/a	Outreach	Mobile vans	96%	Not given	95%
Qvist	2014	Denmark	MSM	Combination	Stand alone and mobile vans at high prevalence locations	97%	Enrolled or registered in care	97%
Daskalakis	2009	USA	MSM	Outreach	Saunas/bath houses	75%	Enrolled or registered in care	n/a
Khawcharoenporn	2017	Thailand	MSM	Outreach	Saunas/bath houses	48%	Enrolled or registered in care	n/a
Champenois	2012	France	MSM	Stand-alone community site	Community-based organization	80%	Enrolled or registered in care	n/a

### Proportion who initiated ART

The pooled estimate of ART initiation across four studies was 67% (95% CI: 25–98%) (Supplemental Table 4). Among the two home-based testing studies in Africa that reported ART initiation, one 2013 study reported that few (5%) HIV-positive men initiated ART,^196^ whereas the other, published in 2017, reported that nearly two-thirds initiated ART.^203^ In contrast, a 2018 study in Africa that used outreach testing plus peer-based case management services reported 94% ART initiation.^204^ Finally, another study in Europe, published in 2014, reported very high ART initiation (97%) among MSM who were offered testing through multiple venues (i.e. walk-in clinic, mobile testing at saunas, sex parties, and Pride parades).^114^ The two studies reporting the highest and lowest ART initiation were published before WHO’s recommendation of test and treat.

### Proportion retained in care and/or reporting viral suppression

No studies reported sex-disaggregated retention in care. Only one study in Europe reported men’s viral suppression.^114^ Among MSM who were offered combination testing, all but one achieved full viral suppression (median 8 months, IQR 5–19 months).^114^

### Outcomes for men within key populations

Sex-disaggregated data for CB-HTS conducted among key populations was reported in 57 studies, 32 of which focused on MSM. Male uptake of CB-HTS within key populations, reported in eight studies across four regions, was 80% (95% CI: 70–88%). Across all regions, males comprised 91% (95% CI: 82–97%) of those tested through CB-HTS models targeting key populations. All men diagnosed with HIV through key population programming were newly diagnosed (95% CI: 94–100%). Linkage to care for HIV-positive men receiving CB-HTS through key population programming was 94% (95% CI: 88–100%), higher than men overall.

## Discussion

We conducted the first global systematic review of studies implementing CB-HTS among men (general population and key populations) and found that – compared to FB-HTS – CB-HTS is highly effective in increasing men’s uptake of testing, particularly for men with previously undiagnosed HIV. In the included studies, over two-thirds of those tested through CB-HTS were men, which is higher than reported in a previous review focused only in sub-Saharan Africa (in which over half of those tested were women).^35^ Our estimate is likely high because it includes approaches which explicitly targeted MSM and also includes recent efforts to address testing gaps among men with differentiated testing models.^205^ Still, populations reached by CB-HTS comprise greater percentages of men than previously estimated. Nevertheless, there are persistent gaps in understanding how CB-HTS affects men’s care engagement following HIV diagnosis, particularly outside of Africa. While existing evidence on linkage to care, ART initiation, and viral suppression among men receiving CB-HTS was limited, our review showed high linkage among men newly diagnosed (mostly MSM) from key population programmes. Such findings are substantial as men from key population groups face unique stigmas and barriers to HIV testing and treatment. Further examination of linkage to care, ART initiation, and viral suppression among *all men* who receive CB-HTS is urgently needed to understand how different testing approaches may help achieve global HIV targets.

Effectively delivering CB-HTS to men requires differentiated delivery employing multiple testing models. Our review examined several such models. Targeted outreach was most effective in increasing HIV testing uptake and new diagnoses, building on PEPFAR data demonstrating high yield for mobile testing in several countries.^206^ Available evidence (albeit of limited quality) also shows high levels of linkage in outreach models. Further work is needed to understand which linkage models convert improvements in male testing uptake to increased treatment coverage.

Findings highlight that targeted outreach is essential for reaching men with HIV from general and key populations. Targeted outreach strategies are diverse, and our study found outreach HIV testing offered in commercial settings (e.g. bars, sex-on-premises venues), social service venues (e.g. needle exchange programmes), large events (e.g. pride parades, health fairs), faith-based organizations, pharmacies and street-based mobile van testing sites. This diversity of settings enables targeted outreach approaches to effectively deliver differentiated testing, as recommended by WHO.^205^ To strengthen differentiated testing models, future research might examine cascade outcomes by setting type. Research is also needed to identify key components of outreach delivery associated with impact across settings.

Our review found that stand-alone community sites (like drop-in centers) are effective in reaching undiagnosed men with HIV, particularly MSM, outside of health facilities. Such stand-alone sites are often more accessible than health facilities, and may be more trusted by community members.^4,7,10,207^ Such sites are often situated within community-based organizations which offer HTS alongside many other services. Recently, stand-alone testing has declined given its resource-intensive nature. Our findings suggest that in certain settings, maintaining such services is important to reaching men, particularly those in key populations.

In contrast, the home-based testing studies we reviewed demonstrated mixed outcomes in engaging men. While uptake was high, likely due to reduced logistical barriers, new diagnoses and linkage to care were the lowest across all models. Our findings suggest that this resource-intensive approach should be carefully employed. Home-based testing may hold particular promise for re-engaging men in care, because they disengage from care at higher rates than women.^208,209^ Other efforts to modify home-based testing delivery could be explored, including offering testing in the evenings/weekend or offering HIV self-test kits for men who are not home during testing.^210^ Our findings also suggest the need for robust linkage support in home-based testing. However, linkage findings may be skewed by the small sample reporting on male-specific linkage, particularly because half of the included studies were published prior to 2014.

Overall, while our review found encouraging evidence that over two-thirds of men tested through CB-HTS and diagnosed with HIV were linked to care, the small number of studies and lack of rigorous study design on linkage to care, ART initiation, care retention, and viral suppression limits assessment of whether improvements in CB-HTS HIV testing uptake can directly translate to epidemiological impact globally. Existing data posed multiple challenges to understanding outcomes of CB-HTS later in the cascade. First, studies used differing definitions of linkage to care, hampering comparability. This is particularly problematic for studies published before the “test and treat” era, as criteria for enrollment in care or pre-ART varied widely. An additional challenge is that few CB-HTS studies detailed how they supported men’s linkage to care, which hampers evaluation of different linkage strategies. Viral suppression and retention in care were rarely reported. There is urgent need to better evaluate linkage packages for CB-HTS and to track viral suppression and retention in care longitudinally, particularly among men.

Finally, based on the studies reviewed, CB-HTS appears particularly effective for men in key populations. Across all outcomes, men reached through CB-HTS models which targeted key populations fared as well or better than men reached through CB-HTS models which targeted general populations. Testing key populations through outreach and other community-based models has been standard practice given their well-documented barriers to accessing health services.^211^ Nonetheless, knowledge gaps persist for certain vulnerable men within key populations. For example, while PWID^212^ and some male sex workers (MSW)^213,214^ face particularly high HIV risk, only two studies reported male outcomes (other than proportion men tested) for male PWID, and zero studies reported outcomes specifically for MSW. Further, across all studies, evidence of CB-HTS’s effect on linkage to care and viral suppression among key populations is nascent.

While growing evidence indicates that CB-HTS increases HIV testing coverage,^35^ studies do not consistently report sex-disaggregated outcomes. This lack of disaggregated data hampers understanding of the impacts of CB-HTS on testing coverage among men, precluding identification of effective strategies for engaging all men.

### Limitations

This review has several limitations. First, there was significant heterogeneity across studies. Pooled estimates should be interpreted with caution and with consideration of underlying social, cultural and epidemic variations. Relatedly, we were unable to fully assess regional variation given limited sample size for each outcome within each region. Second, while we report on the proportion of men tested in CB-HTS models, this outcome does not describe testing coverage within each testing catchment area. A lack of sex-disaggregated data on coverage (and on testing coverage overall) limits understanding of the impact of CB-HTS among men. Third, definitions of linkage to care varied across studies and studies published before 2013 and 2015 often had different treatment guidelines, which may have affected outcomes. Moving forward, standardized measures are key to assessing outcomes along the cascade. Relatedly, measures should also indicate whether linked individuals are newly diagnosed or re-engaged in care, which can help understand progress toward the second 95 target. Fourth, we did not examine outcomes reported for transgender women (TGW), even though they might have been considered for inclusion based on their assigned sex at birth. Understanding TGW’s testing uptake and care engagement is crucial given their risk of acquiring HIV and experiencing poor HIV-related outcomes.^215–217^ Fifth, we did not examine men’s linkage to prevention services following CB-HTS, which is essential for meeting global HIV goals. Sixth, we only conducted quality assessment for experimental and quasi-experimental studies (<20% of included studies) and did not assess the potential for bias across all studies. Finally, this review includes only published studies, which may limit generalizability.

## Conclusions

CB-HTS is an important strategy for reaching undiagnosed men with HIV from the general population and key population groups, particularly using targeted outreach models. When compared to FB-HTS, men tested through CB-HTS are more likely to uptake testing, and nearly all men who tested positive through CB-HTS were newly diagnosed. Linkage to care may be a challenge following CB-HTS, and greater efforts and research are needed to effectively implement testing strategies that facilitate rapid ART initiation and linkage to prevention services.

## Supplemental Material

Supplemental Material - A systematic review and meta-analysis of the evidence for community-based HIV testing on men’s engagement in the HIV care cascade Supplemental Material for A systematic review and meta-analysis of the evidence for community-based HIV testing on men’s engagement in the HIV care cascade by Allison K Groves, Petra Stankard, Sarah L Bowler, Muhammad S Jamil, Luwam T Gebrekristos, Patrick D Smith, Caitlin Quinn, Ndoungou Salla Ba, Thato Chidarikire, Van Thi Thuy Nguyen, Rachel Baggaley and Cheryl Johnson in International Journal of STD & AIDS
