# Supplementary material for: A systematic review and meta-analysis of the evidence for community-based HIV testing on men’s engagement in the HIV care cascade
Source: Int J STD AIDS. 2022 Jul 3;33(13):1090–105. doi: 10.1177/09564624221111277 (PMC9660288; doi:10.1177/09564624221111277)

**Supplemental Digital Content 1. Outcome Definitions**	
Outcome	*Definition*
Proportion of males tested	The number of males tested divided by total number of individuals tested
HIV testing uptake	The number of individuals tested divided by number of individuals offered testing or number of individuals eligible for testing. Both offered testing and eligible for testing were used to construct the denominator because of inconsistences in how studies reported uptake
Positivity	Assessed by the number of males testing HIV positive divided by total number of males tested
New HIV diagnoses	The number of males tested HIV positive for the first time divided by number of males testing HIV positive
First time testers	The number of individuals tested for the first time/total individuals tested
Linkage	The number of HIV-positive individuals linked to care divided by the total number of HIV-positive individuals
ART initiation	Created by dividing the number of HIV positive individuals who initiation/number of HIV positive individuals eligible to initiate
Viral suppression	Described qualitatively as there were very few studies that reported on suppression

Under review

Supplemental Digital Content 2. Search Strategy	
Databases Searched	
PubMED	
Embase	
WHO Global Index Medicus	
Cochrane Library	
International Clinical Trials Registry Platform	
International Standard Randomized Controlled Trial Number Register	
ClinicalTrials.gov	
Conference Abstracts Searched	
Conference on Retroviruses and Opportunistic Infections	
International AIDS Society Conference on HIV Sciences	
International AIDS Conference	
Search Number	*Search Terms*
1	HIV
2	human immunodeficiency virus
3	1 or 2
4	counsel*
5	test
6	testing
7	tested
8	5 or 6 or 7
9	community
10	home
11	house
12	door
13	mobile
14	campaign
15	bar
16	workplace
17	business
18	church
19	temple
20	active
21	school
22	highway
23	brothel
24	bathhouse
25	festival
26	outreach
27	van
28	bicycle
29	9 or 10 or 11 or 12 or 13 or 14 or 15 or 16 or 17 or 18 or 19 or 20 or 21 or
30	3 and 4 and 8 and 29

Under review

supplemental table showing all included studies WITH PID

Obs	Testing Approach	Author(s)	Year	title	source	WHO location	Key Population Category	% male	Uptake	New Positivity	Linkage	ART initiation	Retention in care	VL suppression
1	Home-based HTS	Bell DN, Martinez J, Botwinick G, Shaw K, Walker LE, Dodds S, Sell RL, Johnson RL, Friedman LB, Sotheran JL,	2003	Case finding for HIV-positive youth: a special type of hidden population	article	AMERO		X						
2	Home-based HTS	Were WA, Mermin JH, Wamai N, Awor AC, Bechange S, Moss S, Solberg P, Downing RG, Coutinho A, Bunnell RE.	2006	Undiagnosed HIV infection and couple HIV discordance among household members of HIV-infected people receiving antiretroviral	article	AFRO		X						
3	Home-based HTS	[illegible] B, Wanyenze R, Nuwaha F, Mugisha B, Coutinho A, Bunnell	2009	[illegible] effectiveness of four HIV counseling and testing	article	AFRO		X						
4	Home-based HTS	Menzies N, Abang B, Wanyenze R, Nuwaha F, Mugisha B, Coutinho A, Bunnell R, Mermin J, Blandford JM.	2009	The costs and effectiveness of four HIV counseling and testing strategies in Uganda	article	AFRO		X						
5	Home-based HTS	Negin J, Wariero J, Mutuo P, Jan S, Pronyk P.	2009	Feasibility, acceptability and cost of home-based HIV testing in	article	AFRO		X						

Under review

6	Home-based HTS	Obare F, Fleming P, Anglewicz P,Thornton R, Martinson F, Kapatuka A, Poulin M, Watkins S, Kohler	2009	Acceptance of repeat population-based voluntary counselling and testing for HIV in rural Malawi	article	AFRO		X						
7	Home-based HTS	Lugada E, Levin J, Abang B, Mermin J, Mugalanzi E, Namara G, Gupta S, Grosskurth H, Jaffar S, Coutinho A, Bunnell R.	2010	Comparison of home and clinic-based HIV testing among household members of persons taking antiretroviral therapy in Uganda: results from a	article	AFRO		X						
8	Home-based HTS	Mutale W, Michelo C, Jurgensen M, Fylkesnes K.	2010	Home-based voluntary HIV counselling and testing found highly acceptable and to reduce	article	AFRO		X						
9	Home-based HTS	Tumwesigye E, Wana G, Kasasa S, Muganzi E, Nuwaha F.	2010	High uptake of home-based, district-wide, HIV counseling and testing in	article	AFRO		X	X					
10	Home-based HTS	Vreeman RC, Nyandiko WM, Braitstein P, Were MC, Ayaya SO, Ndege SK, Wiehe SE.	2010	Acceptance of HIV testing for children ages 18 months to 13 years identified through voluntary, home-based HIV counseling and testing in	article	AFRO		X						

11	Home-based HTS	Mulogo EM, Abdulaziz AS, Guerra R, Baine SO.	2011	Facility and home based HIV Counseling and Testing: a comparative analysis of uptake of services by rural communities in southwestern	article	AFRO		X						
12	Home-based HTS	Maheswaran H, Thulare H, Stanistreet D, Tanser F, Newell ML.	2012	Starting a home and mobile HIV testing service in a rural area of South Africa	article	AFRO		X						
13	Home-based HTS	Maheswaran H, Thulare H, Stanistreet D, Tanser F, Newell ML.	2012	Starting a home and mobile HIV testing service in a rural area of South Africa	article	AFRO		X						
14	Home-based HTS	Dalal W, Feikin DR, Amolloh M, Ransom R, Burke H, Lugalia F, Ouma A, Laserson KF, Mermin J, Breiman	2013	Home-based HIV testing and counseling in rural and urban Kenyan communities	article	AFRO		X		X				
15	Home-based HTS	Doherty T, Tabana H, Jackson D, Naik R, Zembe W, Lombard C, Swanevelder S, Fox MP, Thorson A, Ekstrom AM,	2013	Effect of home based HIV counselling and testing intervention in rural South Africa: cluster randomised trial	article	AFRO		X						

16	Home-based HTS	Fylkesnes K, Sandoy IF, Jurgensen M, Chipimo PJ, Mwangala S, Michelo C.	2013	Strong effects of home-based voluntary HIV counselling and testing on acceptance and equity: a cluster randomised trial in Zambia	article	AFRO		X	X					
17	Home-based HTS	Helleringer S, Mkandawire J, Reniers G, Kalilani-Phiri L, Kohler HP.	2013	Should home-based HIV testing and counseling services be offered periodically in programs of ARV treatment as prevention? A case study in	article	AFRO		X		X				
18	Home-based HTS	Medley A, Ackers M, Amolloh M, Owuor P,Muttai H, Audi B, Sewe M, Laserson K.	2013	Early uptake of HIV clinical care after testing HIV-positive during home-based testing and counseling in western Kenya	article	AFRO					X	X		
19	Home-based HTS	Osoti AO, John-Stewart G, Kiarie J, Richardson B, Kinuthia J, Krakowiak D, Farquhar C.	2014	Home visits during pregnancy enhance male partner HIV counselling and testing in Kenya: a randomized	abstract	AFRO		X						

review

supplemental table showing all included studies WITH PID

20	Home-based HTS	van Rooyen H, Barnabas RV, Baeten JM, Phakathi Z, Joseph P, Krows M, Hong T, Murnane PM, Hughes J, Celum C.	2013	High HIV testing uptake and linkage to care in a novel program of home-based HIV counseling and testing with facilitated referral in	article	AFRO		X						
21	Home-based HTS	Okiria AG, Okui O, Dutki M, Baryamutuma R, Nuwagaba CK, Kansiime E, Ojamuge G,Mugweri J, Fleuret J, King R, Bazeyo W, Lindan	2014	HIV incidence and factors associated with seroconversion in a rural community home based counseling and testing program	article	AFRO		X						
22	Home-based HTS	Mark J.; Kinuthia J.; Osoti A.; Gone M.; Asila V.; Parikh S.; Krakowiak D.; Betz B.; Richardson B.; Roxby A.; Farquhar C.	2015	Male partner acceptance of home-based syphilis and HIV testing offered to couples during pregnancy	abstract	AFRO		X	X					
23	Home-based HTS	Parker LA, Jobanputra K, Rusike L, Mazibuko S, Okello V, Kerschberger B, Jouquet G, Cyr J,	2015	Feasibility and effectiveness of two community-based HIV testing models in rural Swaziland	article	AFRO		X						

er review

24	Home-based HTS	Chamie G, Clark TD, Kabami J, Kadede K, Ssemmondo E, Steinfeld R, Lavoy G, Kwarisiima D, Sang N, Jain V, Thirumurthy H, Liegler T, Balzer LB, Petersen ML, Cohen CR, Bukusi EA, Kamya MR,	2016	A hybrid mobile HIV testing approach for population-wide HIV testing in rural East Africa: an observational study	article	AFRO		X						
25	Home-based HTS	Chang LW, Grabowski MK, Ssekubugu R, Nalugoda F, Kigozi G, Nantume B, Lessler J, Moore SM, Quinn TC, Reynolds SJ, Gray RH,	2016	Heterogeneity of the HIV epidemic in agrarian, trading, and fishing communities in Rakai, Uganda: an observational epidemiological	article	AFRO		X						
26	Home-based HTS	Krakowiak D.; Kinuthia J.; Osoti A.; Asila V.; Ann Gone M.; Mark J.; Sharma M.; Barnabas R.V.; Farquhar C.	2016	Home-Based HIV Testing Among Pregnant Couples Increases Partner Testing and	article	AFRO		X	X					
27	Home-based HTS	Phiri M.M.; Shanaube K.; Floyd S.; Sakala E.; Besa S.; Griffith S.; Ayles H.	2016	Does a male chip increase uptake of HIV testing by men? Lessons from HPTN 071 study	abstract	AFRO		X	X					

28	Home-based HTS	Phiri M.M.; Shanaube K.; Floyd S.; Sakala E.; Besa S.; Griffith S.; Ayles H.	2016	Does a male chip increase uptake of HIV testing by men? Lessons from HPTN 071 study	abstract	AFRO		X	X					
29	Home-based HTS	Asiimwe S, Ross JM, Arinaitwe A, Tumusiime O, Turyamureeba B, Roberts DA, O'Malley G, Barnabas RV.	2017	Expanding HIV testing and linkage to care in southwestern Uganda with community health extension workers	article	AFRO		X						
30	Home-based HTS	Bogart LM, Wagner GJ, Musoke W, Naigino R, Linnemayr S, Maistrellis E, Klein DJ, Jumamil RB, Mukasa B, Bassett IV, Giordano TP, Wanyenze RK.	2017	A Comparison of Home-Based Versus Outreach Event-Based Community HIV Testing in Ugandan Fisherfolk	article	AFRO		X						
31	Home-based HTS	Brunie A, Mucheri PNW, Akol A, Chen M, Mercer SJ, Petruney T.	2017	Integrating Family Planning and HIV Services at the Community Level: Formative Assessment with Village	article	AFRO		X						
32	Home-based HTS	Floyd S.; Phiri M.; Schaap A.; Macleod D.; Shanaube K.; Griffith S.; Beyers N.; Hayes	2017	Art coverage after 2 years of a utt intervention in Zambia: Findings from HPTN071	abstract	AFRO		X		X		X		

Under rev

33	Home-based HTS	Geoffroy E, Schell E, Jere J, Khozomba N.	2017	Going door-to-door to reach men and young people with HIV testing services to achieve the 90-90-90 treatment	article	AFRO		X	X					
34	Home-based HTS	Hayes R, Floyd S, Schaap A, Shanaube K, Bock P, Sabapathy K, Griffith S, Donnell D, Piwowar-Manning E, El-Sadr W, Beyers N, Ayles H, Fidler S; HPTN 071 (PopART) Study Team..	2017	A universal testing and treatment intervention to improve HIV control: One-year results from intervention communities in Zambia in the HPTN 071 (PopART)	article	AFRO		X						
35	Home-based HTS	Justman J, Reed JB, Bicego G, Donnell D, Li K, Bock N, Koler A, Philip NM, Mlambo CK, Parekh BS, Duong YT, Ellenberger DL, El-Sadr WM,	2017	Swaziland HIV Incidence Measurement Survey (SHIMS): a prospective national cohort study	article	AFRO		X						
36	Home-based HTS	Mark J, Kinuthia J, Roxby AC, Krakowiak D, Osoti A, Richardson BA, Gone MA, Asila V, Parikh S, Farquhar C.	2017	Uptake of Home-Based Syphilis and Human Immunodeficiency Virus Testing Among Male Partners of Pregnant Women in	article	AFRO		X	X					

Under r

37	Home-based HTS	Mark J, Kinuthia J, Roxby AC, Krakowiak D, Osoti A, Richardson BA, Gone MA, Asila V, Parikh S, Farquhar C.	2017	Uptake of Home-Based Syphilis and Human Immunodeficiency Virus Testing Among Male Partners of Pregnant Women in	article	AFRO		X	X					
38	Home-based HTS	Ogirima, F., Muhammed, R., Agada, G., Christopher Izere, P., Abutu, I., Udeh, E., Jwanle, P., Ashie, M., Ameh, B., Ujah, J., & Oyeledun, B.	2017	Bridging the HIV treatment gap using a door to door strategy: experience from the community care program in Benue state Nigeria	abstract	AFRO		X						
39	Home-based HTS	Oluoch P, Orwa J, Lugalia F, Mutinda D, Gichangi A, Oundo J, Karama M, Nganga Z, Galbraith J.	2017	Application of psychosocial models to Home-Based Testing and Counseling (HBTC) for increased uptake and household coverage in a large informal urban	article	AFRO		X	X					
40	Home-based HTS	Shanaube K, Schaap A, Chaila MJ, Floyd S, Mackworth-Young C, Hoddinott G, Hayes R, Fidler S, Ayles H; HPTN 071 (PopART) Study Team..	2017	Community intervention improves knowledge of HIV status of adolescents in Zambia: findings from HPTN 071-PopART for youth study	article	AFRO		X	X					

41	Home-based HTS	Shanaube K, Schaap A, Floyd S, Phiri M, Griffith S, Chaila J, Bock P, Hayes R, Fidler S, Ayles H; HPTN 071 (PopART) Study Team..	2017	What works - reaching universal HIV testing: lessons from HPTN 071 (PopART) trial in Zambia	article	AFRO		X	X	X				
42	Home-based HTS	Shanaube K.; Chaila M.J.; Macleod D.; Schaap A.; Floyd S.; Jani C.; Hoddinott G.; Hayes R.; Fidler S.;	2017	Community intervention improves adolescent HIV status knowledge: HPTN 071 study	abstract	AFRO		X						
43	Home-based HTS	Floyd S.; Ayles H.; Schaap A.; Shanaube K.; MacLeod D.; Phiri M.; Griffith S.; Bock P.; Beyers N.; Fidler S.; Hayes R.	2018	Towards 90-90: Findings after two years of the HPTN 071 (PopART) cluster-randomized trial of a universal testing-and-treatment intervention in	article	AFRO		X	X	X				
44	Home-based HTS	Muchedzi A.; Mahachi N.; Moga T.; Tafuma T.; Mawora P.; Harbick D.; Nyagura T.; Reichert K.	2018	Improving technical efficiency: Reaching first 90 through community index HIV sexual network testing in Zimbabwe. The case of FHI 360	abstract	AFRO		X						

45	Home-based HTS	O'Laughlin K.N.; He W.; Greenwald K.E.; Kasozi J.; Chang Y.; Mulogo E.; Faustin Z.M.; Njogu P.; Walensky R.P.; Bassett I.V.	2018	Feasibility and acceptability of home-based HIV testing among refugees: A pilot study in Nakivale refugee settlement in	article	AFRO		X						
46	Home-based HTS	Olawore O.M.; Tobian A.; Nalugoda F.; Gray R.H.; Wawer M.; Ssekubugu R.; Santelli J.; Chang L.W.; Serwadda D.;	2018	Migration, gender, and HIV incidence in Rakai, Uganda	article	AFRO		X						
47	Home-based HTS	Roland M.; Block L.; Bachanas P.; Alwano M.G.; Abrams W.; Wirth K.; Gaolathe T.; Makhema J.; Mmalane M.; Lockman S.; El-Halabi S.; Moore J.	2018	Home-based testing identifies more previously undiagnosed older men than mobile testing in Botswana	abstract	AFRO		X		X				
48	Home-based HTS	Ruzagira E, Baisley K, Kamali A, Grosskurth H.	2018	Factors associated with uptake of home-based HIV counselling and testing and HIV care services among identified HIV-positive persons in Masaka,	article	AFRO		X	X					

49	Home-based HTS	Sinha, P.; Moll, A. P.; Brooks, R. P.; Deng, Y.-H.; Shenoi, S. V.	2018	Synergism between diabetes and human immunodeficiency virus in increasing the	article	AFRO		X						
50	Home-based HTS	Tafuma T.A.; Mahachi N.; Dziwa C.; Marowa P.; Moga T.; Chimbidzikai T.; Muchedzi A.; Nyagura T.; Mpofu M.	2018	Time taken to link newly identified HIV positive clients to care following a home-base index case HIV testing: Experience from two provinces in	article.	AFRO								
51	Index/partner notification	Suggaravetsiri P, Yanai H, Chongsuvivatwong V, Naimpasan O, Akarasewi P.	2003	Integrated counseling and screening for tuberculosis and HIV among household contacts of tuberculosis patients in an endemic area of HIV infection: Chiang Rai, Thailand	article	SEARO		X						
52	Index/partner notification	DiCarlo A, Zerbe A, Peters ZJ, Frederix K, Nkonyana JP, Mantell JE, Remien RH, El-Sadr WM.	2017	Use of Index Patients to Enable Home-Based Testing in Lesotho	article	AFRO		X		X				

53	Index/partner notification	Mahachi, N., Muchedzi, A., Moga, T., Tapfuma, T., Dziwa, C., Chimbidzikayi, T., Gonouya, S., Chakubili, O., & Torpey, K.	2017	High yields attained through HIV household index case testing in Zimbabwe: the case of the FHI 360 Zimbabwe HIV care and	abstract	AFRO		X						
54	Outreach HTS	Parker LA, Jobanputra K, Rusike L, Mazibuko S, Okello V, Kerschberger B, Jouquet G, Cyr J,	2015	Feasibility and effectiveness of two community-based HIV testing models in rural Swaziland	article	AFRO								
55	Outreach HTS	DiFranceisco W, Holtgrave DR, Hoxie N, Reiser WJ, Resenhoeft R, Pinkerton SD, Vergeront, J.	1998	HIV seropositivity rates in outreach-based counseling and testing services:	article	AMERO	Mixed populations	X						
56	Outreach HTS	Keenan, P. A., & Keenan, J. M.	2001	Rapid HIV Testing in Urban Outreach: A Strategy for Improving Posttest Counseling	article	AMERO	MSM	X						
57	Outreach HTS	Keenan, P. A., & Keenan, J. M.	2001	Rapid HIV Testing in Urban Outreach: A Strategy for Improving Posttest Counseling	article	AMERO	Mixed populations	X						
58	Outreach HTS	Liebman J, Pat Lamberti M, Altice F.	2002	Effectiveness of a mobile medical van in providing screening services for	article	AMERO		X						

59	Outreach HTS	Bell DN, Martinez J, Botwinick G, Shaw K, Walker LE, Dodds S, Sell RL, Johnson RL, Friedman LB, Sotheran JL,	2003	Case finding for HIV-positive youth: a special type of hidden population	article	AMERO		X						
60	Outreach HTS	Bell DN, Martinez J, Botwinick G, Shaw K, Walker LE, Dodds S, Sell RL, Johnson RL, Friedman LB, Sotheran JL,	2003	Case finding for HIV-positive youth: a special type of hidden population	article	AMERO		X						
61	Outreach HTS	Kahn RH, Moseley KE, Thilges JN, Johnson G, Farley TA.	2003	Community-based screening and treatment for STDs: results from a mobile clinic	article	AMERO		X						
62	Outreach HTS	Bradshaw, C. S., Pierce, L. I., Tabrizi, S. N., Fairley, C. K., & Garland, S. M.	2005	Screening injecting drug users for sexually transmitted infections and blood borne viruses using street outreach and self	article	WPRO	People who inject/use drugs	X						
63	Outreach HTS	Liang TS, Erbelding E, Jacob CA, Wicker H, Christmyer C, Brunson S, Richardson D, Ellen JM.	2005	Rapid HIV testing of clients of a mobile STD/HIV clinic	article	AMERO	Mixed populations	X						

**64**	Outreach HTS	Liang TS, Erbelding E, Jacob CA, Wicker H, Christmyer C, Brunson S, Richardson D, Ellen JM.	2005	Rapid HIV testing of clients of a mobile STD/HIV clinic	article	AMERO		X						
**65**	Outreach HTS	Liang TS, Erbelding E, Jacob CA, Wicker H, Christmyer C, Brunson S, Richardson D, Ellen JM.	2005	Rapid HIV testing of clients of a mobile STD/HIV clinic	article	AMERO		X						
**66**	Outreach HTS	Lister, N. A., Smith, A., Tabrizi, S. N., Garland, S., Hayes, P., & Fairley, C. K.	2005	Comprehensive clinical care on-site in men-only saunas: confidential STI/HIV screening outreach clinic	article	WPRO	MSM	X						
**67**	Outreach HTS	Spielberg, F., Branson, B. M., Goldbaum, G. M., Lockhart, D., Kurth, A., Rossini, A., & Wood, R. W.	2005	Choosing HIV Counseling and Testing Strategies for Outreach Settings: A Randomized	article	AMERO	People who inject/use drugs	X						
**68**	Outreach HTS	Spielberg, F., Branson, B. M., Goldbaum, G. M., Lockhart, D., Kurth, A., Rossini, A., & Wood, R. W.	2005	Choosing HIV Counseling and Testing Strategies for Outreach Settings: A Randomized	article	AMERO	People who inject/use drugs	X		X				
**69**	Outreach HTS	Galvan, F. H., Bluthenthal, R. N., Ani, C., & Bing, E. G.	2006	Increasing HIV Testing Among Latinos by Bundling HIV Testing with Other Tests	article	AMERO	MSM	X	X					

Under re

70	Outreach HTS	Morin SF, Khumalo-Sakutukwa G, Charlebois ED, Routh J, Fritz K, Lane T, Vaki T, Fiamma A, Coates TJ.	2006	Removing barriers to knowing HIV status: same-day mobile HIV testing in Zimbabwe	article	AFRO		X						
71	Outreach HTS	Rose, V. J., Raymond, H. F., Kellogg, T. A., & McFarland, W.	2006	Assessing the feasibility of harm reduction services for MSM: the late night breakfast buffet study	article	AMERO	MSM	X						
72	Outreach HTS	Bucher, J. B., Thomas, K. M., Guzman, D., Riley, E., Dela Cruz, N., & Bangsberg, D. R.	2007	Community-based rapid HIV testing in homeless and marginally housed adults in San Francisco	article	AMERO	Mixed populations	X						
73	Outreach HTS	Bucher JB, Thomas KM, Guzman D, Riley E, Dela Cruz N, Bangsberg DR.	2007	Community-based rapid HIV testing in homeless and marginally housed adults in San Francisco	article	AMERO		X						
74	Outreach HTS	Kawichai S, Celentano DD, Chariyalertsak S, Visrutaratna S, Short O, Ruangyuttikarn C, Chariyalertsak C, Genberg B, Beyrer	2007	Community-based voluntary counseling and testing services in rural communities of Chiang Mai Province,	article	SEARO		X						

Under r

75	Outreach HTS	Bingham, T. A., Secura, G. M., Behel, S. K., Bunch, J. G., Simon, P. A., & MacKellar, D. A.	2008	HIV Risk Factors Reported by Two Samples of Male Bathhouse Attendees in Los Angeles, California 2001-	article	AMERO	MSM	X						
76	Outreach HTS	Arumainayagam, J., Grimshaw, R., Acharya, S., Chandramani, S., Morrall, I. A., & Pugh, R. N.	2009	Value of targeting at-risk populations at outreach venues: findings from a local	article	EURO	MSM	X						
77	Outreach HTS	Daskalakis, D., Silvera, R., Bernstein, K., Stein, D., Hagerty, R., Hutt, R., Maillard, A., Borkowsky, W., Aberg, J.,	2009	Implementation of HIV Testing at 2 New York City Bathhouses: From Pilot to Clinical Service	article	AMERO	MSM	X			X			
78	Outreach HTS	de la Fuente L, Delgado J, Hoyos J, Belza MJ, Alvarez J, Gutierrez J, Neira-Leon M, Suraez M, Madrid	2009	Increasing early diagnosis of HIV through rapid testing in a street outreach program in Spain	article	EURO		X						
79	Outreach HTS	Chirawu P, Langhaug L, Mavhu W, Pascoe S, Dirawo J, Cowan F.	2010	Acceptability and challenges of implementing voluntary counselling and testing (VCT) in rural Zimbabwe: evidence from the Regai Dzive Shiri Project	article	AFRO		X						

80	Outreach HTS	Grabbe KL, Menzies N, Taegtmeyer M, Emukule G, Angala P, Mwega I, Musango G, Marum E.	2010	Increasing access to HIV counseling and testing through mobile services in Kenya: strategies, utilization, and cost-	article	AFRO		X						
81	Outreach HTS	Grabbe KL, Menzies N, Taegtmeyer M, Emukule G, Angala P, Mwega I, Musango G, Marum E.	2010	Increasing access to HIV counseling and testing through mobile services in Kenya: strategies, utilization, and cost-	article	AFRO		X						
82	Outreach HTS	Lahuerta, M., Sabidó, M., Giardina, F., Hernández, G., Palacios, J.F., Ortiz, R., Fernández, V.H. and Casabona, J.	2010	Comparison of users of an HIV/syphilis screening community-based mobile van and traditional voluntary counselling and testing sites in	article	AMERO	Mixed populations	X						
83	Outreach HTS	Lugada E, Millar D, Haskew J, Grabowsky M, Garg N, Vestergaard M, Kahn JG, Muraguri N, Mermin J.	2010	Rapid implementation of an integrated large-scale HIV counseling and testing, malaria, and diarrhea prevention campaign in rural Kenya	article	AFRO		X						

er review

**84**	Outreach HTS	Brady M, Harrison C, Warriner J, Skinner C, Larbalestier N, Ward P.	2011	Community HIV testing: the feasibility and acceptability of assertive outreach and community testing to reduce the late	article	EURO		X						
**85**	Outreach HTS	Govindasamy D, van Schaik N, Kranzer K, Wood R, Mathews C, Bekker LG.	2011	Linkage to HIV care from a mobile testing unit in South Africa by different CD4	article	AFRO		X						
**86**	Outreach HTS	Kranzer K, Govindasamy D, van Schaik N, Thebus E, Davies N, Zimmermann M, Jeneker S, Lawn S, Wood R, Bekker LG.	2011	Incentivized recruitment of a population sample to a mobile HIV testing service increases the yield of newly diagnosed cases, including those in need of antiretroviral therapy	article	AFRO		X						
**87**	Outreach HTS	Kranzer K, Govindasamy D, van Schaik N, Thebus E, Davies N, Zimmermann M, Jeneker S, Lawn S, Wood R, Bekker LG.	2011	Incentivized recruitment of a population sample to a mobile HIV testing service increases the yield of newly diagnosed cases, including those in need of antiretroviral therapy	article	AFRO		X						

Under review

88	Outreach HTS	Ostermann J, Reddy EA, Shorter MM, Muiruri C, Mtalo A, Itemba DK, Njau B, Bartlett JA, Crump JA, Thielman NM.	2011	Who tests, who doesn't, and why? Uptake of mobile HIV counseling and testing in the Kilimanjaro Region of	article	AFRO		X						
89	Outreach HTS	Baisley K, Doyle AM, Changalucha J, Maganja K, Watson-Jones D, Hayes R, Ross D.	2012	Uptake of voluntary counselling and testing among young people participating in an HIV prevention trial: comparison of opt-out and opt-	article	AFRO		X						
90	Outreach HTS	Baisley K, Doyle AM, Changalucha J, Maganja K, Watson-Jones D, Hayes R, Ross D.	2012	Uptake of voluntary counselling and testing among young people participating in an HIV prevention trial: comparison of opt-out and opt-	article	AFRO		X						
91	Outreach HTS	Granich R, Muraguri N, Doyen A, Garg N, Williams BG.	2012	Achieving universal access for human immunodeficien cy virus and tuberculosis: potential prevention impact of an integrated multi-disease prevention campaign in	article	AFRO		X						

92	Outreach HTS	Hood JE, MacKellar D, Spaulding A, Nelson R, Mosiakgabo B, Sikwa B, Puso I, Raats J, Loeto P, Alwano MG, Monyatsi B.	2012	Client characteristics and gender-specific correlates of testing HIV positive: a comparison of standalone center versus mobile outreach HIV testing and counseling in Botswana	article	AFRO		X						
93	Outreach HTS	Celentano D, Srithanaviboonchai K, Wichajarn M,	2012	Accept (HPTN 043) HIV/AIDS community mobilization	article	SEARO		X						
94	Outreach HTS	Ngiazi MD, van Schaik N, Kranzer K, Lawn SD, Wood	2012	An incentivized HIV counseling and testing	article	AFRO		X		X				
95	Outreach HTS	Schaik N, Kranzer K, Lawn SD, Wood R, Bekker LG.	2012	HIV counseling and testing program	article	AFRO		X		X				
96	Outreach HTS	Van Rooyen H, McGrath N, Chirowodza A, Joseph P,	2013	Mobile VCT: reaching men and young people in urban	article	AFRO		X						
97	Outreach HTS	McCoy SI, Shiu K, Martz TE, Smith CD, Mattox L, Gluth DR, Murgai	2013	Improving the efficiency of HIV testing with peer recruitment,	article	AMERO		X						
98	Outreach HTS	Cawley C, Wringe A, Slaymaker E, Todd J, Michael D, Kumugola Y,	2014	The impact of voluntary counselling and testing services	article	AFRO		X						
99	Outreach HTS	Balbuena S, de la Fuente L, Hoyos J, Rosales-Statkus	2014	Highly visible street-based HIV rapid testing: is it an	article	EURO		X		X				
##	Outreach HTS	Fernandez-Balbuena S, de la Fuente L, Hoyos J, Rosales-Statkus	2014	Highly visible street-based HIV rapid testing: is it an	article	EURO	MSM	X		X				

For Peer Review

##	Outreach HTS	Ifekandu, C., Suleiman, A., & Aniekwe, O.	2014	The cost-effectiveness in the use of HIV counselling and	article	AFRO	MSM	X								
##	Outreach HTS	Kakalou, E., Papastamopoulos, V., Ioannidis, P., Papanikolaou, K., Georgiou, O., & Skoutelis, A.	2014	Early HIV diagnosis through use of rapid diagnosis test (RDT) in the community and direct link to HIV care: a pilot project for vulnerable populations in Athens, Greece	article	EURO	Mixed populations	X								
##	Outreach HTS	Lipsitz, M.C., Segura, E.R., Castro, J.L., Smith,	2014	Bringing testing to the people - benefits of	article	AMERO	MSM & Transgender	X								
##	Outreach HTS	Mabuto T, Latka MH, Kuwane B, Churchyard GJ,	2014	Four models of HIV counseling and testing:	article	AFRO		X								
##	Outreach HTS	Mabuto T, Latka MH, Kuwane B, Churchyard GJ, Charalambous S, Hoffmann CJ.	2014	Four models of HIV counseling and testing: utilization and test results in South Africa	article	AFRO		X								
##	Outreach HTS	Mdodo, R., Thomas, P. E., Walker, A., Chavez, P., Ethridge, S., Oraka, E., & Sutton, M. Y.	2014	Rapid HIV Testing at Gay Pride Events to Reach Previously Untested MSM:	article	AMERO	MSM	X								
##	Outreach HTS	Zhang D, Meng S, Xu P, Lu H, Zhuang M, Wu G, Liu Y, Pan X, Yan H, Chen X, Fan L, Li C, Fu X, Qi J, Han L, Ma F, Lv F, Sun	2014	Experience of offering HIV rapid testing to at-risk patients in community health centers in eight Chinese	article	WPRO		X								

##	Outreach HTS	Adebajo S, Eluwa G, Njab J, Oginni A, Ukwuije F, Ahonsi B, Lorenc T.	2015	Evaluating the effect of HIV prevention strategies on	article	AFRO	Mixed populations	X	X					
##	Outreach HTS	Adebajo S, Eluwa G, Njab J, Oginni A, Ukwuije F, Ahonsi B, Lorenc T.	2015	Evaluating the effect of HIV prevention strategies on	article	AFRO	Mixed populations	X	X					
##	Outreach HTS	Bassett IV, Regan S, Mbonambi H, Blossom J, Bogan S, Bearnot B, Robine M, Walensky RP, Mhlongo B, Freedberg KA, Thulare H, Losina E.	2015	Finding HIV in hard to reach populations: mobile HIV testing and geospatial mapping in Umlazi township, Durban, South	article	AFRO		X						
##	Outreach HTS	Belza MJ, Hoyos J, FernÃ¡ndez-Balbuena S, Diaz A, Bravo MJ, de la Fuente L; Madrid HIV rapid testing group..	2015	Assessment of an outreach street-based HIV rapid testing programme as a strategy to promote early diagnosis: a comparison with two surveillance systems in Spain, 2008-	article	EURO		X						
##	Outreach HTS	Belza MJ, Hoyos J, Fernández-Balbuena S, Diaz A, Bravo MJ, de la Fuente L; Madrid HIV rapid testing group..	2015	Assessment of an outreach street-based HIV rapid testing programme as a strategy to promote early diagnosis: a comparison with two surveillance systems in Spain, 2008-	article	EURO	MSM	X						

##	Outreach HTS	Darin KM, Klepser ME, Klepser DE, Klepser SA, Reeves A, Young M, Scarsi KK.	2015	Pharmacist-provided rapid HIV testing in two community pharmacies	article .	AMERO		X						
##	Outreach HTS	Fernandez-Balbuena S, Belza MJ, Zulaica D, Martinez JL, Marcos H, Rifa B, Arrillaga A, de la Fuente L, Hoyos J; Working Group.	2015	Widening the Access to HIV Testing: The Contribution of Three In-Pharmacy Testing Programmes in	article	EURO		X						
##	Outreach HTS	Fernandez-Balbuena S, Marcos H, Perez-	2015	The rapid test in Spanish pharmacies: a	article	EURO		X		X				
##	Outreach HTS	Marcus U, Ort J, Grenz M, Eckstein K, Wirtz K, Wille A.	2015	Risk factors for HIV and STI diagnosis in a community-based HIV/STI testing and counselling site for men having sex with men (MSM) in a large German city in	article	EURO	MSM	X						
##	Outreach HTS	Parker LA, Jobanputra K, Rusike L, Mazibuko S, Okello V, Kerschberger B, Jouquet G, Cyr J,	2015	Feasibility and effectiveness of two community-based HIV testing models in rural Swaziland	article	AFRO		X						

Under review

supplemental table showing all included studies WITH PID

##	Outreach HTS	Wood M, Ellks R, Grobicki M.	2015	Outreach sexual infection screening and postal tests in men who have sex with men: are they comparable to clinic screening?	article	EURO	MSM	X	X					
##	Outreach HTS	van Zyl MA, Brown LL, Pahl K.	2015	Using a call center to encourage linkage to care following mobile HIV counseling and testing	article	AFRO								
##	Outreach HTS	Chamie G, Clark TD, Kabami J, Kadede K, Ssemmondo E, Steinfeld R, Lavoy G, Kwarisiima D, Sang N, Jain V, Thirumurthy H, Liegler T, Balzer LB, Petersen ML, Cohen CR, Bukusi EA, Kamya MR,	2016	A hybrid mobile approach for population-wide HIV testing in rural east Africa: an observational study	article	AFRO		X						
##	Outreach HTS	Arevalo, A. L., Duran, A., Carrizo, E., Betti, L., Marachlian, L., Vulcano, F., Nan, M., Carones, M. L., Serantes, D., Carrozzi, B., Vulcano, S., Orge, P., Hirsch, C., Minissale, G.,	2017	Free HIV tests on public spaces: a strategy which allows easy access to diagnose in the Autonomous City of Buenos Aires (CABA)`	abstract	AMERO		X						

##	Outreach HTS	Camacho-Gonzalez A.; Gillespie S.; Thomas-Seaton L.; Frieson K.; Hussen S.A.; Murray A.; Gaul Z.; Sutton M.; Graves C.; Chakraborty R.	2017	The Metropolitan Atlanta community adolescent rapid testing initiative study: closing the gaps in HIV care among youth in Atlanta,	article	AMERO								
##	Outreach HTS	Castel AD, Kuo I, Mikre M, Young T, Haddix M, Das S, Maugham G, Reisen C.	2017	Feasibility of Using HIV Care-Continuum Outcomes to Identify Geographic Areas for Targeted HIV Testing	article	AMERO		X						
##	Outreach HTS	Chamie G.; Schaffer E.; Ndyabakira A.; Emperador D.; Kwarisiima D.; Havlir D.V.; Kahn J.; Kamya M.R.;	2017	A randomized trial of novel strategies to incentivize HIV testing among men in Uganda	abstract	AFRO		X	X					
##	Outreach HTS	Daniels J, Komarek A, Forgreive B, Pahl K, Stafford S, Bruns LC, Coates T.	2017	Shout-It-Now: A Mobile HCT Model Employing Technology and Edutainment in	article	AFRO		X						
##	Outreach HTS	Ezeanolue EE, Obiefune MC, Yang W, Ezeanolue CO, Pharr J, Osuji A, Ogidi AG, Hunt AT, Patel D, Ogedegbe G, Ehiri JE.	2017	What do You Need to Get Male Partners of Pregnant Women Tested for HIV in Resource Limited Settings? The Baby Shower	article	AFRO		X	X					

##	Outreach HTS	Khawcharoenporn T, Apisarnthanarak A, Phanuphak N.	2017	Active targeted HIV testing and linkage to care among men who have sex with men attending a gay sauna in Thailand	article	SEARO	MSM	X	X		X			
##	Outreach HTS	Knoblauch AM, Divall MJ, Owuor M, Nduna K, Ng'uni H, Musunka G, Pascall A, Utzinger J, Winkler MS.	2017	Experience and lessons from health impact assessment guiding prevention and control of HIV/AIDS in a copper mine project, northwestern Zambia	article	AFRO		X						
##	Outreach HTS	Krisintu, P., Avery, M., Sattayapanich, T., Arunmanakul, A., Janyam, S., & Linjongrat, D.	2017	Making mobile HIV testing available for high-risk MSM in saunas	abstract	SEARO	MSM	X						
##	Outreach HTS	Mutch AJ, Lui CW, Dean J, Mao L, Lemoire J, Debattista J, Howard C, Whittaker A, Fitzgerald L.	2017	Increasing HIV testing among hard-to-reach groups: examination of RAPID, a community-based testing service in Queensland,	article	WPRO		X						
##	Outreach HTS	Ngunu-Gituathi, C., Omai, J., Kongin, H., Ogaro, T., & Ochola, S.	2017	Fast tracking the HIV response in Nairobi city by targeted HIV testing of key populations, Kenya, 2015.	abstract	AFRO	Mixed populations	X						

##	Outreach HTS	Okoko, N. A., Guze, M. A., Ndolo, S., Nyanaro, G., Bukusi, E. A., Cohen, C. R., Penner, J., & Kulzer, J. L.	2017	Toward the first 90: identifying and testing younger populations for HIV at community outreach events	abstract	AFRO		X						
##	Outreach HTS	SEARCH Collaboration..	2017	Evaluating the feasibility and uptake of a community-led HIV testing and multi-disease health campaign in rural Uganda	article	AFRO		X						
##	Outreach HTS	Sibanda EL, Tumushime M, Mufuka J, Mavedzenge SN, Gudukeya S, Bautista-Arredondo S, Hatzold K, Thirumurthy H, McCoy SI, Padian N, Copas A, Cowan FM.	2017	Effect of non-monetary incentives on uptake of couples' counselling and testing among clients attending mobile HIV services in rural Zimbabwe: a cluster-randomised trial	article	AFRO		X						
##	Outreach HTS	Sibanda EL, Tumushime M, Mufuka J, Mavedzenge SN, Gudukeya S, Bautista-Arredondo S, Hatzold K, Thirumurthy H, McCoy SI, Padian N, Copas A, Cowan FM.	2017	Effect of non-monetary incentives on uptake of couples' counselling and testing among clients attending mobile HIV services in rural Zimbabwe: a cluster-randomised trial	article	AFRO		X						

##	Outreach HTS	Smyrnov, P., Williams, L., Korobchuk, A., Sazonova, Y., Nikolopoulos, Skaathun, B., Schneider, J., & Friedman, S. R.	2017	Social network approaches to locating undiagnosed HIV cases are more effective than RDS recruitment or outreach models	abstract	EURO	People who inject/use drugs	X							
##	Outreach HTS	van Niekerk, M., Draper, H., & Meehan, S.-A.	2017	Can STI screening be suitably integrated into community-based HIV testing services for men in Cape	abstract	AFRO		X							
##	Outreach HTS	Adetunji AA, Kuti MA, Audu RA, Muyibi SA, Imhansoloeva M, Mosuro OA, Solanke EA, Akpa OM, Irabor AE, Ladipo M, Berzins B, Robertson K,	2018	Discordant rapid HIV tests: lessons from a low-resource community	article	AFRO		X							
##	Outreach HTS	Bekolo CE, Yimdjo Fogue TD, Williams TD.	2018	Feasibility of integrating HIV testing into local youth development programmes in Cameroon	article	AFRO		X							
##	Outreach HTS	Geoffroy E.; Khozomba N.; Jere J.; Schell E.; Schafer T.; Goldman J.; Kabwere K.	2018	Going door-to-door to reach men and young people with HIV testing services to achieve the 90-90-90 treatment	abstract	AFRO		X							

der review

##	Outreach HTS	Herce ME, Miller WM, Bula A, Edwards JK, Sapalalo P, Lancaster KE, Mofolo I, Furtado MLM, Weir SS.	2018	Achieving the first 90 for key populations in sub-Saharan Africa through venue-based outreach: challenges and opportunities for HIV prevention based on PLACE study findings from	article	AFRO	MSM	X		X				
##	Outreach HTS	Roland M.; Block L.; Bachanas P.; Alwano M.G.; Abrams W.; Wirth K.; Gaolathe T.; Makhema J.; Mmalane M.; Lockman S.; El-Halabi S.; Moore J.	2018	Home-based testing identifies more previously undiagnosed older men than mobile testing in Botswana	abstract	AFRO		X		X				
##	Outreach HTS	Sinha, P.; Moll, A. P.; Brooks, R. P.; Deng, Y.-H.; Shenoi, S. V.	2018	Synergism between diabetes and human immunodeficiency virus in increasing the	article	AFRO		X						

Under re

##	Outreach HTS	Wasantioopapokakorn M, Manopaiboon C, Phoorisri T, Sukkul A, Lertpiriyasuwat C, Ongwandee S, Langkafah F, Kritsanavarin U, Visavakum P, Jetsawang B, Nookhai S, Kitwattanachai P, Weerawattanayotin W, Losirikul M, Yenyarsun N, Jongchotchatchawa	2018	Implementation and assessment of a model to increase HIV testing among men who have sex with men and transgender women in Thailand, 2011-2016	article	SEARO	MSM & Transgender	X						
##	Outreach HTS	Williams D.; Mackellar D.; Dlamini M.; Simelane N.; Mlambo S.; Mamba P.; Byrd J.; Mazibuko S.; Pathmanathan I.; Lukhele N.; Dube L.; Pasipamire M.; Nxumalo V.; Beyer A.; Ryan C.	2018	Rapid ART initiation and index client testing outcomes of commlink, a community-based, HIV testing, mobile HIV care, and peer-delivered, Linkage Case Management Program-	abstract	AFRO					X	X		
##	Stand-alone HT	Bell DN, Martinez J, Botwinick G, Shaw K, Walker LE, Dodds S, Sell RL , Johnson RL, Friedman LB, Sotheran JL, Siciliano C.	2003	Case finding for HIV-positive youth: a special type of hidden population	article	AMERO		X						

##	Stand-alone HT	Bailey, A. C., Roberts, J., Weatherburn, P., Hickson, F. C. I., Reid, D. S., Fisher, M., & Dean, G.	2008	Community HIV testing for men who have sex with men: results of a pilot project and comparison of service users with those testing in	article	EURO	MSM	X		X				
##	Stand-alone HT	Wringe A, Isingo R, Urassa M, Maiseli G, Manyalla R, Changalucha J, Mngara J, Kalluvya S, Zaba B.	2008	Uptake of HIV voluntary counselling and testing services in rural Tanzania: implications for effective HIV prevention and equitable	article	AFRO		X						
##	Stand-alone HT	Kimbrough, L. W., Fisher, H. E., Jones, K. T., Johnson, W., Thadiparthi, S., & Dooley, S.	2009	Accessing Social Networks With High Rates of Undiagnosed HIV Infection: The Social Networks Demonstration	article	AMERO	Mixed populations	X						
##	Stand-alone HT	Menzies N, Abang B, Wanyenze R, Nuwaha F, Mugisha B, Coutinho A, Bunnell R, Mermin J,	2009	The costs and effectiveness of four HIV counseling and testing strategies in	article	AFRO		X						
##	Stand-alone HT	Champenois, K., Le Gall, J.M., Jacquemin, C., Jean, S., Martin, C., Rios, L., Benoit, O., Vermoesen, S., Lert, F., Spire, B. and Yazdanpanah,	2012	ANRS–COM'TEST: description of a community-based HIV testing intervention in non-medical settings for men	article	EURO	MSM	X	X		X			

Under

supplemental table showing all included studies WITH PID

##	Stand-alone HT	Lorente, N., Preau, M., Vernay-Vaisse, C., Mora, M., Blanche, J., Otis, J., Passeron, A., Le Gall, J.M., Dhotte, P., Carrieri, M.P. and Suzan-Monti, M.	2013	Expanding Access to Non-Medicalized Community-Based Rapid Testing to Men Who Have Sex with Men: An Urgent HIV Prevention Intervention (The ANRS-DRAG Study)	article	EURO	MSM	X						
##	Stand-alone HT	Meulbroek, M., Ditzel, E., Saz, J., Taboada, H., Pérez, F., Pérez, A., Carrillo, A., Font, G., Marazzi, G., Uya, J. and Cabrero, J.	2013	BCN Checkpoint, a community-based centre for men who have sex with men in Barcelona, Catalonia, Spain, shows high efficiency in HIV detection and linkage to care: Efficient HIV detection at	article	EURO	MSM	X						
##	Stand-alone HT	Knight, V., Gale, M., Guy, R., Parkhill, N., Holden, J., Leeman, C., McNulty, A., Keen, P. and Wand, H.	2014	A novel time-limited pop-up HIV testing service for gay men in Sydney, Australia, attracts high-risk	article	WPRO	MSM	X						

##	Stand-alone HT	Yan, H., Zhang, M., Zhao, J., Huan, X., Ding, J., Wu, S., Wang, C., Xu, Y., Liu, L., Xu, F. and Yang, H.	2014	The increased effectiveness of HIV preventive intervention among men who have sex with men and of follow-up care for people living with HIV after 'task-shifting' to community-based organizations: a 'cash on service delivery' model	article	WPRO	MSM	X		X				
##	Stand-alone HT	Ferrer L, Loureiro E, Meulbroek M, Folch C, Perez F, Esteve A, Saz J, Taboada H, Pujol F, Casabona J.	2015	High HIV incidence among men who have sex with men attending a community-based voluntary counselling and testing service in Barcelona, Spain: results from the ITACA	article	EURO	MSM	X						
##	Stand-alone HT	Des Jarlais D, Duong HT, Pham Minh K, Khuat OH, Nham TT, Arasteh K, Feelemyer J, Heckathorn DD, Peries M, Moles JP, Laureillard D, Nagot N; (The Drive Study Team)..	2016	Integrated respondent-driven sampling and peer support for persons who inject drugs in Haiphong, Vietnam: a case study with implications for	article	WPRO	MSM	X						

supplemental table showing all included studies WITH PID

##	Stand-alone HT	Engler K, Rollet K, Lessard D, Thomas R, Lebouche B.	2016	Explaining the Presence of "Heterosexual" Female Clients of a Rapid HIV Testing Site Located in the Gay Village of Montreal,	article	AMERO		X							
##	Stand-alone HT	Lazarus L, Patel S, Shaw A, Leblanc S, Lalonde C, Hladio M, Mandryk K, Horvath C, Petrcich W, Kendall C, Tyndall MW; Proud Community Advisory Committee.	2016	Uptake of Community-Based Peer Administered HIV Point-of-Care Testing: Findings from the PROUD Study	article	AMERO	People who inject/use drugs	X	X						
##	Stand-alone HT	Lessard D.; Lebouche' B.; Engler K.; Thomas R.	2016	An analysis of socio-demographic and behavioural factors among immigrant MSM in Montreal from an HIV-testing site sample	article	AMERO	MSM	X							
##	Stand-alone HT	Reif LK, Rivera V, Louis B, Bertrand R, Peck M, Anglade B, Seo G, Abrams EJ, Pape JW, Fitzgerald DW, McNairy ML.	2016			AMERO		X							

ScholarOne, 375 Greenbrier Drive, Charlottesville, VA, 22901

##	Stand-alone HT	Robert E Booth, Jonathan M Davis, Sergey Dvoryak, John T Brewster, Oksana Lisovska, Steffanie A Strathdee, Carl A Latkin	2016	HIV incidence among people who inject drugs (PWIDs) in Ukraine: results from a clustered randomised trial	article	EURO	People who inject/use drugs	X						
##	Stand-alone HT	Vannakit R.; Jantarapakde J.; Pengnonyang S.; Jitjang S.; Janamnuaysook R.; Pankam T.; Trachunthong D.; Pussadee K.; Reankhomfu R.; Lingjongrat D.; Janyam S.; Nakpor T.; Leenasirimakul P.; Jadwattanakul T.; Noriega S.; Charoenying S.; Sattayapanich T.; Arunmanakul A.; Phanuphak P.; Cassell M.; Phanuphak N.	2016	A cohort study of community-based test and treat for men who have sex with men and transgender women: Preliminary findings from Thailand	abstract	SEARO	MSM & Transgender	X						
##	Stand-alone HT	Pham, M.K., Moles, J.P., Thi, H.D., Thi, T.N., Thi, G.H., Thi, T.T., Hai, V.V., Thi, H.K., Vallo, R., Peries, M. and Arasteh, K.	2017	Low HIV incidence but high HCV incidence among people who inject drugs in Haiphong, Vietnam: results of the ANRS 12299/NIDA P30DA011041	abstract	WPRO	People who inject/use drugs	X						

##	Combination of interventions	Outlaw, A.Y., Naar-King, S., Parsons, J.T., Green-Jones, M., Janisse, H. and Secord, E.	2010	Using Motivational Interviewing in HIV Field Outreach With Young African American Men Who Have Sex With Men: A Randomized	article	AMERO	MSM	X	X					
##	Combination of interventions	Stein, R., Green, K., Bell, K., Toledo, C.A., Uhl, G., Moore, A., Shelley, G.A. and Hardnett, F.P.	2011	Provision of HIV Counseling and Testing Services at Five Community-Based Organizations Among Young Men of Color Who Have Sex with Men	article	AMERO	MSM & Transgender	X						
##	Combination of interventions	Qvist, T., Cowan, S.A., Graugaard, C. and Helleberg, M.	2014	High linkage to care in a community-based rapid HIV testing and counseling project among men who have sex with men in	article	EURO	MSM	X			X	X		
##	Combination of interventions	Zhang, D., Meng, S., Xu, P., Lu, H., Zhuang, M., Wu, G., Liu, Y., Pan, X., Yan, H., Chen, X. and Fan, L.	2014	Experience of offering HIV rapid testing to at-risk patients in community health centers in eight Chinese	article	WPRO	MSM	X						

Under r

##	Combination of interventions	Castro R, Ribeiro-Alves M, CorrÃªa RG, Derrico M, Lemos K, Grangeiro JR, Jesus Bd, Pires D, Veloso	2016	The Men Who Have Sex with Men HIV Care Cascade in Rio de Janeiro, Brazil	article	AMERO	MSM	X						
##	Combination of interventions	Castro R, Ribeiro-Alves M, CorrÃªa RG, Derrico M, Lemos K, Grangeiro JR, Jesus Bd, Pires D, Veloso	2016	The Men Who Have Sex with Men HIV Care Cascade in Rio de Janeiro, Brazil	article	AMERO	MSM & Transgender	X						
##	Combination of interventions	Fernandez-Lopez L, Reyes-Uruena J, Agusti C, Kustec T, Klavs I, Casabona C; COBATEST Network group.	2016	The COBATEST network: a platform to perform monitoring and evaluation of HIV community-based testing practices in Europe and conduct operational	article	EURO		X						
##	Combination of interventions	Hoenigl M, Chaillon A, Morris SR, Little SJ.	2016	HIV Infection Rates and Risk Behavior among Young Men undergoing community-based Testing in San Diego	article	AMERO	MSM	X						
##	Combination of interventions	Hoenigl M, Chaillon A, Morris SR, Little SJ.	2016	HIV Infection Rates and Risk Behavior among Young Men undergoing community-based Testing in San Diego	article	AMERO		X						

##	Combination of interventions	Bitimwine, H., Musiime, F., Ajuna, P., Tumbu, P., Nahirya-Ntege, P., & Kekitiinwa, A.	2017	Maximizing targeted testing to improve HIV yield among children and adolescents in Rwenzori	abstract	AFRO		X						
##	Combination of interventions	Casalini, C., Boyee, D., Ndolichimpa, M., Rutabanzibwa, N., Bandio, R., Mlanga, E., &	2017	Key population risk factors associated with differentiated HIV care in	abstract	AFRO	Mixed populations	X						
##	Combination of interventions	Holliday RC, Zellner T, Francis C, Braithwaite RL, McGregor B, Bonhomme J.	2017	Campus and Community HIV and Addiction Prevention (CCHAP): An HIV Testing and Prevention Model to Reach Young African American Adults	article	AMERO		X						
##	Combination of interventions	Ribas Baltrons, Josep; Fernàndez-López, Laura; Casabona I Barbarà, Jordi; Grupo red COBATEST	2017	[Cobatest network: users' characteristics of community-based voluntary,counselling and	article	EURO	Mixed populations	X						
##	Combination of interventions	Zulliger R, Maulsby C, Solomon L, Baytop C, Orr A, Nasrullah M, Shouse L, DiNenno E, Holtgrave D.	2017	Cost-utility of HIV Testing Programs Among Men Who Have Sex with Men in the	article	AMERO	MSM	X						
##	TB index testing	Velen K, Lewis JJ, Charalambous S, Page-Shipp L, Popane F, Churchyard GJ, Hoffmann CJ.	2016	Household HIV Testing Uptake among Contacts of TB Patients in South Africa	article	AFRO		X	X					

Under re

##	School-based HTS	Ijadunola K, Abiona T, Balogun J, Aderounmu A.	2011	Provider-initiated (Opt-out) HIV testing and counselling in a group of university students in Ile-Ife, Nigeria	article	AFRO		X						
##	School-based HTS	Gill, H., Bulman, J., Wallace, H., Evans, A., & Schoeman, S.	2014	Evaluation of a pilot student LGBT sexual health "pop up" clinic	abstract	EURO	MSM & Transgender	X						
##	School-based HTS	Milligan C, Cuneo CN, Rutstein SE, Hicks C.	2014	"Know Your Status": results from a novel, student-run HIV testing initiative on college campuses	article	AMERO	MSM	X		X				
##	School-based HTS	Milligan C, Cuneo CN, Rutstein SE, Hicks C.	2014	"Know Your Status": results from a novel, student-run HIV testing initiative on college campuses	article	AMERO		X						
##	School-based HTS	Okpo E, Corrigan H, Gillies P.	2015	Blood borne virus (BBV) testing in a university setting in North-East Scotland: a	article	EURO		X						
##	School-based HTS	Khawcharoenporn T, Chunloy K, Apisarnthanarak A.	2016	Uptake of HIV testing and counseling, risk perception and linkage to HIV care among Thai university students	article	SEARO		X						

##	Workplace-based HTS	Van der Borght SF, Schim van der Loeff MF, Clevenbergh P, Kabarega JP, Kamo E, van Cranenburgh K, Rijckborst H, Lange JM, Rinke de Wit TF.	2010	Long-term voluntary counseling and testing (VCT) uptake dynamics in a multicountry HIV workplace program in sub-Saharan Africa	article	AFRO		X						
##	Workplace-based HTS	de Beer I, Chani K, Feeley FG, Rinke de Wit TF, Sweeney-Bindels E, Mulongeni P.	2015	Assessing the costs of mobile voluntary counseling and testing at the work place versus facility based voluntary counseling and testing in	article	AFRO		X						
##	Workplace-based HTS	Knoblauch AM, Divall MJ, Owuor M, Nduna K, Ng'uni H, Musunka G, Pascall A, Utzinger J, Winkler MS.	2017	Experience and lessons from health impact assessment guiding prevention and control of HIV/AIDS in a copper mine project, northwestern Zambia	article	AFRO		X						

review

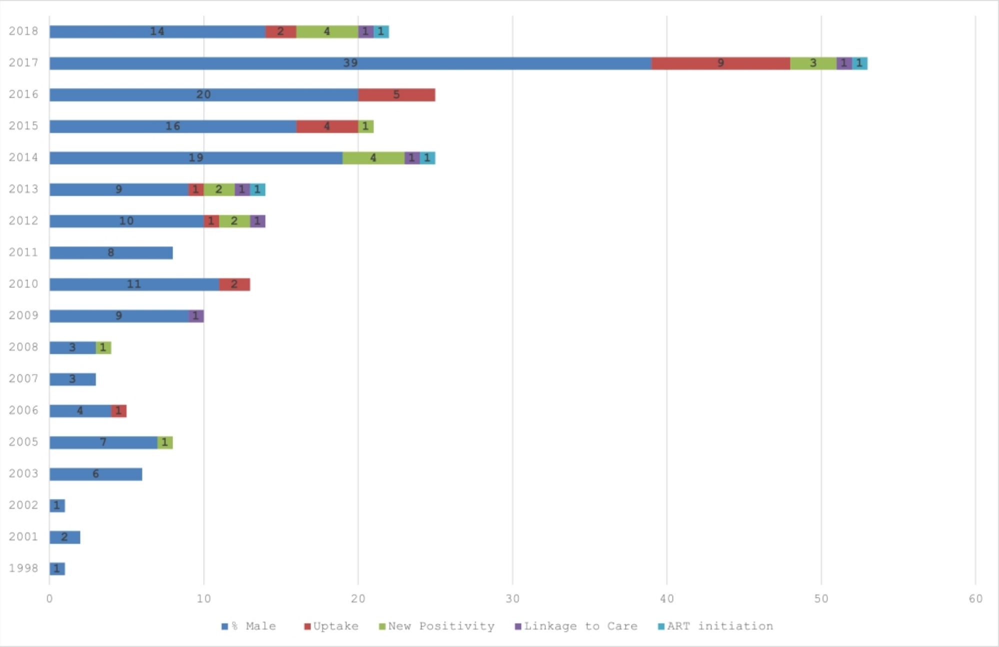

637x411mm (130 x 130 DPI)

Supplemental Digital Content 5a. Quality of studies included in systematic review of CB HTS using Cochrane Collaboration's Risk of Bias Tool							
	Selection bias		Performance and detection bias	Reporting bias	Attrition bias	Other	
**Author, year**	A	B	C	D	E	F	**Total score[1]**
Chamie 2017	U	U	0	U	U	U	0*
Doherty 2013	1	1	U	1	1	0	4
Ezeanolue 2017	1	1	0	1	1	1	5
Fylkesnes 2013	1	1	0	1	1	1	5
Krakowiak 2016A	U	U	0	U	U	U	0*
Lugada 2010	U	U	0	0	1	1	2
Osoti 2013	1	1	0	0	1	1	4
Outlaw 2010	1	U	0	0	1	1	3
Roland 2018	U	U	0	U	U	U	0*
Sibanda 2016	U	U	0	U	U	U	0*
Sibanda 2017	1	0	0	U	1	1	3
Spielberg 2005	U	U	0	U	1	0	1

Quality categories: A: Adequate sequence generation; B: Allocation concealment; C: Blinding of participants, personnel and outcome assessors; D: Incomplete outcome data addressed; E: Free of selection reporting; F: Free of any other bias

[1]Total scores range from 0 to 6. Higher scores indicate low risk of bias; lower scores indicate higher risk of bias.

*Conference abstract only, unable to assess completely

For Peer Review

Supplemental Digital Content 5b. Quality of studies included in systematic review of CB HTS using Newcastle-Ottawa Quality Assessment Scale								
	Selection bias			Confounding	Measurement bias			
**Author, year**	A	B	C	D	E	F	G	**Total score[1]**
Adebajo 2015	U	0	1	1	1	1	U	4
Bailey 2008	U	0	1	1	1	1	U	4
Baisley 2012	1	0	1	2	0	1	1	5
Bogart 2017	0	0	1	2	1	1	1	6
Cawley 2014	U	U	1	0	1	0	U	1
Chamie 2016	U	U	1	1	U	1	U	3
de Beer 2015	U	0	1	0	U	1	U	2
Grabbe 2010	0	1	1	2	1	1	0	6
Hood 2012	1	0	1	0	1	1	1	5
Lipsitz 2014	U	0	1	0	1	1	U	3
Mabuto 2014	0	0	1	0	1	1	1	4
Mark 2017	1	1	1	U	U	1	1	5
Menzies 2009	1	1	1	2	1	1	1	8
Mulogo 2011	U	0	1	0	U	1	1	3
Parker 2015	U	0	1	1	1	1	1	5
Phiri 2016	U	U	1	U	U	1	1	3*
Varela 2016	U	U	NA	U	U	1	1	2*
Wasantioopapokakorn 2018	U	0	1	0	1	1	1	4
Wood 2015	U	0	1	0	U	1	U	2
Zhang 2014	U	U	1	0	1	1	U	3

Under review

A: Representativeness of the cohort in the intervention arm of the average person in the community from which study participants were drawn<br>*B: Representativeness of the cohort in the comparator arm to the intervention arm*<br>*C: Determination of whether or not HTC was used*<br>*D: Outcomes were adjusted for patient-level barriers (e.g., distance to testing site, income level or education level) (up to 2 points)*<br>*E: Assessment of outcome according to gold standard measurement (Cases confirmed through two tests in countries with HIV prevalence >5% and through three tests in countries with HIV prevalence <5%)*<br>*F: Adequate follow-up to detect the outcome (i.e. follow-up visit to determine HIV status)*<br>*G: Attrition (i.e., retention of 70% or more of participants during the study)*
[1]Total scores range from 0 to 8. Points are given for meeting standards of quality related to selection, confounding and measurement. Higher scores indicate low risk of bias; lower scores indicate higher risk of bias.<br>*Conference abstract only, unable to assess completely

Under review

HIV testing uptake (Men)

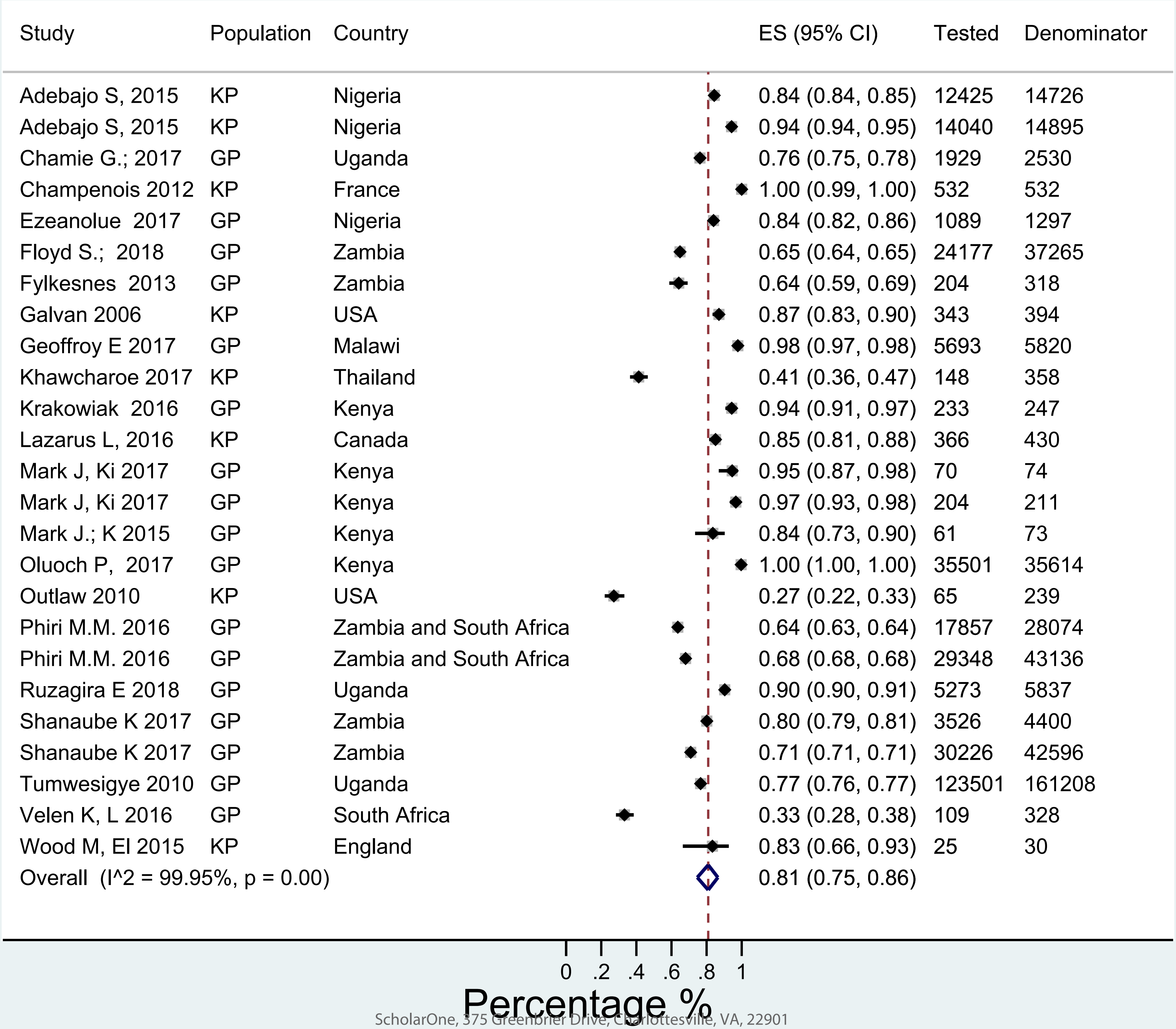

% tested male

Study	Country	ES (95% CI)	Tested	Tested
Adebajo S, 2015	Nigeria	1.00 (1.00, 1.00)	12425	12425
Adebajo S, 2015	Nigeria	1.00 (1.00, 1.00)	14040	14040
Adetunji A 2018	Nigeria	0.53 (0.48, 0.58)	216	408
Arevalo, A 2017	Argentina	0.54 (0.53, 0.56)	2087	3847
Arumainaya 2009	United Kingdom	1.00 (0.98, 1.00)	168	168
Asiimwe S, 2017	Uganda	0.47 (0.47, 0.48)	20729	43696
Bailey, A. 2008	England	1.00 (0.99, 1.00)	280	280
Baisley K, 2012	Tanzania	0.57 (0.56, 0.59)	3091	5399
Baisley K, 2012	Tanzania	0.54 (0.53, 0.56)	2174	4021
Bassett IV 2015	South Africa	0.47 (0.45, 0.49)	1317	2802
Bekolo CE, 2018	Cameroon	0.49 (0.47, 0.51)	994	2024
Bell DAYAM 2003	USA	0.55 (0.52, 0.57)	863	1570
Bell DN, M 2003	USA	0.67 (0.65, 0.69)	1780	2654
Bell SafeS 2003	USA	0.57 (0.39, 0.73)	16	28
Bell TOP-U 2003	USA	0.39 (0.33, 0.45)	104	267
Belza MJ, 2015	Spain	0.48 (0.47, 0.49)	2832	5919
Belza MJ, 2015	Spain	1.00 (1.00, 1.00)	3004	3004
Bingham 2008	USA	1.00 (0.99, 1.00)	458	458
Bitimwine, 2017	Uganda	0.48 (0.46, 0.49)	1956	4091
Bogart LM, 2017	Uganda	0.54 (0.50, 0.57)	443	822
Bradshaw 2005	Australia	0.64 (0.58, 0.69)	197	309
Brady M, H 2011	England	0.61 (0.56, 0.65)	280	459
Brunie A, 2017	Uganda	0.29 (0.24, 0.34)	94	327
Bucher 2007	USA	0.76 (0.73, 0.78)	919	1213
Bucher JB, 2007	USA	0.76 (0.73, 0.78)	919	1213
Casalini, 2017	Tanzania	0.10 (0.10, 0.11)	3995	39180
Castel AD, 2017	United States	0.60 (0.57, 0.62)	876	1471
Castro R, 2016	Brazil	1.00 (0.99, 1.00)	756	756
Castro R, 2016	Brazil	0.95 (0.94, 0.97)	756	793
Cawley C, 2014	Tanzania	0.47 (0.43, 0.50)	400	856
Chamie G, 2016	Uganda & Kenya	0.41 (0.40, 0.41)	42622	104635
Chamie G, 2016	Uganda & Kenya	0.55 (0.55, 0.56)	14771	26672
Chamie G.; 2017	Uganda	1.00 (1.00, 1.00)	1929	1929
Champenois 2012	France	1.00 (0.99, 1.00)	532	532
Chang LW, 2016	Uganda	0.46 (0.45, 0.47)	7904	17119
Chirawu P, 2010	Zimbabwe	0.38 (0.35, 0.40)	513	1368
Dalal W, F 2013	Kenya	0.45 (0.44, 0.45)	8889	19966
Daniels J, 2017	South Africa	0.44 (0.43, 0.44)	31499	72220
Darin KM, 2015	United States	0.41 (0.30, 0.52)	28	69
Daskalakis 2009	USA	1.00 (0.99, 1.00)	493	493
Des Jarlai 2016	Vietnam	1.00 (0.93, 1.00)	49	49
DiCarlo A, 2017	Lesotho	0.34 (0.30, 0.38)	186	553
DiFranceis 1998	USA	0.56 (0.56, 0.57)	6868	12171
Doherty T, 2013	South Africa	0.16 (0.15, 0.18)	229	1392
Engler K, 2016	Canada	0.68 (0.57, 0.78)	52	76
Ezeanolue 2017	Nigeria	1.00 (1.00, 1.00)	1089	1089
Fernandez- 2014	Spain	0.47 (0.45, 0.48)	2326	4993
Fernandez- 2014	Spain	1.00 (1.00, 1.00)	2559	2559
FernÃ nd 2016	Europe	0.65 (0.64, 0.66)	6057	9266
FernÃ¡nd 2015	Spain	0.73 (0.72, 0.74)	17628	24151
FernÃ¡nd 2015	Spain	0.75 (0.73, 0.76)	1619	2168
Ferrer L, 2015	Spain	1.00 (1.00, 1.00)	3544	3544
Floyd S.; 2017	Zambia	0.41 (0.41, 0.41)	34538	84186
Floyd S.; 2018	Zambia	0.42 (0.41, 0.42)	24177	58073
Fylkesnes 2013	Zambia	0.45 (0.41, 0.50)	204	452
Galvan 2006	USA	1.00 (0.99, 1.00)	343	343
Geoffroy E 2017	Malawi	0.41 (0.40, 0.42)	5693	13783
Geoffroy E 2018	Malawi	1.00 (1.00, 1.00)	4378	4378
Gill 2014	United Kingdom	0.40 (0.20, 0.64)	6	15
Govindasam 2011	South Africa	0.40 (0.33, 0.47)	76	192
Grabbe KL, 2010	Kenya	0.58 (0.57, 0.58)	27411	47539
Grabbe KL, 2010	Kenya	0.59 (0.59, 0.60)	8703	14634
Granich R, 2012	Kenya	0.40 (0.39, 0.42)	2090	5198
Hayes R, F 2017	Zambia	0.46 (0.46, 0.47)	28093	60624
Helleringe 2013	Malawi	0.41 (0.37, 0.45)	241	588
Herce ME, 2018	Malawi and Angola	1.00 (1.00, 1.00)	832	832
Hoenigl M, 2016	United States	1.00 (1.00, 1.00)	8926	8926
Hoenigl M, 2016	United States	1.00 (1.00, 1.00)	2944	2944
Holliday R 2017	United States	0.56 (0.54, 0.58)	1339	2385
Hood JE, M 2012	Botswana	0.48 (0.47, 0.48)	10107	21237
Ifekandu 2014	Nigeria	1.00 (0.99, 1.00)	356	356
Ijadunola 2011	Nigeria	0.57 (0.50, 0.63)	142	251
Justman J, 2017	South Africa	0.51 (0.50, 0.52)	5746	11232
Kahn RH, M 2003	USA	0.09 (0.08, 0.10)	247	2807
Kakalou 2014	Greece	0.74 (0.66, 0.81)	87	117
Kawichai S 2007	Thailand	0.48 (0.43, 0.52)	203	427
Kawichai S 2012	Thailand	0.48 (0.47, 0.48)	8168	17153
Keenan 2001	USA	1.00 (0.74, 1.00)	11	11
Keenan 2001	USA	0.68 (0.65, 0.72)	502	735
Khawcharoe 2016	Thailand	0.21 (0.19, 0.23)	277	1307
Khawcharoe 2017	Thailand	1.00 (0.97, 1.00)	148	148
Kimbrough 2009	USA	0.68 (0.66, 0.69)	2152	3172
Knight, V. 2014	Australia	1.00 (0.98, 1.00)	182	182
Knoblauch 2017	Zambia	0.95 (0.94, 0.96)	5288	5564
Knoblauch 2017	Zambia	0.38 (0.37, 0.39)	4428	11638
Krakowiak 2016	Kenya	1.00 (0.98, 1.00)	233	233
Kranzer K, 2011	South Africa	0.52 (0.49, 0.56)	491	936
Kranzer K, 2011	South Africa	0.56 (0.52, 0.59)	488	877
Krisintu, 2017	Thailand	1.00 (0.98, 1.00)	224	224
Lahuerta 2010	Guatemala	0.44 (0.41, 0.47)	362	823
Lazarus L, 2016	Canada	0.80 (0.76, 0.83)	366	458
Lessard D. 2016	Canada	1.00 (1.00, 1.00)	1353	1353
Liang 2005	USA	0.61 (0.56, 0.65)	268	439
Liang TS, 2005	USA	0.63 (0.57, 0.68)	179	284
Liang TS, 2005	USA	0.59 (0.51, 0.66)	92	156
Liebman J, 2002	USA	0.57 (0.51, 0.63)	141	247
Lipsitz, M 2014	Peru	0.87 (0.85, 0.88)	1387	1602
Lister 2005	Australia	1.00 (0.96, 1.00)	102	102
Lorente 2013	France	1.00 (0.98, 1.00)	211	211
Lugada E, 2010	Uganda	0.42 (0.40, 0.44)	1125	2678
Lugada E, 2010	Kenya	0.38 (0.38, 0.39)	18101	47173
Mabuto T, 2014	South Africa	0.46 (0.46, 0.46)	32552	70824
Mabuto T, 2014	South Africa	0.43 (0.42, 0.43)	12372	28937
Mahachi, N 2017	Zimbabwe	0.50 (0.50, 0.51)	13057	25930
Maheswaran 2012	South Africa	0.31 (0.29, 0.33)	490	1585
Maheswaran 2012	South Africa	0.41 (0.38, 0.44)	412	1013
Marcus U, 2015	Germany	1.00 (1.00, 1.00)	1413	1413
Mark J, Ki 2017	Kenya	1.00 (0.95, 1.00)	70	70
Mark J, Ki 2017	Kenya	1.00 (0.98, 1.00)	204	204
Mark J.; K 2015	Kenya	1.00 (0.94, 1.00)	61	61
McCoy SI, 2013	USA	0.64 (0.58, 0.69)	186	291
Mdodo 2014	USA	1.00 (1.00, 1.00)	1072	1072
Menzies DO 2009	Uganda	0.49 (0.49, 0.50)	24283	49470
Menzies N, 2009	Uganda	0.48 (0.46, 0.50)	968	2011
Menzies ST 2009	Uganda	0.51 (0.50, 0.52)	4923	9576
Meulbroek 2013	Spain	1.00 (1.00, 1.00)	14453	14453
Miligan 2014	USA	1.00 (0.97, 1.00)	109	109
Milligan C 2014	USA	0.45 (0.43, 0.48)	635	1408
Morin SF, 2006	Zimbabwe	0.58 (0.55, 0.61)	641	1099
Muchedzi A 2018	Zambabwe	0.53 (0.52, 0.54)	11754	22147
Mulogo EM, 2011	Uganda	0.32 (0.28, 0.36)	156	494
Mutale W, 2010	Zambia	0.42 (0.41, 0.44)	1616	3814
Mutch AJ, 2017	Australia	0.95 (0.94, 0.96)	1144	1199
Negin J, W 2009	Kenya	0.28 (0.27, 0.30)	847	2989
Nglazi MD, 2012	South Africa	1.00 (1.00, 1.00)	4112	4112
Nglazi MD, 2012	South Africa	1.00 (1.00, 1.00)	5214	5214
Ngunu-Gitu 2017	Kenya	0.19 (0.18, 0.20)	1255	6602
O'Laughlin 2018	Uganda	0.44 (0.39, 0.49)	166	378
Obare F, F 2009	Malawi	0.44 (0.43, 0.46)	1326	2987
Ogirima, F 2017	Nigeria	0.48 (0.48, 0.48)	185056	385534
Okiria AG, 2014	Uganda	0.35 (0.35, 0.36)	6994	19801
Okoko, N.A 2017	Kenya	0.47 (0.46, 0.48)	6855	14603
Okpo E, Co 2015	United Kingdom	0.46 (0.39, 0.53)	88	191
Olawore O. 2018	Uganda	0.45 (0.44, 0.46)	6861	15187
Oluoch P, 2017	Kenya	0.49 (0.49, 0.50)	35501	71720
Osoti AO, 2013	Kenya	0.89 (0.83, 0.93)	133	150
Ostermann 2011	Tanzania	0.52 (0.49, 0.56)	460	878
Outlaw 2010	USA	1.00 (0.94, 1.00)	65	65
Parker LA, 2015	eSwatini	0.44 (0.42, 0.46)	895	2043
Parker LA, 2015	eSwatini	0.44 (0.43, 0.45)	3106	7026
Pham M. 2017	Vietnam	0.90 (0.87, 0.92)	543	603
Phiri M.M. 2016	Zambia and South Africa	1.00 (1.00, 1.00)	17857	17857
Phiri M.M. 2016	Zambia and South Africa	1.00 (1.00, 1.00)	29348	29348
Qvist, T. 2014	Denmark	1.00 (1.00, 1.00)	3012	3012
Reif LK, R 2016	Haiti	0.24 (0.22, 0.25)	797	3348
Ribas Balt 2017	Spain	0.67 (0.66, 0.68)	3602	5385
Robert E B 2016	Ukraine	0.75 (0.72, 0.77)	899	1200
Roland M.; 2018	Botswana	0.42 (0.42, 0.43)	8181	19349
Roland M.; 2018	Botswana	0.56 (0.55, 0.56)	16930	30344
Rose 2006	USA	0.90 (0.71, 0.97)	19	21
Ruzagira E 2018	Uganda	0.44 (0.43, 0.44)	5273	12100
SEARCH Col 2017	Uganda	0.41 (0.39, 0.43)	865	2119
Shanaube K 2017	Zambia	0.40 (0.39, 0.41)	3147	7790
Shanaube K 2017	Zambia	0.40 (0.39, 0.42)	3526	8707
Shanaube K 2017	Zambia	0.45 (0.45, 0.46)	30226	66894
Sibanda EL 2017	Zimbabwe	0.45 (0.44, 0.46)	6377	14099
Sibanda EL 2017	Zimbabwe	0.46 (0.45, 0.47)	4892	10580
Sinha, P.; 2018	South Africa	0.27 (0.26, 0.28)	1334	4985
Sinha, P.; 2018	South Africa	0.20 (0.19, 0.22)	554	2723
Smyrnov, P 2017	Ukraine	0.69 (0.69, 0.70)	9669	13936
Spielberg, 2005	USA	0.71 (0.66, 0.76)	230	324
Spielberg, 2005	USA	1.00 (0.99, 1.00)	437	437
Stein 2011	USA	1.00 (1.00, 1.00)	1723	1723
Suggaravet 2003	Thailand	0.61 (0.58, 0.64)	546	890
Tumwesigye 2010	Uganda	0.47 (0.46, 0.47)	123501	264966
Van der Bo 2010	DRC, Rwanda, Burundi, Congo & Nigeria	0.59 (0.58, 0.60)	5744	9723
Vannakit R 2016	Thailand	0.71 (0.68, 0.74)	731	1029
Velen K, L 2016	South Africa	0.36 (0.31, 0.41)	109	304
Vreeman RC 2010	Kenya	0.51 (0.48, 0.53)	657	1294
Wasantioop 2018	Thailand	0.84 (0.82, 0.85)	1606	1923
Were WA, M 2006	Uganda	0.48 (0.46, 0.50)	1130	2348
Wood M, El 2015	England	1.00 (0.87, 1.00)	25	25
Wringe A, 2008	Tanzania	0.55 (0.52, 0.58)	605	1100
Yan, H. et 2014	China	1.00 (1.00, 1.00)	17091	17091
Zhang 2014	China	1.00 (1.00, 1.00)	31406	31406
Zhang D, M 2014	China	0.25 (0.24, 0.26)	5896	23609
Zulliger R 2017	USA	1.00 (1.00, 1.00)	27475	27475
de Beer I, 2015	Namibia	0.72 (0.70, 0.73)	3674	5124
de la Fuen 2009	Spain	0.61 (0.59, 0.62)	4325	7138
van Nieker 2017	South Africa	0.50 (0.50, 0.51)	34688	68877
van Rooyen 2012	South Africa	0.56 (0.53, 0.59)	554	988
van Rooyen 2013	South Africa	0.33 (0.30, 0.37)	222	671
Overall (I^2 = 99.97%, p = 0.00)		0.70 (0.67, 0.74)		

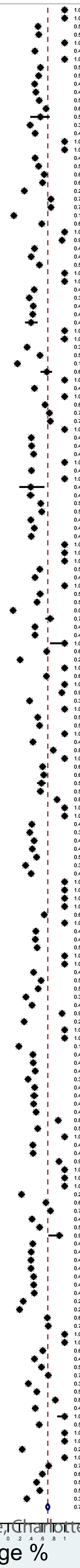

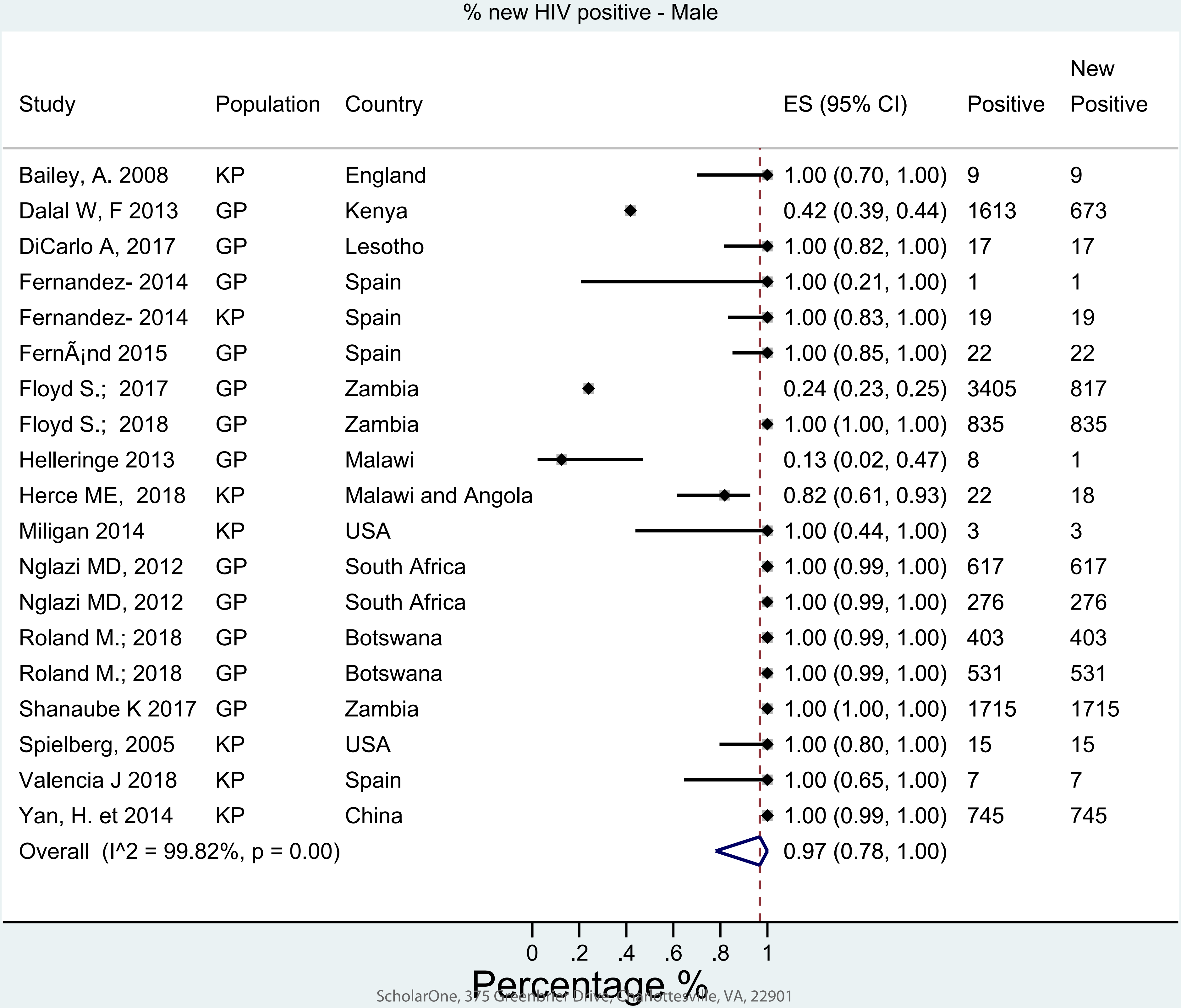

ScholarOne, 375 Greenbrier Drive, Charlottesville, VA, 22901

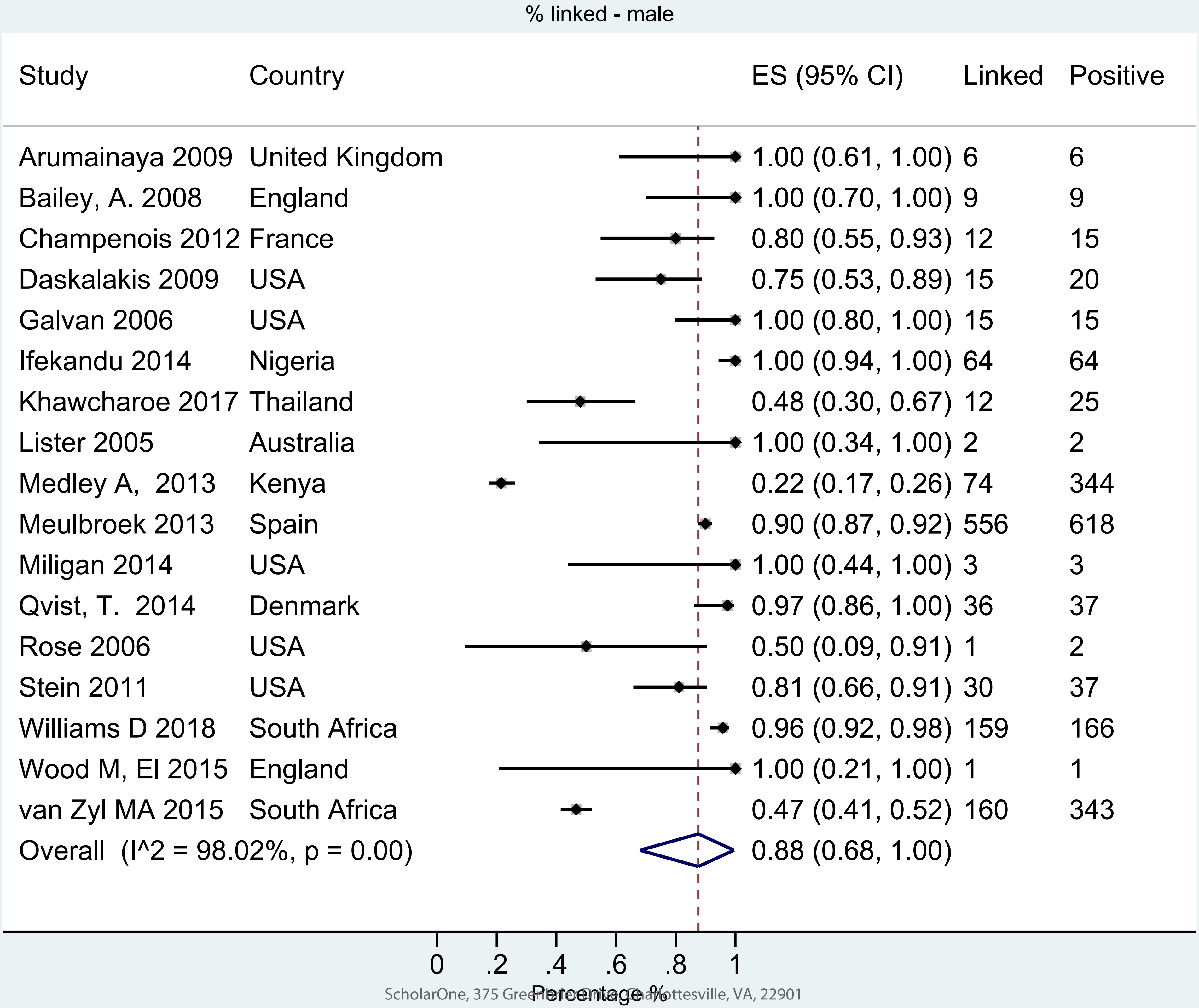
% linked - male
Study
Country
ES (95% CI)
Linked
Positive
Arumainaya 2009 United Kingdom 1.00 (0.61, 1.00) 6 6
Bailey, A. 2008 England 1.00 (0.70, 1.00) 9 9
Champenois 2012 France 0.80 (0.55, 0.93) 12 15
Daskalakis 2009 USA 0.75 (0.53, 0.89) 15 20
Galvan 2006 USA 1.00 (0.80, 1.00) 15 15
Ifekandu 2014 Nigeria 1.00 (0.94, 1.00) 64 64
Khawcharoe 2017 Thailand 0.48 (0.30, 0.67) 12 25
Lister 2005 Australia 1.00 (0.34, 1.00) 2 2
Medley A, 2013 Kenya 0.22 (0.17, 0.26) 74 344
Meulbroek 2013 Spain 0.90 (0.87, 0.92) 556 618
Miligan 2014 USA 1.00 (0.44, 1.00) 3 3
Qvist, T. 2014 Denmark 0.97 (0.86, 1.00) 36 37
Rose 2006 USA 0.50 (0.09, 0.91) 1 2
Stein 2011 USA 0.81 (0.66, 0.91) 30 37
Williams D 2018 South Africa 0.96 (0.92, 0.98) 159 166
Wood M, El 2015 England 1.00 (0.21, 1.00) 1 1
van Zyl MA 2015 South Africa 0.47 (0.41, 0.52) 160 343
Overall (I^2 = 98.02%, p = 0.00) 0.88 (0.68, 1.00)
0
.2
.4
.6
.8
1
Percentage %

Initiated ART (%)

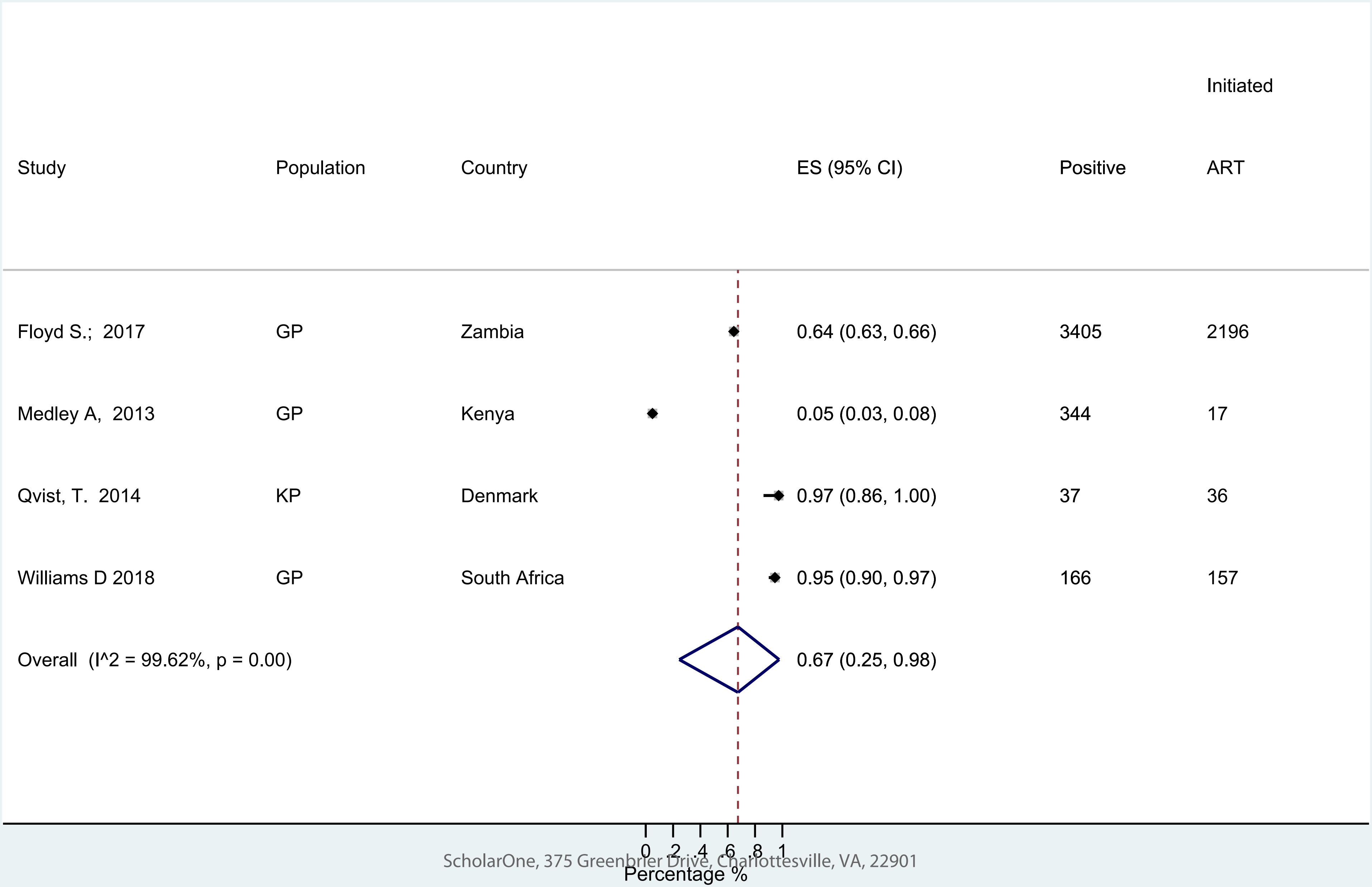

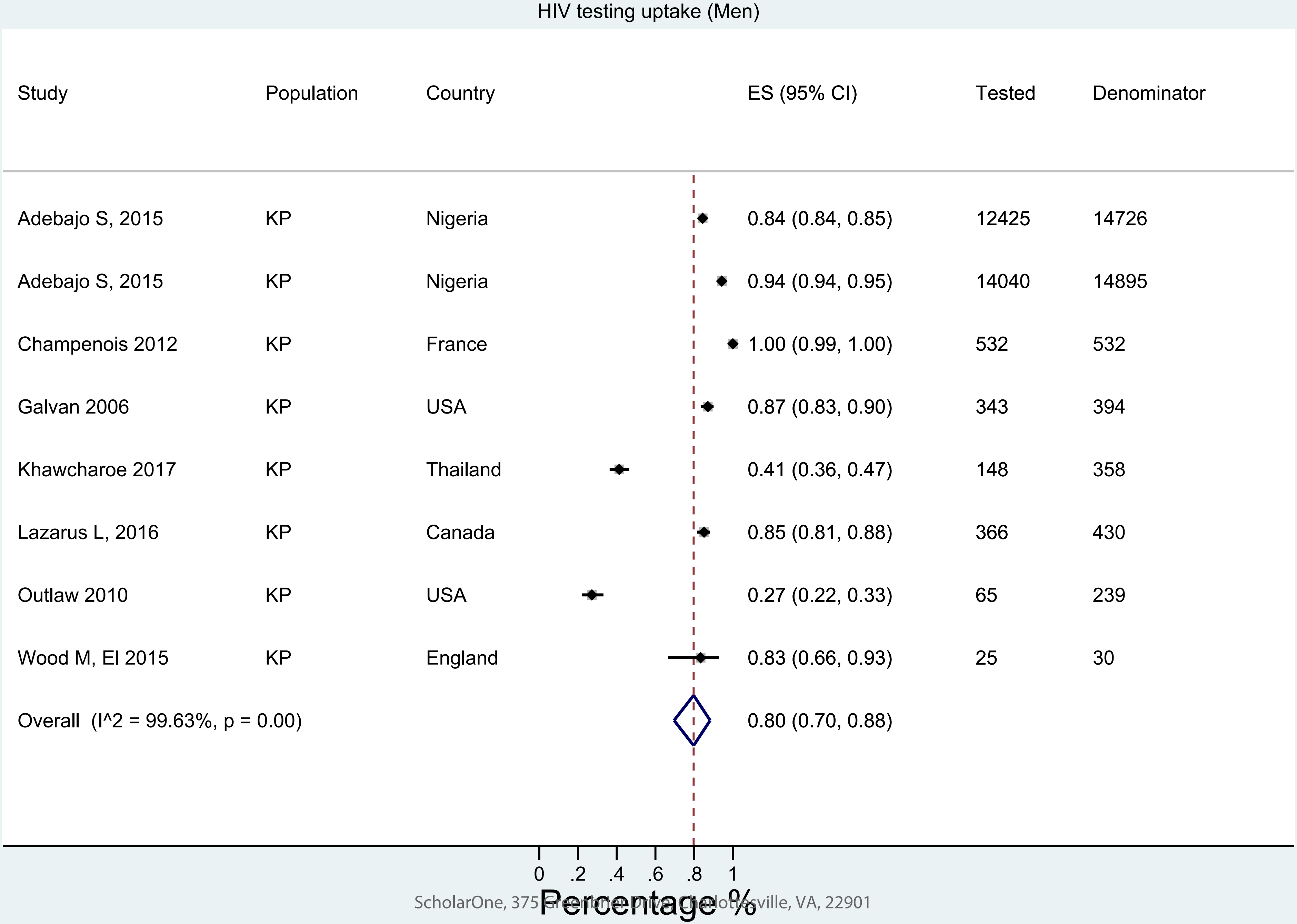
HIV testing uptake (Men)
Study
Population
Country
ES (95% CI)
Tested
Denominator
Adebajo S, 2015 KP Nigeria 0.84 (0.84, 0.85) 12425 14726
Adebajo S, 2015 KP Nigeria 0.94 (0.94, 0.95) 14040 14895
Champenois 2012 KP France 1.00 (0.99, 1.00) 532 532
Galvan 2006 KP USA 0.87 (0.83, 0.90) 343 394
Khawcharoe 2017 KP Thailand 0.41 (0.36, 0.47) 148 358
Lazarus L, 2016 KP Canada 0.85 (0.81, 0.88) 366 430
Outlaw 2010 KP USA 0.27 (0.22, 0.33) 65 239
Wood M, El 2015 KP England 0.83 (0.66, 0.93) 25 30
Overall (I^2 = 99.63%, p = 0.00) 0.80 (0.70, 0.88)
0
.2
.4
.6
.8
1
Percentage %

% tested male

Study	Country	ES (95% CI)	Tested	Tested
Adebajo S, 2015	Nigeria	1.00 (1.00, 1.00)	12425	12425
Adebajo S, 2015	Nigeria	1.00 (1.00, 1.00)	14040	14040
Arumainaya 2009	United Kingdom	1.00 (0.98, 1.00)	168	168
Bailey, A. 2008	England	1.00 (0.99, 1.00)	280	280
Belza MJ, 2015	Spain	1.00 (1.00, 1.00)	3004	3004
Bingham 2008	USA	1.00 (0.99, 1.00)	458	458
Bradshaw 2005	Australia	0.64 (0.58, 0.69)	197	309
Bucher 2007	USA	0.76 (0.73, 0.78)	919	1213
Casalini, 2017	Tanzania	0.10 (0.10, 0.11)	3995	39180
Castro R, 2016	Brazil	1.00 (0.99, 1.00)	756	756
Castro R, 2016	Brazil	0.95 (0.94, 0.97)	756	793
Champenois 2012	France	1.00 (0.99, 1.00)	532	532
Daskalakis 2009	USA	1.00 (0.99, 1.00)	493	493
Des Jarlai 2016	Vietnam	1.00 (0.93, 1.00)	49	49
DiFranceis 1998	USA	0.56 (0.56, 0.57)	6868	12171
Fernandez- 2014	Spain	1.00 (1.00, 1.00)	2559	2559
Ferrer L, 2015	Spain	1.00 (1.00, 1.00)	3544	3544
Galvan 2006	USA	1.00 (0.99, 1.00)	343	343
Gill 2014	United Kingdom	0.40 (0.20, 0.64)	6	15
Herce ME, 2018	Malawi and Angola	1.00 (1.00, 1.00)	832	832
Hoenigl M, 2016	United States	1.00 (1.00, 1.00)	8926	8926
Ifekandu 2014	Nigeria	1.00 (0.99, 1.00)	356	356
Kakalou 2014	Greece	0.74 (0.66, 0.81)	87	117
Keenan 2001	USA	1.00 (0.74, 1.00)	11	11
Keenan 2001	USA	0.68 (0.65, 0.72)	502	735
Khawcharoe 2017	Thailand	1.00 (0.97, 1.00)	148	148
Kimbrough 2009	USA	0.68 (0.66, 0.69)	2152	3172
Knight, V. 2014	Australia	1.00 (0.98, 1.00)	182	182
Krisintu, 2017	Thailand	1.00 (0.98, 1.00)	224	224
Lahuerta 2010	Guatemala	0.44 (0.41, 0.47)	362	823
Lazarus L, 2016	Canada	0.80 (0.76, 0.83)	366	458
Lessard D. 2016	Canada	1.00 (1.00, 1.00)	1353	1353
Liang 2005	USA	0.61 (0.56, 0.65)	268	439
Lipsitz, M 2014	Peru	0.87 (0.85, 0.88)	1387	1602
Lister 2005	Australia	1.00 (0.96, 1.00)	102	102
Lorente 2013	France	1.00 (0.98, 1.00)	211	211
Marcus U, 2015	Germany	1.00 (1.00, 1.00)	1413	1413
Mdodo 2014	USA	1.00 (1.00, 1.00)	1072	1072
Meulbroek 2013	Spain	1.00 (1.00, 1.00)	14453	14453
Miligan 2014	USA	1.00 (0.97, 1.00)	109	109
Ngunu-Gitu 2017	Kenya	0.19 (0.18, 0.20)	1255	6602
Outlaw 2010	USA	1.00 (0.94, 1.00)	65	65
Pham M. 2017	Vietnam	0.90 (0.87, 0.92)	543	603
Qvist, T. 2014	Denmark	1.00 (1.00, 1.00)	3012	3012
Ribas Balt 2017	Spain	0.67 (0.66, 0.68)	3602	5385
Robert E B 2016	Ukraine	0.75 (0.72, 0.77)	899	1200
Rose 2006	USA	0.90 (0.71, 0.97)	19	21
Smyrnov, P 2017	Ukraine	0.69 (0.69, 0.70)	9669	13936
Spielberg, 2005	USA	0.71 (0.66, 0.76)	230	324
Spielberg, 2005	USA	1.00 (0.99, 1.00)	437	437
Stein 2011	USA	1.00 (1.00, 1.00)	1723	1723
Vannakit R 2016	Thailand	0.71 (0.68, 0.74)	731	1029
Wasantioop 2018	Thailand	0.84 (0.82, 0.85)	1606	1923
Wood M, El 2015	England	1.00 (0.87, 1.00)	25	25
Yan, H. et 2014	China	1.00 (1.00, 1.00)	17091	17091
Zhang 2014	China	1.00 (1.00, 1.00)	31406	31406
Zulliger R 2017	USA	1.00 (1.00, 1.00)	27475	27475
Overall (I^2 = 99.98%, p = 0.00)		0.94 (0.86, 0.99)		

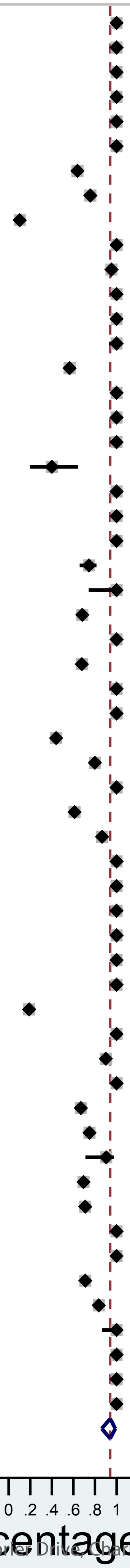

% new HIV positive - Male

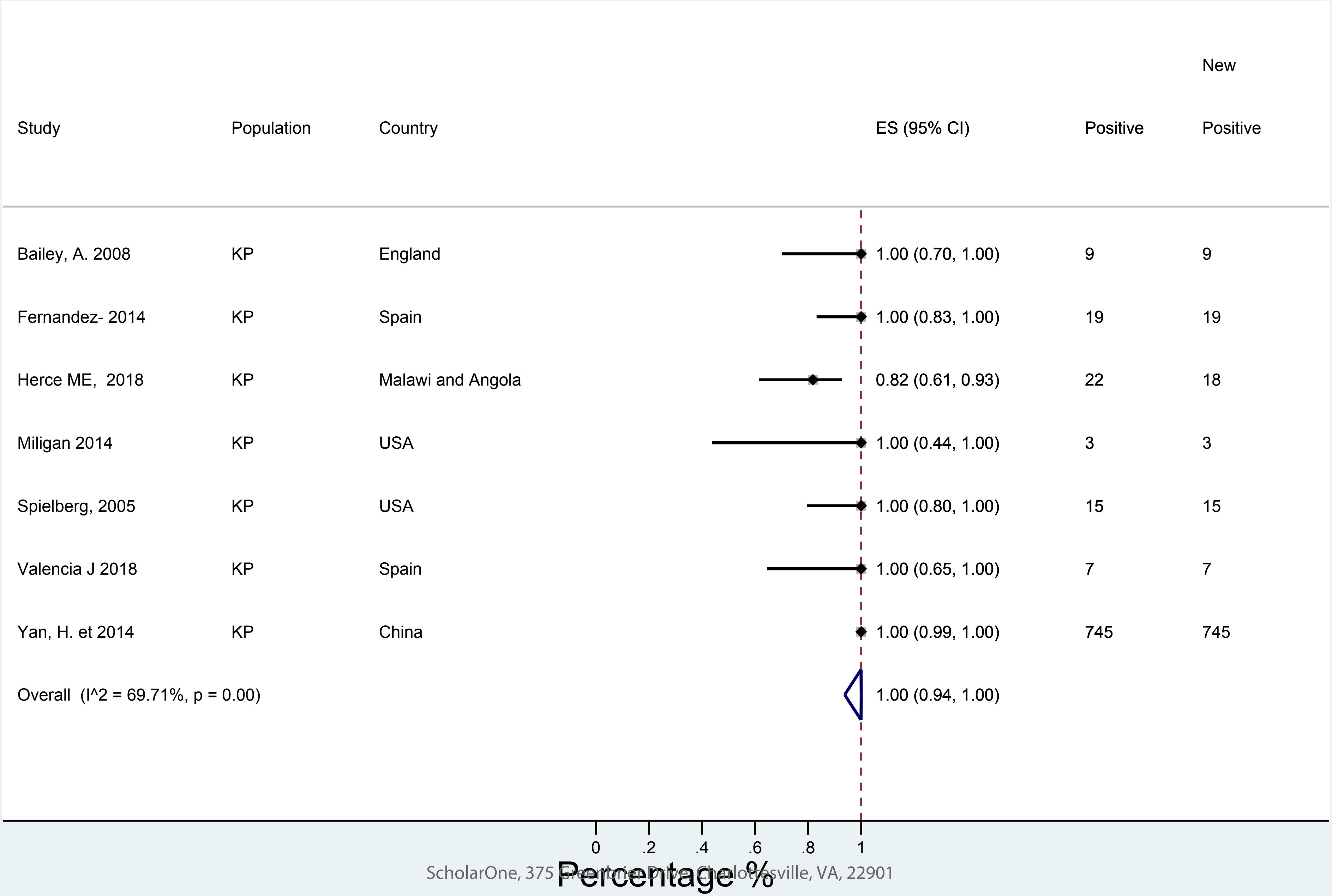

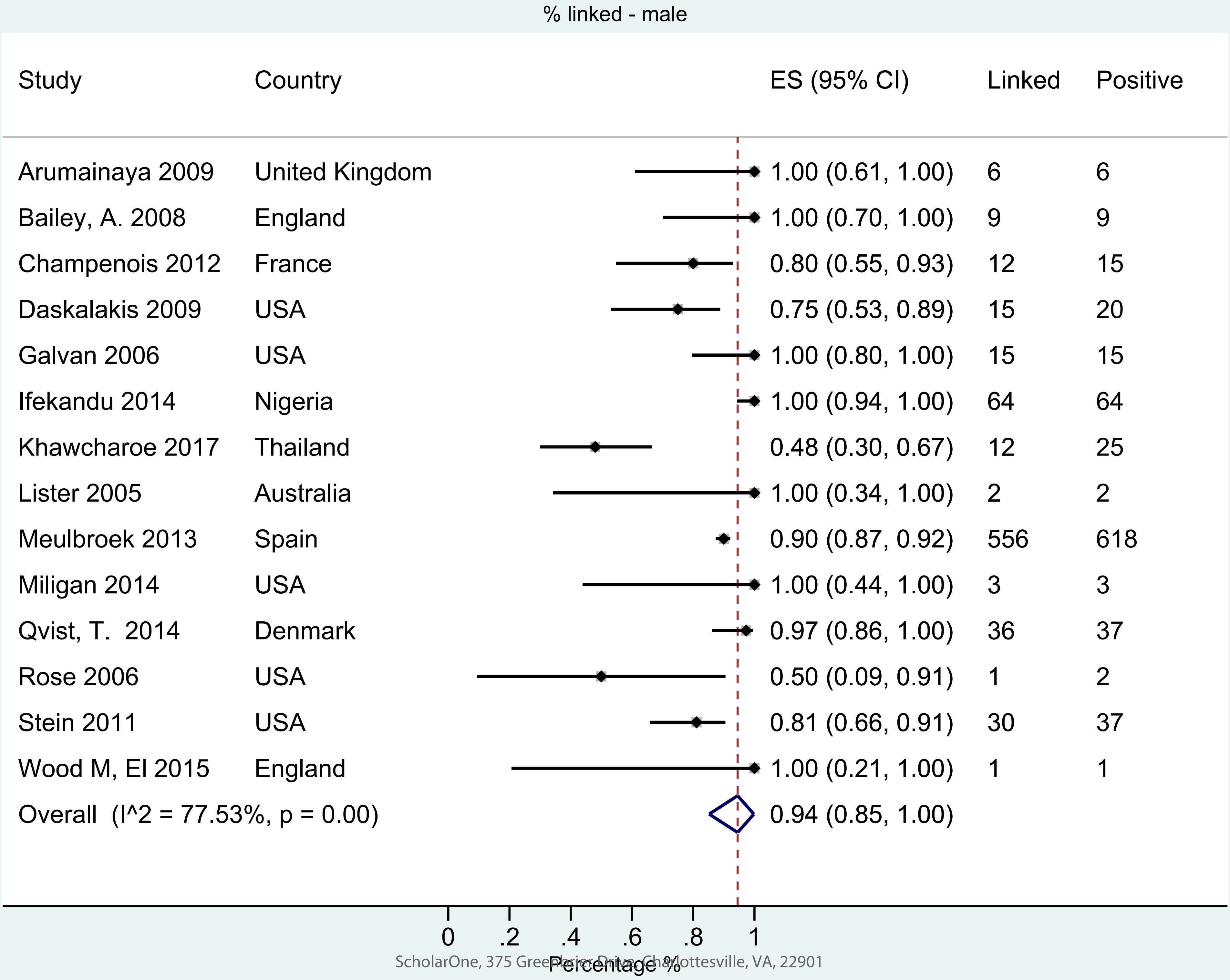
% linked - male
Study
Country
ES (95% CI)
Linked
Positive
Arumainaya 2009
United Kingdom
1.00 (0.61, 1.00)
6
6
Bailey, A. 2008
England
1.00 (0.70, 1.00)
9
9
Champenois 2012
France
0.80 (0.55, 0.93)
12
15
Daskalakis 2009
USA
0.75 (0.53, 0.89)
15
20
Galvan 2006
USA
1.00 (0.80, 1.00)
15
15
Ifekandu 2014
Nigeria
1.00 (0.94, 1.00)
64
64
Khawcharoe 2017
Thailand
0.48 (0.30, 0.67)
12
25
Lister 2005
Australia
1.00 (0.34, 1.00)
2
2
Meulbroek 2013
Spain
0.90 (0.87, 0.92)
556
618
Miligan 2014
USA
1.00 (0.44, 1.00)
3
3
Qvist, T. 2014
Denmark
0.97 (0.86, 1.00)
36
37
Rose 2006
USA
0.50 (0.09, 0.91)
1
2
Stein 2011
USA
0.81 (0.66, 0.91)
30
37
Wood M, El 2015
England
1.00 (0.21, 1.00)
1
1
Overall (I^2 = 77.53%, p = 0.00)
0.94 (0.85, 1.00)
0
.2
.4
.6
.8
1
Percentage %

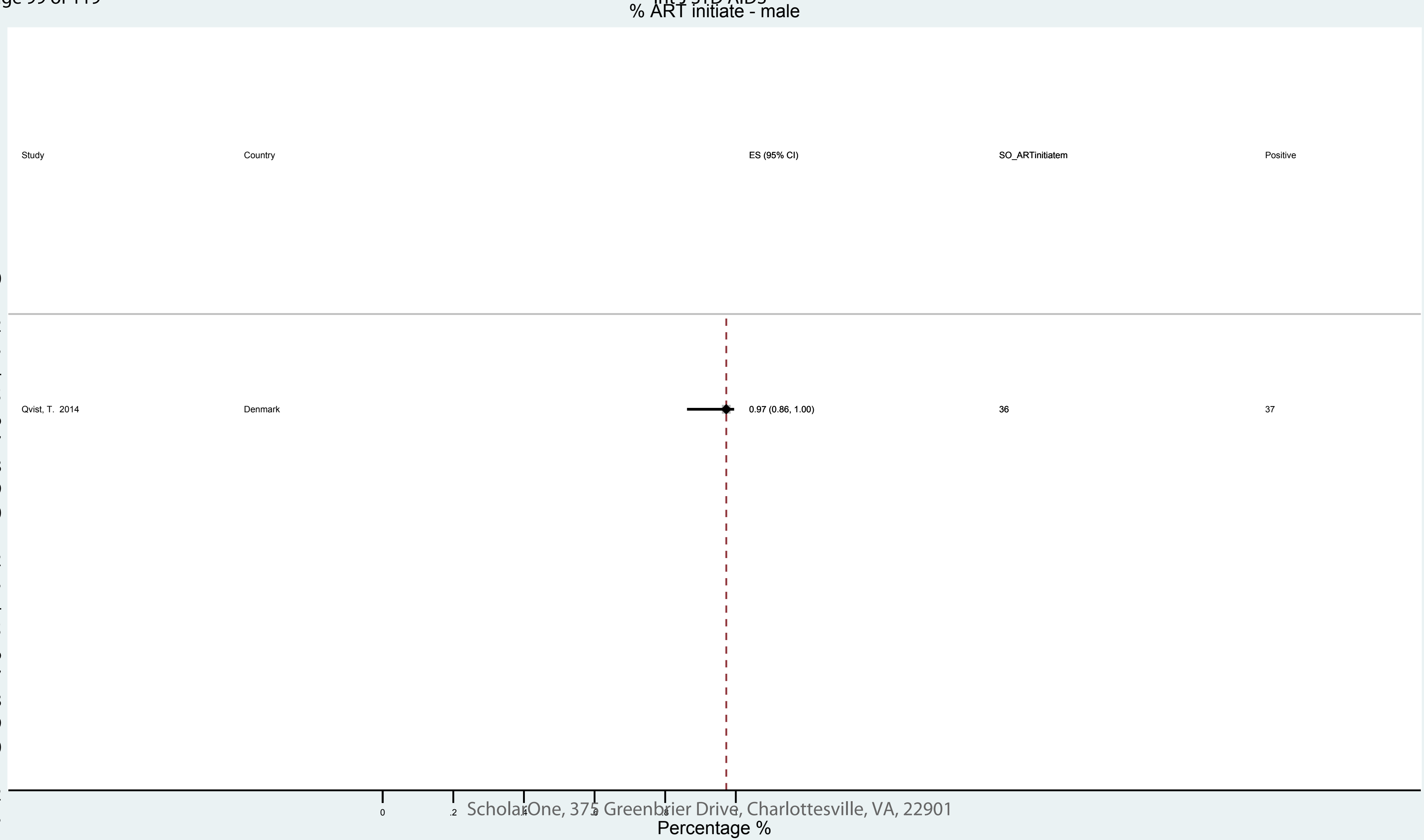
% ART initiate - male
Study
Country
ES (95% CI)
SO_ARTinitiatem
Positive
Qvist, T. 2014
Denmark
0.97 (0.86, 1.00)
36
37
0
.2
Percentage %

HIV testing uptake (Men)

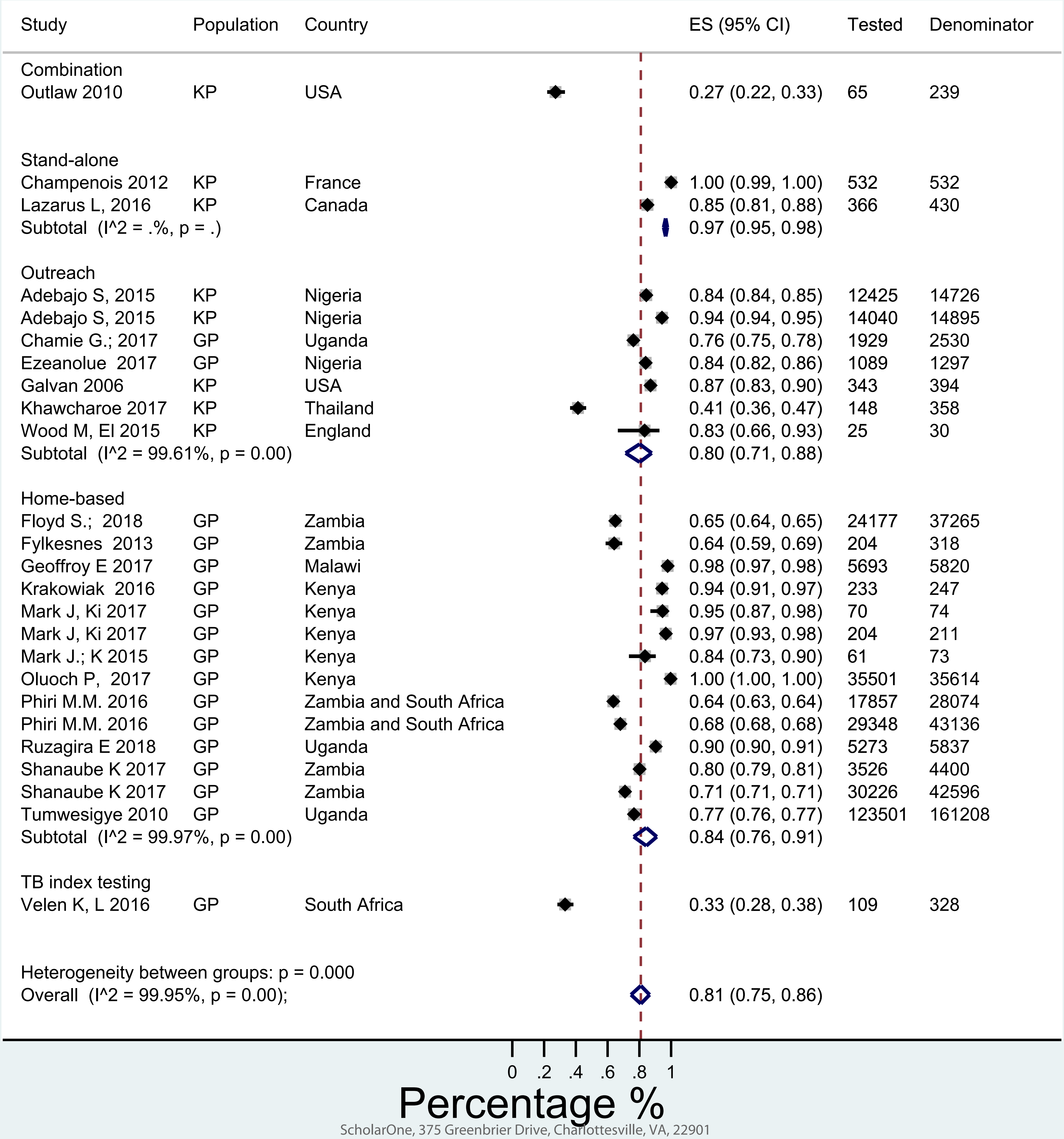

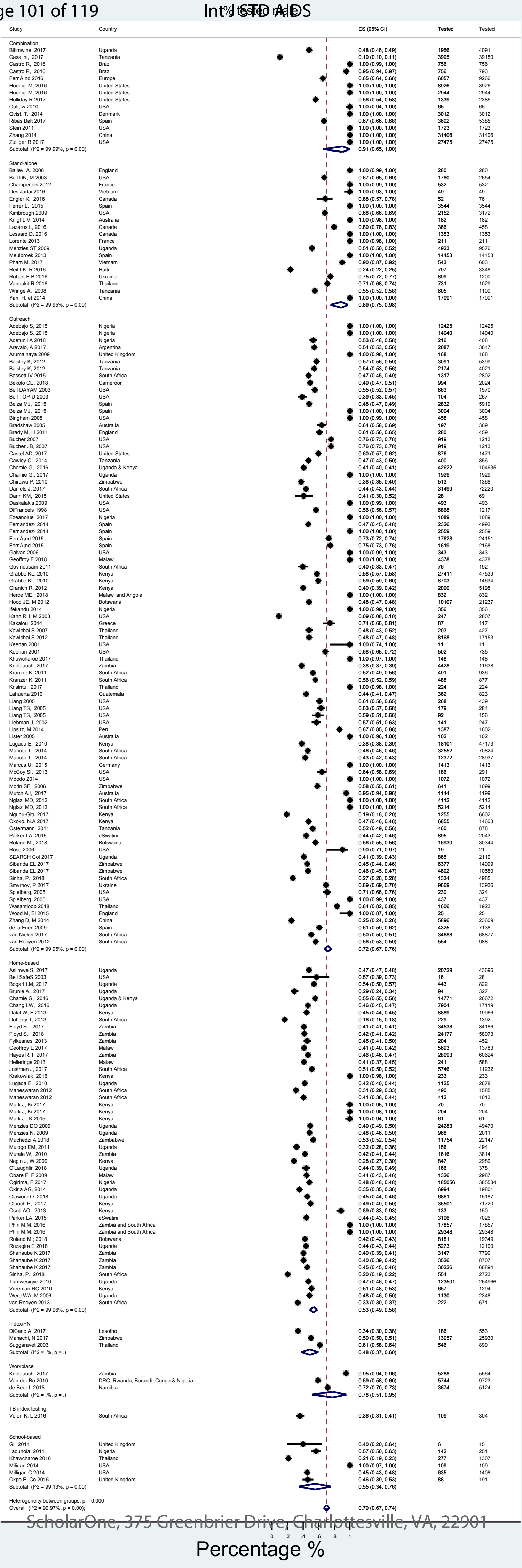

ScholarOne, 375 Greenbrier Drive, Charlottesville, VA, 22901

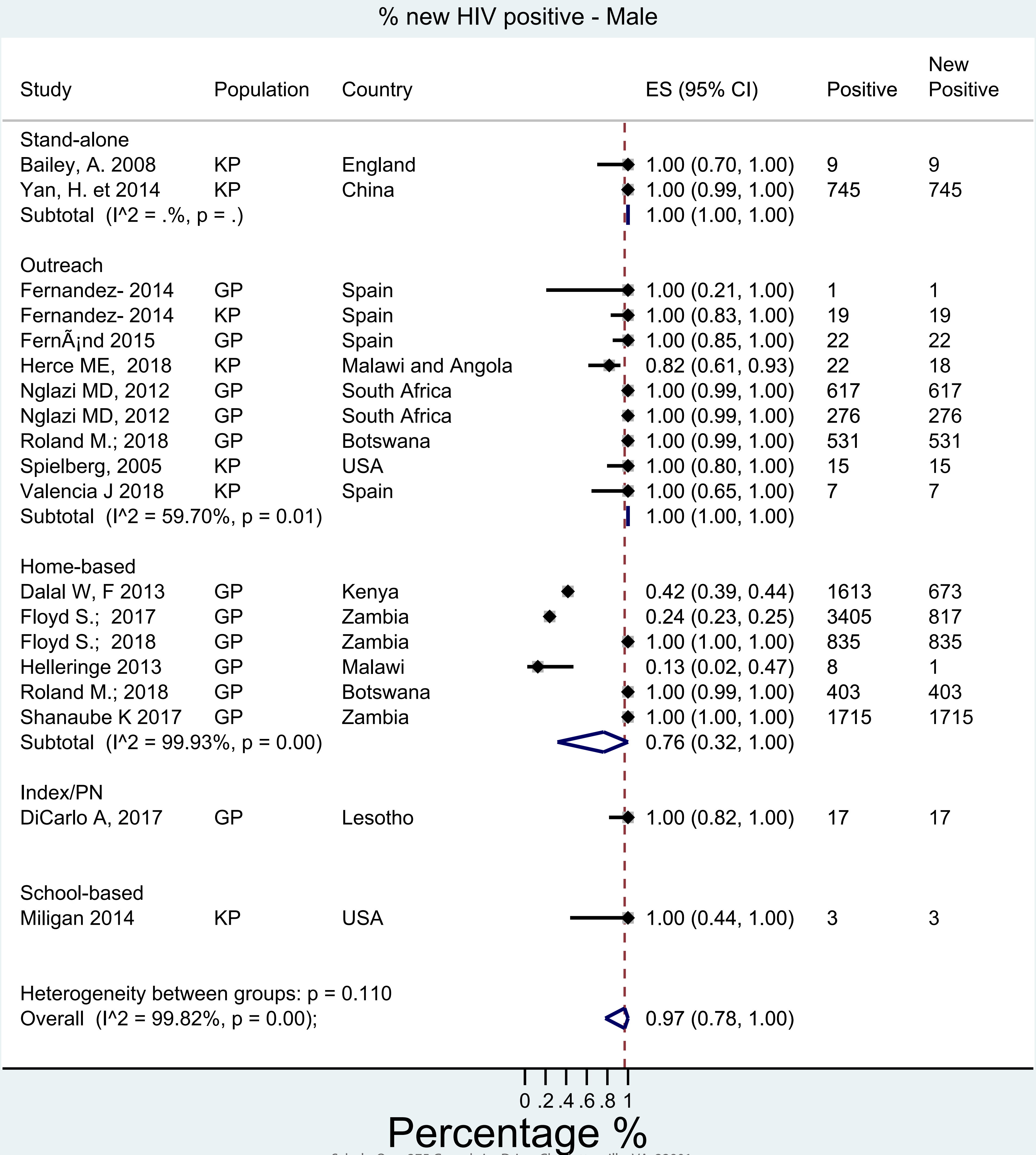
% new HIV positive - Male
Study
Population
Country
ES (95% CI)
Positive
New Positive
Stand-alone
Bailey, A. 2008 KP England 1.00 (0.70, 1.00) 9 9
Yan, H. et 2014 KP China 1.00 (0.99, 1.00) 745 745
Subtotal (I^2 = .%, p = .) 1.00 (1.00, 1.00)
Outreach
Fernandez- 2014 GP Spain 1.00 (0.21, 1.00) 1 1
Fernandez- 2014 KP Spain 1.00 (0.83, 1.00) 19 19
FernÃ¡nd 2015 GP Spain 1.00 (0.85, 1.00) 22 22
Herce ME, 2018 KP Malawi and Angola 0.82 (0.61, 0.93) 22 18
Nglazi MD, 2012 GP South Africa 1.00 (0.99, 1.00) 617 617
Nglazi MD, 2012 GP South Africa 1.00 (0.99, 1.00) 276 276
Roland M.; 2018 GP Botswana 1.00 (0.99, 1.00) 531 531
Spielberg, 2005 KP USA 1.00 (0.80, 1.00) 15 15
Valencia J 2018 KP Spain 1.00 (0.65, 1.00) 7 7
Subtotal (I^2 = 59.70%, p = 0.01) 1.00 (1.00, 1.00)
Home-based
Dalal W, F 2013 GP Kenya 0.42 (0.39, 0.44) 1613 673
Floyd S.; 2017 GP Zambia 0.24 (0.23, 0.25) 3405 817
Floyd S.; 2018 GP Zambia 1.00 (1.00, 1.00) 835 835
Helleringe 2013 GP Malawi 0.13 (0.02, 0.47) 8 1
Roland M.; 2018 GP Botswana 1.00 (0.99, 1.00) 403 403
Shanaube K 2017 GP Zambia 1.00 (1.00, 1.00) 1715 1715
Subtotal (I^2 = 99.93%, p = 0.00) 0.76 (0.32, 1.00)
Index/PN
DiCarlo A, 2017 GP Lesotho 1.00 (0.82, 1.00) 17 17
School-based
Miligan 2014 KP USA 1.00 (0.44, 1.00) 3 3
Heterogeneity between groups: p = 0.110
Overall (I^2 = 99.82%, p = 0.00); 0.97 (0.78, 1.00)
0 .2 .4 .6 .8 1
Percentage %

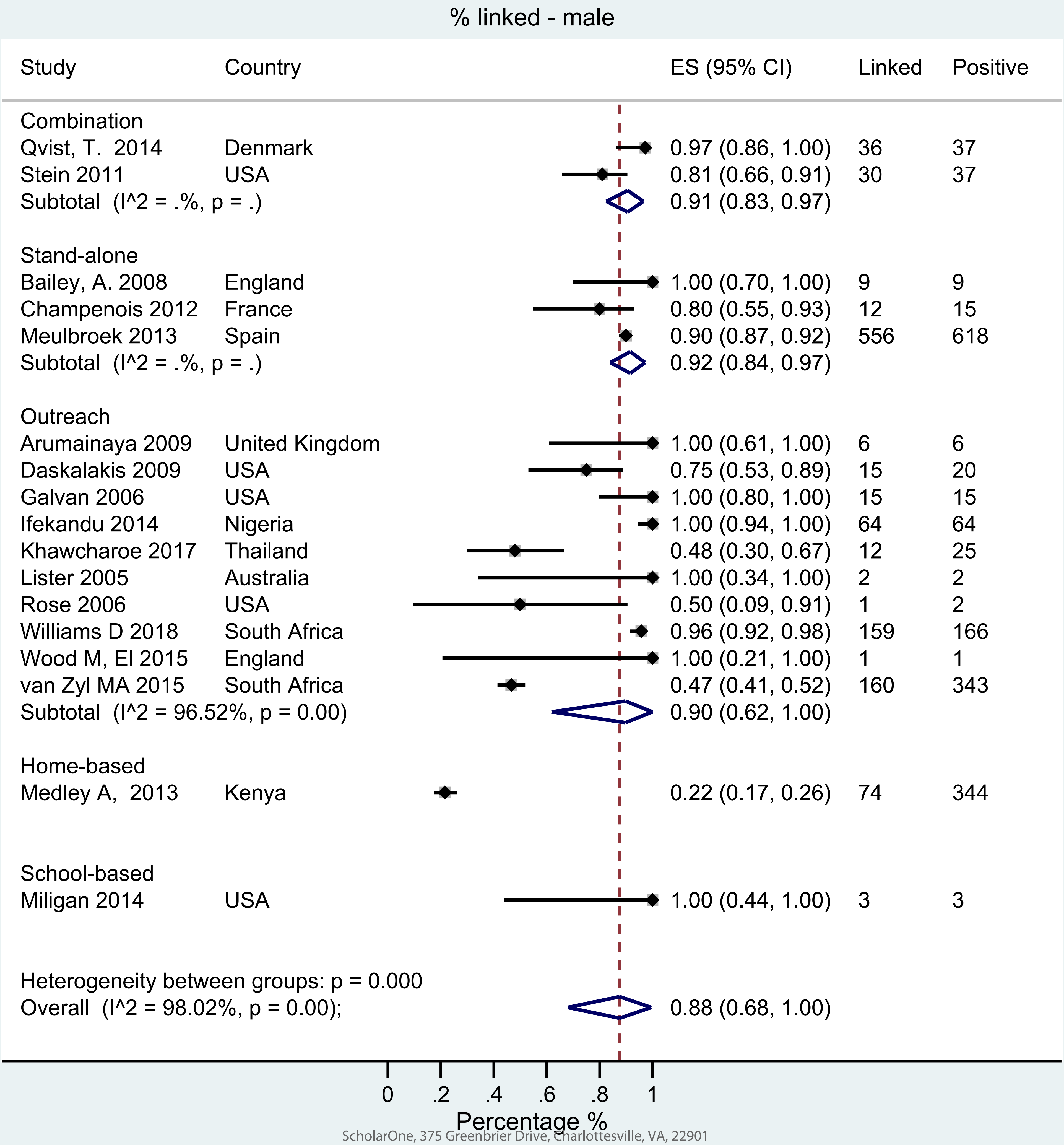
% linked - male
Study
Country
ES (95% CI)
Linked
Positive
Combination
Qvist, T. 2014 Denmark 0.97 (0.86, 1.00) 36 37
Stein 2011 USA 0.81 (0.66, 0.91) 30 37
Subtotal (I^2 = .%, p = .) 0.91 (0.83, 0.97)
Stand-alone
Bailey, A. 2008 England 1.00 (0.70, 1.00) 9 9
Champenois 2012 France 0.80 (0.55, 0.93) 12 15
Meulbroek 2013 Spain 0.90 (0.87, 0.92) 556 618
Subtotal (I^2 = .%, p = .) 0.92 (0.84, 0.97)
Outreach
Arumainaya 2009 United Kingdom 1.00 (0.61, 1.00) 6 6
Daskalakis 2009 USA 0.75 (0.53, 0.89) 15 20
Galvan 2006 USA 1.00 (0.80, 1.00) 15 15
Ifekandu 2014 Nigeria 1.00 (0.94, 1.00) 64 64
Khawcharoe 2017 Thailand 0.48 (0.30, 0.67) 12 25
Lister 2005 Australia 1.00 (0.34, 1.00) 2 2
Rose 2006 USA 0.50 (0.09, 0.91) 1 2
Williams D 2018 South Africa 0.96 (0.92, 0.98) 159 166
Wood M, El 2015 England 1.00 (0.21, 1.00) 1 1
van Zyl MA 2015 South Africa 0.47 (0.41, 0.52) 160 343
Subtotal (I^2 = 96.52%, p = 0.00) 0.90 (0.62, 1.00)
Home-based
Medley A, 2013 Kenya 0.22 (0.17, 0.26) 74 344
School-based
Miligan 2014 USA 1.00 (0.44, 1.00) 3 3
Heterogeneity between groups: p = 0.000
Overall (I^2 = 98.02%, p = 0.00); 0.88 (0.68, 1.00)
0
.2
.4
.6
.8
1
Percentage %

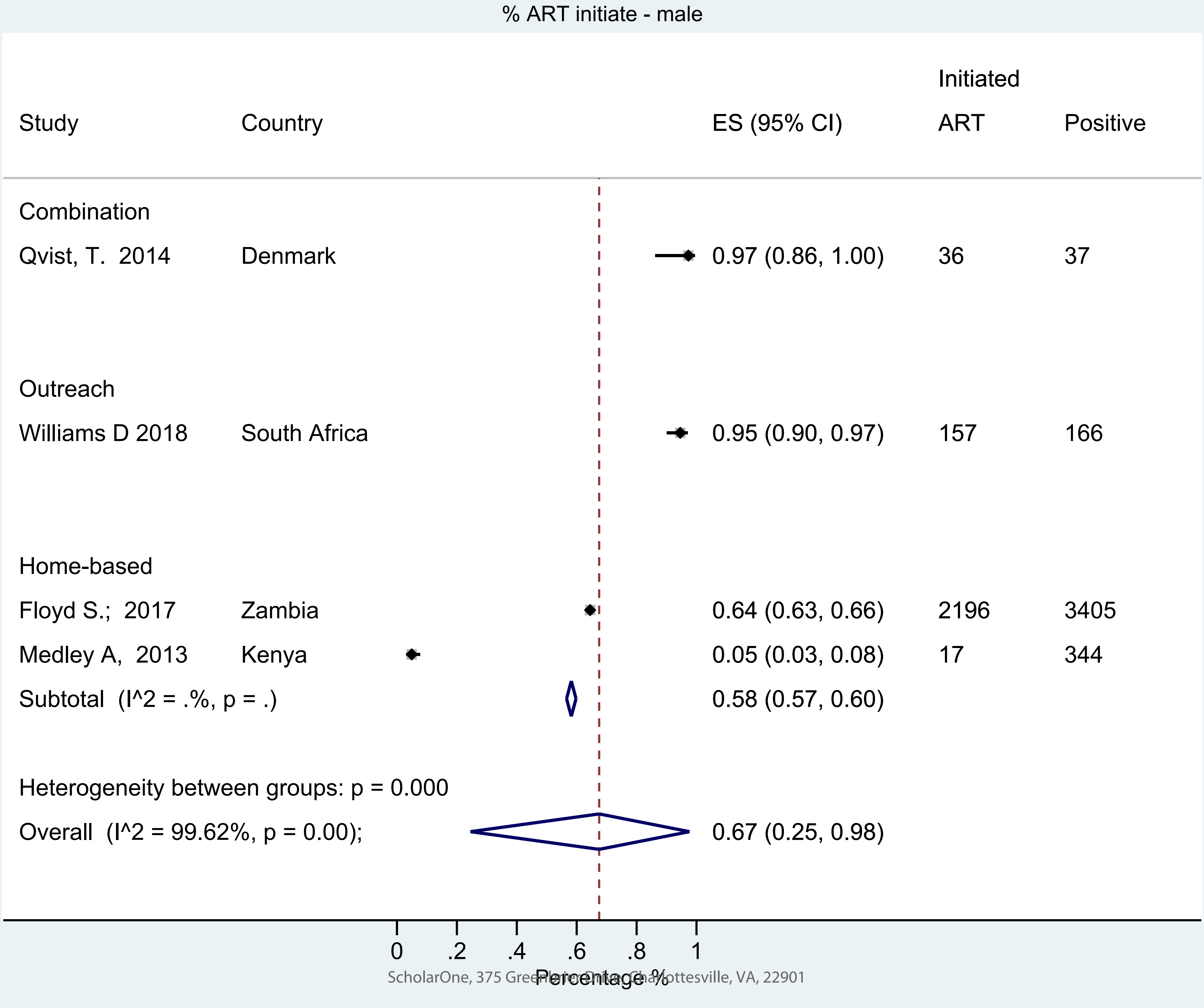
% ART initiate - male
Study
Country
ES (95% CI)
Initiated ART
Positive
Combination
Qvist, T. 2014
Denmark
0.97 (0.86, 1.00)
36
37
Outreach
Williams D 2018
South Africa
0.95 (0.90, 0.97)
157
166
Home-based
Floyd S.; 2017
Zambia
0.64 (0.63, 0.66)
2196
3405
Medley A, 2013
Kenya
0.05 (0.03, 0.08)
17
344
Subtotal (I^2 = .%, p = .)
0.58 (0.57, 0.60)
Heterogeneity between groups: p = 0.000
Overall (I^2 = 99.62%, p = 0.00);
0.67 (0.25, 0.98)
0
.2
.4
.6
.8
1
Percentage %

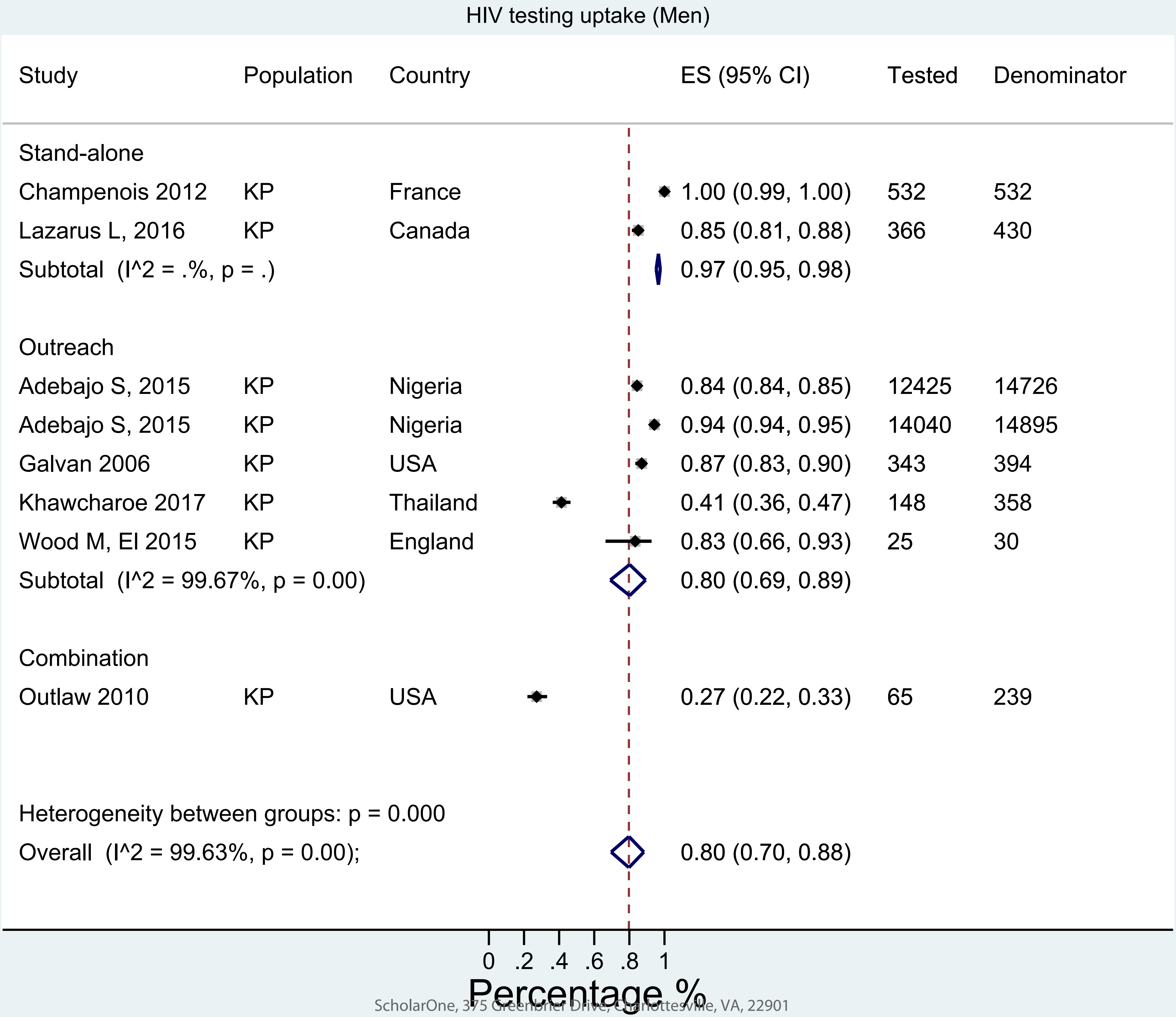
HIV testing uptake (Men)
Study
Population
Country
ES (95% CI)
Tested
Denominator
Stand-alone
Champenois 2012 KP France 1.00 (0.99, 1.00) 532 532
Lazarus L, 2016 KP Canada 0.85 (0.81, 0.88) 366 430
Subtotal (I^2 = .%, p = .) 0.97 (0.95, 0.98)
Outreach
Adebajo S, 2015 KP Nigeria 0.84 (0.84, 0.85) 12425 14726
Adebajo S, 2015 KP Nigeria 0.94 (0.94, 0.95) 14040 14895
Galvan 2006 KP USA 0.87 (0.83, 0.90) 343 394
Khawcharoe 2017 KP Thailand 0.41 (0.36, 0.47) 148 358
Wood M, El 2015 KP England 0.83 (0.66, 0.93) 25 30
Subtotal (I^2 = 99.67%, p = 0.00) 0.80 (0.69, 0.89)
Combination
Outlaw 2010 KP USA 0.27 (0.22, 0.33) 65 239
Heterogeneity between groups: p = 0.000
Overall (I^2 = 99.63%, p = 0.00); 0.80 (0.70, 0.88)
0
.2
.4
.6
.8
1
Percentage %

% tested male

Study	Country	ES (95% CI)	Tested	Tested
**Stand-alone**				
Bailey, A. 2008	England	1.00 (0.99, 1.00)	280	280
Champenois 2012	France	1.00 (0.99, 1.00)	532	532
Des Jarlai 2016	Vietnam	1.00 (0.93, 1.00)	49	49
Ferrer L, 2015	Spain	1.00 (1.00, 1.00)	3544	3544
Kimbrough 2009	USA	0.68 (0.66, 0.69)	2152	3172
Knight, V. 2014	Australia	1.00 (0.98, 1.00)	182	182
Lazarus L, 2016	Canada	0.80 (0.76, 0.83)	366	458
Lessard D. 2016	Canada	1.00 (1.00, 1.00)	1353	1353
Lorente 2013	France	1.00 (0.98, 1.00)	211	211
Meulbroek 2013	Spain	1.00 (1.00, 1.00)	14453	14453
Pham M. 2017	Vietnam	0.90 (0.87, 0.92)	543	603
Robert E B 2016	Ukraine	0.75 (0.72, 0.77)	899	1200
Vannakit R 2016	Thailand	0.71 (0.68, 0.74)	731	1029
Yan, H. et 2014	China	1.00 (1.00, 1.00)	17091	17091
Subtotal (I^2 = 99.80%, p = 0.00)		0.96 (0.91, 1.00)		
**Outreach**				
Adebajo S, 2015	Nigeria	1.00 (1.00, 1.00)	12425	12425
Adebajo S, 2015	Nigeria	1.00 (1.00, 1.00)	14040	14040
Arumainaya 2009	United Kingdom	1.00 (0.98, 1.00)	168	168
Belza MJ, 2015	Spain	1.00 (1.00, 1.00)	3004	3004
Bingham 2008	USA	1.00 (0.99, 1.00)	458	458
Bradshaw 2005	Australia	0.64 (0.58, 0.69)	197	309
Bucher 2007	USA	0.76 (0.73, 0.78)	919	1213
Daskalakis 2009	USA	1.00 (0.99, 1.00)	493	493
DiFranceis 1998	USA	0.56 (0.56, 0.57)	6868	12171
Fernandez- 2014	Spain	1.00 (1.00, 1.00)	2559	2559
Galvan 2006	USA	1.00 (0.99, 1.00)	343	343
Herce ME, 2018	Malawi and Angola	1.00 (1.00, 1.00)	832	832
Ifekandu 2014	Nigeria	1.00 (0.99, 1.00)	356	356
Kakalou 2014	Greece	0.74 (0.66, 0.81)	87	117
Keenan 2001	USA	1.00 (0.74, 1.00)	11	11
Keenan 2001	USA	0.68 (0.65, 0.72)	502	735
Khawcharoe 2017	Thailand	1.00 (0.97, 1.00)	148	148
Krisintu, 2017	Thailand	1.00 (0.98, 1.00)	224	224
Lahuerta 2010	Guatemala	0.44 (0.41, 0.47)	362	823
Liang 2005	USA	0.61 (0.56, 0.65)	268	439
Lipsitz, M 2014	Peru	0.87 (0.85, 0.88)	1387	1602
Lister 2005	Australia	1.00 (0.96, 1.00)	102	102
Marcus U, 2015	Germany	1.00 (1.00, 1.00)	1413	1413
Mdodo 2014	USA	1.00 (1.00, 1.00)	1072	1072
Ngunu-Gitu 2017	Kenya	0.19 (0.18, 0.20)	1255	6602
Rose 2006	USA	0.90 (0.71, 0.97)	19	21
Smyrnov, P 2017	Ukraine	0.69 (0.69, 0.70)	9669	13936
Spielberg, 2005	USA	0.71 (0.66, 0.76)	230	324
Spielberg, 2005	USA	1.00 (0.99, 1.00)	437	437
Wasantioop 2018	Thailand	0.84 (0.82, 0.85)	1606	1923
Wood M, El 2015	England	1.00 (0.87, 1.00)	25	25
Subtotal (I^2 = 99.93%, p = 0.00)		0.93 (0.84, 0.99)		
**Combination**				
Casalini, 2017	Tanzania	0.10 (0.10, 0.11)	3995	39180
Castro R, 2016	Brazil	1.00 (0.99, 1.00)	756	756
Castro R, 2016	Brazil	0.95 (0.94, 0.97)	756	793
Hoenigl M, 2016	United States	1.00 (1.00, 1.00)	8926	8926
Outlaw 2010	USA	1.00 (0.94, 1.00)	65	65
Qvist, T. 2014	Denmark	1.00 (1.00, 1.00)	3012	3012
Ribas Balt 2017	Spain	0.67 (0.66, 0.68)	3602	5385
Stein 2011	USA	1.00 (1.00, 1.00)	1723	1723
Zhang 2014	China	1.00 (1.00, 1.00)	31406	31406
Zulliger R 2017	USA	1.00 (1.00, 1.00)	27475	27475
Subtotal (I^2 = 99.99%, p = 0.00)		0.95 (0.65, 1.00)		
**School-based**				
Gill 2014	United Kingdom	0.40 (0.20, 0.64)	6	15
Miligan 2014	USA	1.00 (0.97, 1.00)	109	109
Subtotal (I^2 = .%, p = .)		0.99 (0.96, 1.00)		
Heterogeneity between groups: p = 0.533				
Overall (I^2 = 99.98%, p = 0.00);		0.94 (0.86, 0.99)		

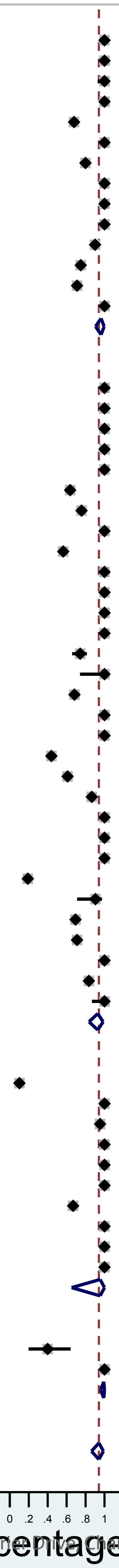

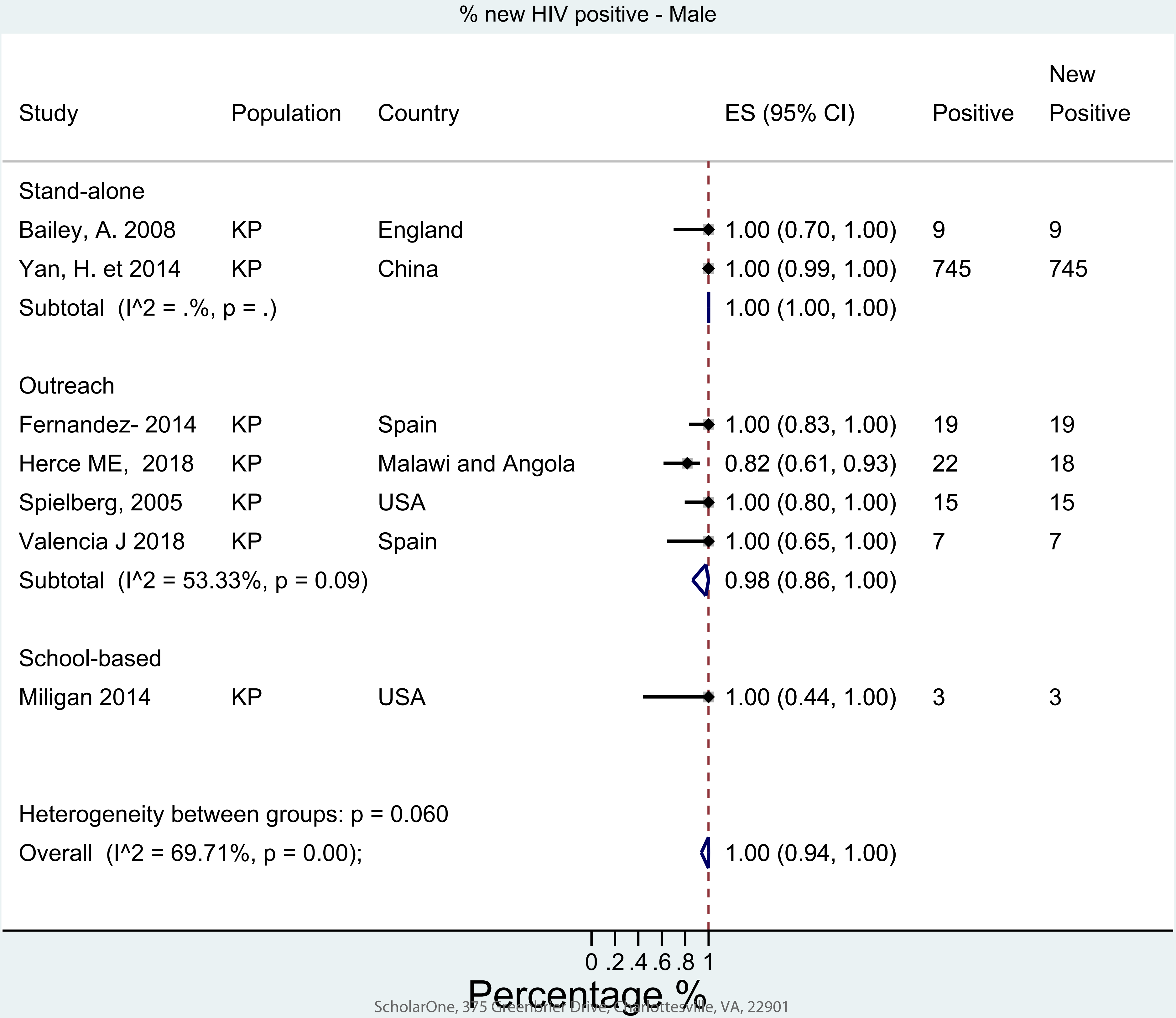
% new HIV positive - Male
Study
Population
Country
ES (95% CI)
Positive
New Positive
Stand-alone
Bailey, A. 2008 KP England 1.00 (0.70, 1.00) 9 9
Yan, H. et 2014 KP China 1.00 (0.99, 1.00) 745 745
Subtotal (I^2 = .%, p = .) 1.00 (1.00, 1.00)
Outreach
Fernandez- 2014 KP Spain 1.00 (0.83, 1.00) 19 19
Herce ME, 2018 KP Malawi and Angola 0.82 (0.61, 0.93) 22 18
Spielberg, 2005 KP USA 1.00 (0.80, 1.00) 15 15
Valencia J 2018 KP Spain 1.00 (0.65, 1.00) 7 7
Subtotal (I^2 = 53.33%, p = 0.09) 0.98 (0.86, 1.00)
School-based
Miligan 2014 KP USA 1.00 (0.44, 1.00) 3 3
Heterogeneity between groups: p = 0.060
Overall (I^2 = 69.71%, p = 0.00); 1.00 (0.94, 1.00)
0 .2 .4 .6 .8 1
Percentage %

% linked - male

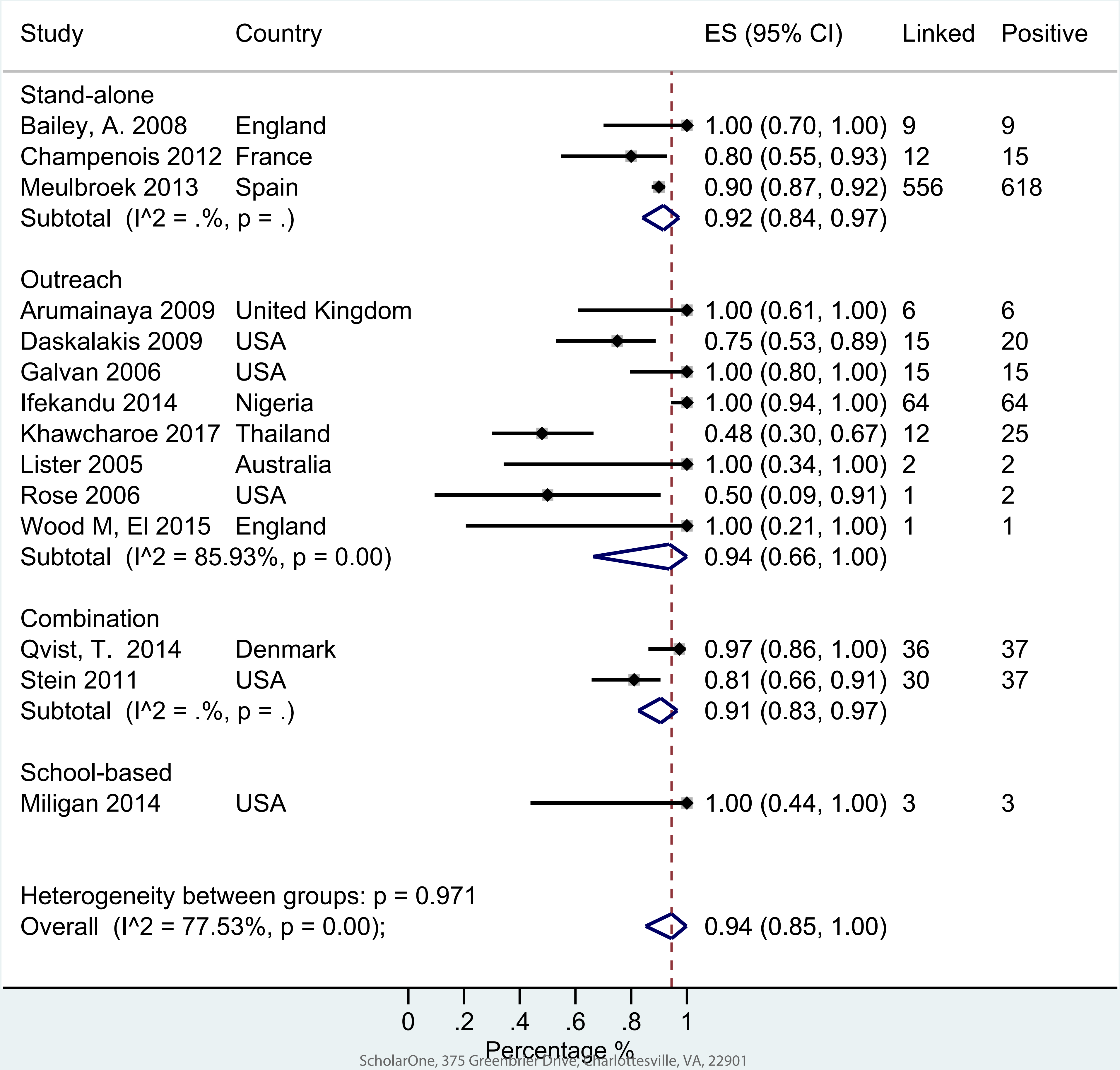
Study
Country
ES (95% CI)
Linked
Positive
Stand-alone
Bailey, A. 2008 England 1.00 (0.70, 1.00) 9 9
Champenois 2012 France 0.80 (0.55, 0.93) 12 15
Meulbroek 2013 Spain 0.90 (0.87, 0.92) 556 618
Subtotal (I^2 = .%, p = .) 0.92 (0.84, 0.97)
Outreach
Arumainaya 2009 United Kingdom 1.00 (0.61, 1.00) 6 6
Daskalakis 2009 USA 0.75 (0.53, 0.89) 15 20
Galvan 2006 USA 1.00 (0.80, 1.00) 15 15
Ifekandu 2014 Nigeria 1.00 (0.94, 1.00) 64 64
Khawcharoe 2017 Thailand 0.48 (0.30, 0.67) 12 25
Lister 2005 Australia 1.00 (0.34, 1.00) 2 2
Rose 2006 USA 0.50 (0.09, 0.91) 1 2
Wood M, El 2015 England 1.00 (0.21, 1.00) 1 1
Subtotal (I^2 = 85.93%, p = 0.00) 0.94 (0.66, 1.00)
Combination
Qvist, T. 2014 Denmark 0.97 (0.86, 1.00) 36 37
Stein 2011 USA 0.81 (0.66, 0.91) 30 37
Subtotal (I^2 = .%, p = .) 0.91 (0.83, 0.97)
School-based
Miligan 2014 USA 1.00 (0.44, 1.00) 3 3
Heterogeneity between groups: p = 0.971
Overall (I^2 = 77.53%, p = 0.00); 0.94 (0.85, 1.00)
0
.2
.4
.6
.8
1
Percentage %

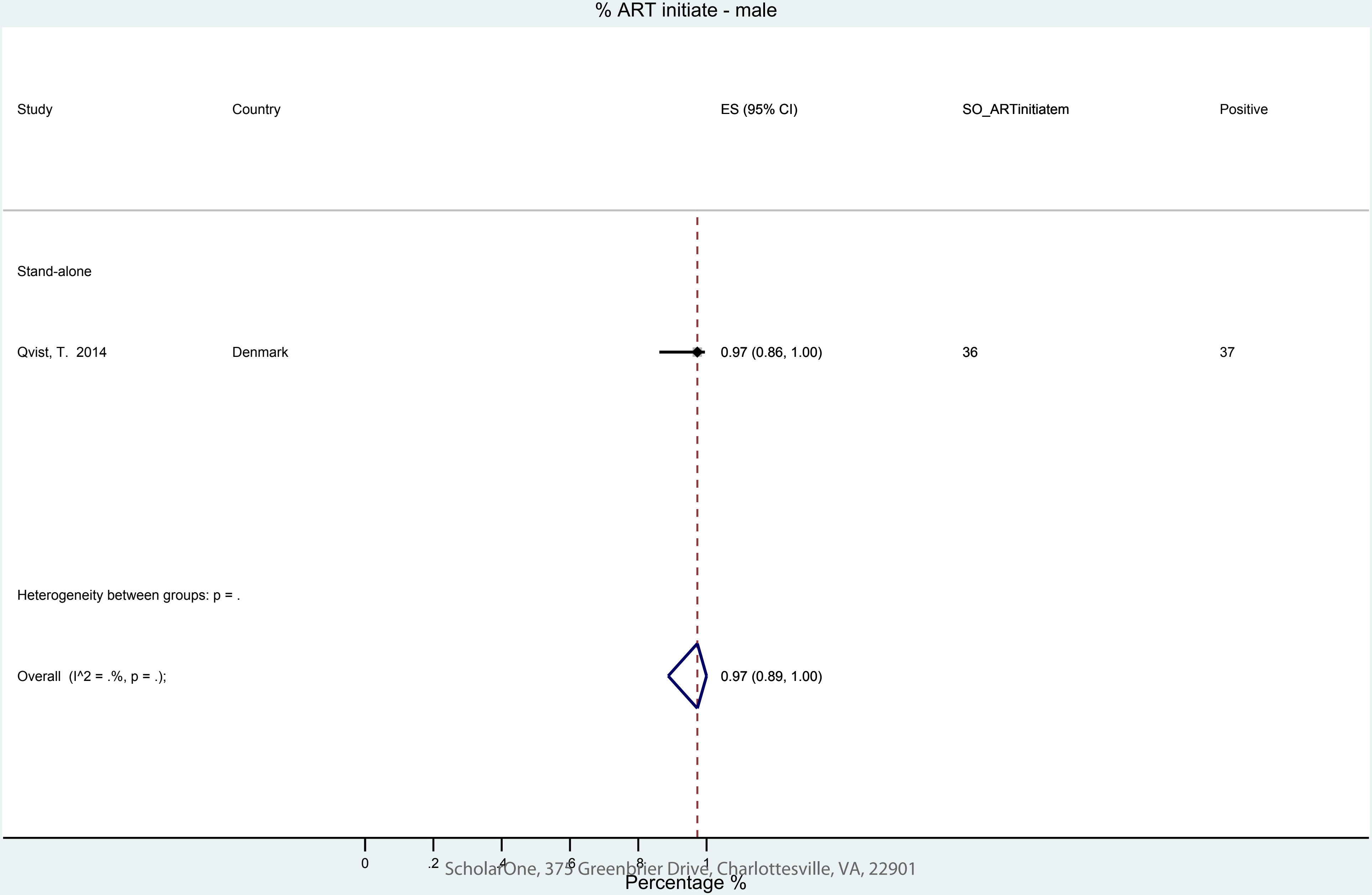

ScholarOne, 375 Greenbrier Drive, Charlottesville, VA, 22901

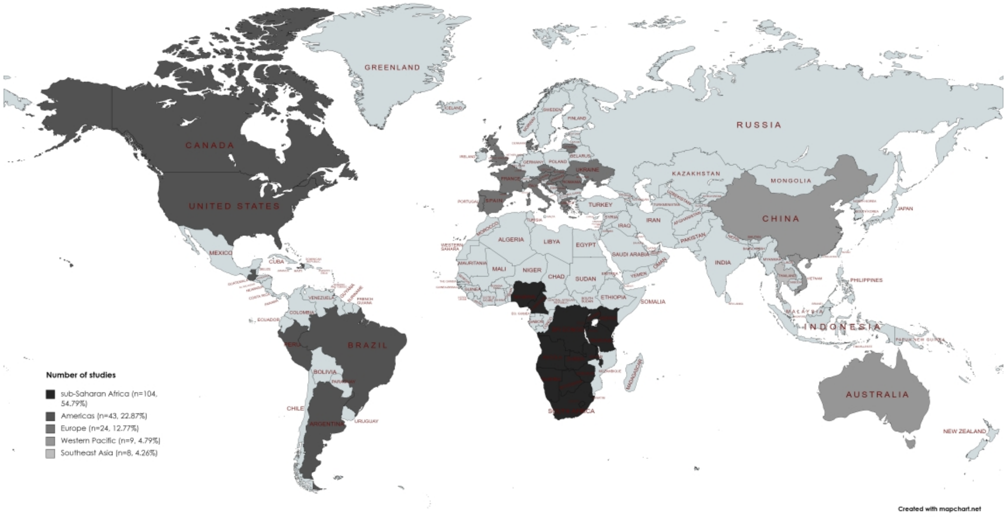

1390x732mm (118 x 118 DPI)

HIV testing uptake (Men)

Study	Population	Country	ES (95% CI)	Tested	Denominator
**Africa**					
Adebajo S, 2015	KP	Nigeria	0.84 (0.84, 0.85)	12425	14726
Adebajo S, 2015	KP	Nigeria	0.94 (0.94, 0.95)	14040	14895
Chamie G.; 2017	GP	Uganda	0.76 (0.75, 0.78)	1929	2530
Ezeanolue 2017	GP	Nigeria	0.84 (0.82, 0.86)	1089	1297
Floyd S.; 2018	GP	Zambia	0.65 (0.64, 0.65)	24177	37265
Fylkesnes 2013	GP	Zambia	0.64 (0.59, 0.69)	204	318
Geoffroy E 2017	GP	Malawi	0.98 (0.97, 0.98)	5693	5820
Krakowiak 2016	GP	Kenya	0.94 (0.91, 0.97)	233	247
Mark J, Ki 2017	GP	Kenya	0.95 (0.87, 0.98)	70	74
Mark J, Ki 2017	GP	Kenya	0.97 (0.93, 0.98)	204	211
Mark J.; K 2015	GP	Kenya	0.84 (0.73, 0.90)	61	73
Phiri M.M. 2016	GP	Zambia and South Africa	0.64 (0.63, 0.64)	17857	28074
Phiri M.M. 2016	GP	Zambia and South Africa	0.68 (0.68, 0.68)	29348	43136
Ruzagira E 2018	GP	Uganda	0.90 (0.90, 0.91)	5273	5837
Shanaube K 2017	GP	Zambia	0.80 (0.79, 0.81)	3526	4400
Shanaube K 2017	GP	Zambia	0.71 (0.71, 0.71)	30226	42596
Tumwesigye 2010	GP	Uganda	0.77 (0.76, 0.77)	123501	161208
Velen K, L 2016	GP	South Africa	0.33 (0.28, 0.38)	109	328
Subtotal (I^2 = 99.89%, p = 0.00)			0.81 (0.76, 0.85)		
**South-East Asia**					
Khawcharoe 2017	KP	Thailand	0.41 (0.36, 0.47)	148	358
**Americas**					
Galvan 2006	KP	USA	0.87 (0.83, 0.90)	343	394
Lazarus L, 2016	KP	Canada	0.85 (0.81, 0.88)	366	430
Oluoch P, 2017	GP	Kenya	1.00 (1.00, 1.00)	35501	35614
Outlaw 2010	KP	USA	0.27 (0.22, 0.33)	65	239
Bungay 2013	KP	Canada	(Excluded)	0	0
Schulden 2008	KP	USA	(Excluded)	0	0
Shrestha 2011	KP	USA	(Excluded)	0	0
Subtotal (I^2 = 99.75%, p = 0.00)			0.80 (0.45, 0.99)		
**Europe**					
Champenois 2012	KP	France	1.00 (0.99, 1.00)	532	532
Wood M, El 2015	KP	England	0.83 (0.66, 0.93)	25	30
Subtotal (I^2 = .%, p = .)			1.00 (1.00, 1.00)		
Heterogeneity between groups: p = 0.000					
Overall (I^2 = 99.95%, p = 0.00);			0.81 (0.75, 0.86)		

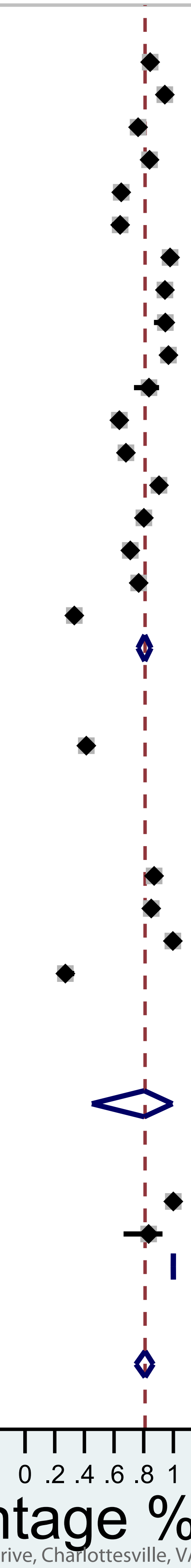

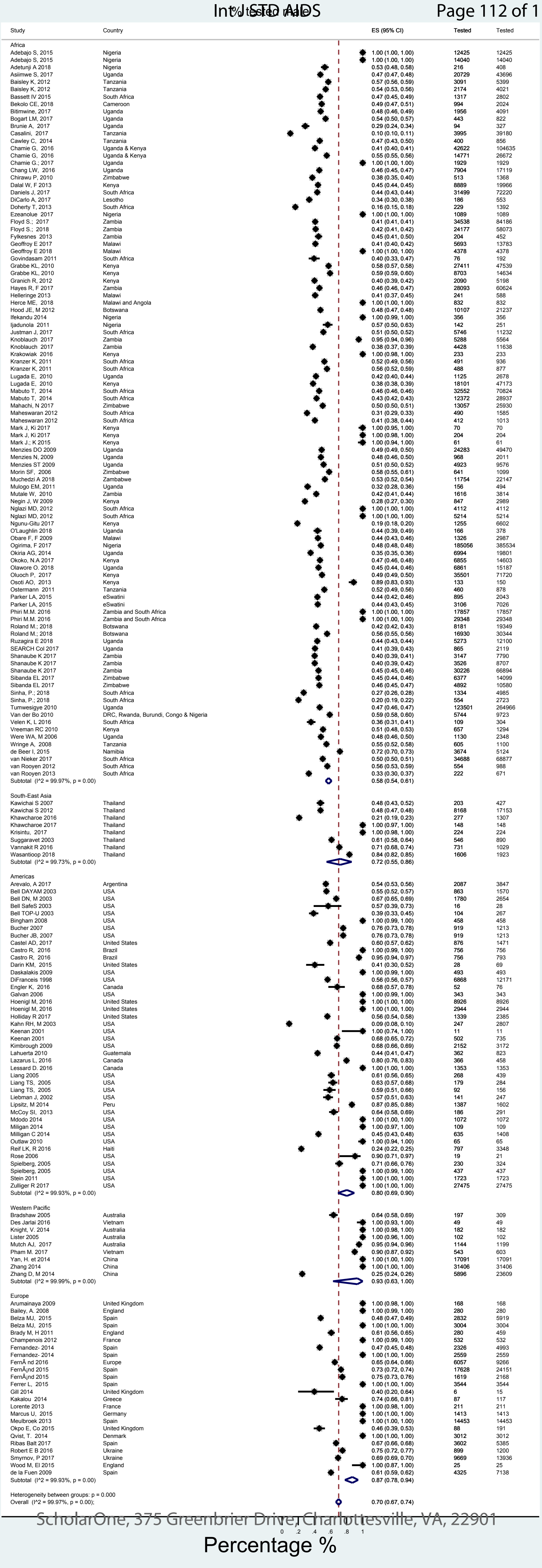
% tested male
Study | Country | ES (95% CI) | Tested | Tested
Africa
Adebajo S, 2015 | Nigeria | 1.00 (1.00, 1.00) | 12425 | 12425
Adebajo S, 2015 | Nigeria | 1.00 (1.00, 1.00) | 14040 | 14040
Adetunji A 2018 | Nigeria | 0.53 (0.48, 0.58) | 216 | 408
Asiimwe S, 2017 | Uganda | 0.47 (0.47, 0.48) | 20729 | 43696
Baisley K, 2012 | Tanzania | 0.57 (0.56, 0.59) | 3091 | 5399
Baisley K, 2012 | Tanzania | 0.54 (0.53, 0.56) | 2174 | 4021
Bassett IV 2015 | South Africa | 0.47 (0.45, 0.49) | 1317 | 2802
Bekolo CE, 2018 | Cameroon | 0.49 (0.47, 0.51) | 994 | 2024
Bitimwine, 2017 | Uganda | 0.48 (0.46, 0.49) | 1956 | 4091
Bogart LM, 2017 | Uganda | 0.54 (0.50, 0.57) | 443 | 822
Brunie A, 2017 | Uganda | 0.29 (0.24, 0.34) | 94 | 327
Casalini, 2017 | Tanzania | 0.10 (0.10, 0.11) | 3995 | 39180
Cawley C, 2014 | Tanzania | 0.47 (0.43, 0.50) | 400 | 856
Chamie G, 2016 | Uganda & Kenya | 0.41 (0.40, 0.41) | 42622 | 104635
Chamie G, 2016 | Uganda & Kenya | 0.55 (0.55, 0.56) | 14771 | 26672
Chamie G.; 2017 | Uganda | 1.00 (1.00, 1.00) | 1929 | 1929
Chang LW, 2016 | Uganda | 0.46 (0.45, 0.47) | 7904 | 17119
Chirawu P, 2010 | Zimbabwe | 0.38 (0.35, 0.40) | 513 | 1368
Dalal W, F 2013 | Kenya | 0.45 (0.44, 0.45) | 8889 | 19966
Daniels J, 2017 | South Africa | 0.44 (0.43, 0.44) | 31499 | 72220
DiCarlo A, 2017 | Lesotho | 0.34 (0.30, 0.38) | 186 | 553
Doherty T, 2013 | South Africa | 0.16 (0.15, 0.18) | 229 | 1392
Ezeanolue 2017 | Nigeria | 1.00 (1.00, 1.00) | 1089 | 1089
Floyd S.; 2017 | Zambia | 0.41 (0.41, 0.41) | 34538 | 84186
Floyd S.; 2018 | Zambia | 0.42 (0.41, 0.42) | 24177 | 58073
Fylkesnes 2013 | Zambia | 0.45 (0.41, 0.50) | 204 | 452
Geoffroy E 2017 | Malawi | 0.41 (0.40, 0.42) | 5693 | 13783
Geoffroy E 2018 | Malawi | 1.00 (1.00, 1.00) | 4378 | 4378
Govindasam 2011 | South Africa | 0.40 (0.33, 0.47) | 76 | 192
Grabbe KL, 2010 | Kenya | 0.58 (0.57, 0.58) | 27411 | 47539
Grabbe KL, 2010 | Kenya | 0.59 (0.59, 0.60) | 8703 | 14634
Granich R, 2012 | Kenya | 0.40 (0.39, 0.42) | 2090 | 5198
Hayes R, F 2017 | Zambia | 0.46 (0.46, 0.47) | 28093 | 60624
Helleringe 2013 | Malawi | 0.41 (0.37, 0.45) | 241 | 588
Herce ME, 2018 | Malawi and Angola | 1.00 (1.00, 1.00) | 832 | 832
Hood JE, M 2012 | Botswana | 0.48 (0.47, 0.48) | 10107 | 21237
Ifekandu 2014 | Nigeria | 1.00 (0.99, 1.00) | 356 | 356
Ijadunola 2011 | Nigeria | 0.57 (0.50, 0.63) | 142 | 251
Justman J, 2017 | South Africa | 0.51 (0.50, 0.52) | 5746 | 11232
Knoblauch 2017 | Zambia | 0.95 (0.94, 0.96) | 5288 | 5564
Knoblauch 2017 | Zambia | 0.38 (0.37, 0.39) | 4428 | 11638
Krakowiak 2016 | Kenya | 1.00 (0.98, 1.00) | 233 | 233
Kranzer K, 2011 | South Africa | 0.52 (0.49, 0.56) | 491 | 936
Kranzer K, 2011 | South Africa | 0.56 (0.52, 0.59) | 488 | 877
Lugada E, 2010 | Uganda | 0.42 (0.40, 0.44) | 1125 | 2678
Lugada E, 2010 | Kenya | 0.38 (0.38, 0.39) | 18101 | 47173
Mabuto T, 2014 | South Africa | 0.46 (0.46, 0.46) | 32552 | 70824
Mabuto T, 2014 | South Africa | 0.43 (0.42, 0.43) | 12372 | 28937
Mahachi, N 2017 | Zimbabwe | 0.50 (0.50, 0.51) | 13057 | 25930
Maheswaran 2012 | South Africa | 0.31 (0.29, 0.33) | 490 | 1585
Maheswaran 2012 | South Africa | 0.41 (0.38, 0.44) | 412 | 1013
Mark J, Ki 2017 | Kenya | 1.00 (0.95, 1.00) | 70 | 70
Mark J, Ki 2017 | Kenya | 1.00 (0.98, 1.00) | 204 | 204
Mark J.; K 2015 | Kenya | 1.00 (0.94, 1.00) | 61 | 61
Menzies DO 2009 | Uganda | 0.49 (0.49, 0.50) | 24283 | 49470
Menzies N, 2009 | Uganda | 0.48 (0.46, 0.50) | 968 | 2011
Menzies ST 2009 | Uganda | 0.51 (0.50, 0.52) | 4923 | 9576
Morin SF, 2006 | Zimbabwe | 0.58 (0.55, 0.61) | 641 | 1099
Muchedzi A 2018 | Zambabwe | 0.53 (0.52, 0.54) | 11754 | 22147
Mulogo EM, 2011 | Uganda | 0.32 (0.28, 0.36) | 156 | 494
Mutale W, 2010 | Zambia | 0.42 (0.41, 0.44) | 1616 | 3814
Negin J, W 2009 | Kenya | 0.28 (0.27, 0.30) | 847 | 2989
Nglazi MD, 2012 | South Africa | 1.00 (1.00, 1.00) | 4112 | 4112
Nglazi MD, 2012 | South Africa | 1.00 (1.00, 1.00) | 5214 | 5214
Ngunu-Gitu 2017 | Kenya | 0.19 (0.18, 0.20) | 1255 | 6602
O'Laughlin 2018 | Uganda | 0.44 (0.39, 0.49) | 166 | 378
Obare F, F 2009 | Malawi | 0.44 (0.43, 0.46) | 1326 | 2987
Ogirima, F 2017 | Nigeria | 0.48 (0.48, 0.48) | 185056 | 385534
Okiria AG, 2014 | Uganda | 0.35 (0.35, 0.36) | 6994 | 19801
Okoko, N.A 2017 | Kenya | 0.47 (0.46, 0.48) | 6855 | 14603
Olawore O. 2018 | Uganda | 0.45 (0.44, 0.46) | 6861 | 15187
Oluoch P, 2017 | Kenya | 0.49 (0.49, 0.50) | 35501 | 71720
Osoti AO, 2013 | Kenya | 0.89 (0.83, 0.93) | 133 | 150
Ostermann 2011 | Tanzania | 0.52 (0.49, 0.56) | 460 | 878
Parker LA, 2015 | eSwatini | 0.44 (0.42, 0.46) | 895 | 2043
Parker LA, 2015 | eSwatini | 0.44 (0.43, 0.45) | 3106 | 7026
Phiri M.M. 2016 | Zambia and South Africa | 1.00 (1.00, 1.00) | 17857 | 17857
Phiri M.M. 2016 | Zambia and South Africa | 1.00 (1.00, 1.00) | 29348 | 29348
Roland M.; 2018 | Botswana | 0.42 (0.42, 0.43) | 8181 | 19349
Roland M.; 2018 | Botswana | 0.56 (0.55, 0.56) | 16930 | 30344
Ruzagira E 2018 | Uganda | 0.44 (0.43, 0.44) | 5273 | 12100
SEARCH Col 2017 | Uganda | 0.41 (0.39, 0.43) | 865 | 2119
Shanaube K 2017 | Zambia | 0.40 (0.39, 0.41) | 3147 | 7790
Shanaube K 2017 | Zambia | 0.40 (0.39, 0.42) | 3526 | 8707
Shanaube K 2017 | Zambia | 0.45 (0.45, 0.46) | 30226 | 66894
Sibanda EL 2017 | Zimbabwe | 0.45 (0.44, 0.46) | 6377 | 14099
Sibanda EL 2017 | Zimbabwe | 0.46 (0.45, 0.47) | 4892 | 10580
Sinha, P.; 2018 | South Africa | 0.27 (0.26, 0.28) | 1334 | 4985
Sinha, P.; 2018 | South Africa | 0.20 (0.19, 0.22) | 554 | 2723
Tumwesigye 2010 | Uganda | 0.47 (0.46, 0.47) | 123501 | 264966
Van der Bo 2010 | DRC, Rwanda, Burundi, Congo & Nigeria | 0.59 (0.58, 0.60) | 5744 | 9723
Velen K, L 2016 | South Africa | 0.36 (0.31, 0.41) | 109 | 304
Vreeman RC 2010 | Kenya | 0.51 (0.48, 0.53) | 657 | 1294
Were WA, M 2006 | Uganda | 0.48 (0.46, 0.50) | 1130 | 2348
Wringe A, 2008 | Tanzania | 0.55 (0.52, 0.58) | 605 | 1100
de Beer I, 2015 | Namibia | 0.72 (0.70, 0.73) | 3674 | 5124
van Nieker 2017 | South Africa | 0.50 (0.50, 0.51) | 34688 | 68877
van Rooyen 2012 | South Africa | 0.56 (0.53, 0.59) | 554 | 988
van Rooyen 2013 | South Africa | 0.33 (0.30, 0.37) | 222 | 671
Subtotal (I^2 = 99.97%, p = 0.00) | 0.58 (0.54, 0.61)
South-East Asia
Kawichai S 2007 | Thailand | 0.48 (0.43, 0.52) | 203 | 427
Kawichai S 2012 | Thailand | 0.48 (0.47, 0.48) | 8168 | 17153
Khawcharoe 2016 | Thailand | 0.21 (0.19, 0.23) | 277 | 1307
Khawcharoe 2017 | Thailand | 1.00 (0.97, 1.00) | 148 | 148
Krisintu, 2017 | Thailand | 1.00 (0.98, 1.00) | 224 | 224
Suggaravet 2003 | Thailand | 0.61 (0.58, 0.64) | 546 | 890
Vannakit R 2016 | Thailand | 0.71 (0.68, 0.74) | 731 | 1029
Wasantioop 2018 | Thailand | 0.84 (0.82, 0.85) | 1606 | 1923
Subtotal (I^2 = 99.73%, p = 0.00) | 0.72 (0.55, 0.86)
Americas
Arevalo, A 2017 | Argentina | 0.54 (0.53, 0.56) | 2087 | 3847
Bell DAYAM 2003 | USA | 0.55 (0.52, 0.57) | 863 | 1570
Bell DN, M 2003 | USA | 0.67 (0.65, 0.69) | 1780 | 2654
Bell SafeS 2003 | USA | 0.57 (0.39, 0.73) | 16 | 28
Bell TOP-U 2003 | USA | 0.39 (0.33, 0.45) | 104 | 267
Bingham 2008 | USA | 1.00 (0.99, 1.00) | 458 | 458
Bucher 2007 | USA | 0.76 (0.73, 0.78) | 919 | 1213
Bucher JB, 2007 | USA | 0.76 (0.73, 0.78) | 919 | 1213
Castel AD, 2017 | United States | 0.60 (0.57, 0.62) | 876 | 1471
Castro R, 2016 | Brazil | 1.00 (0.99, 1.00) | 756 | 756
Castro R, 2016 | Brazil | 0.95 (0.94, 0.97) | 756 | 793
Darin KM, 2015 | United States | 0.41 (0.30, 0.52) | 28 | 69
Daskalakis 2009 | USA | 1.00 (0.99, 1.00) | 493 | 493
DiFranceis 1998 | USA | 0.56 (0.56, 0.57) | 6868 | 12171
Engler K, 2016 | Canada | 0.68 (0.57, 0.78) | 52 | 76
Galvan 2006 | USA | 1.00 (0.99, 1.00) | 343 | 343
Hoenigl M, 2016 | United States | 1.00 (1.00, 1.00) | 8926 | 8926
Hoenigl M, 2016 | United States | 1.00 (1.00, 1.00) | 2944 | 2944
Holliday R 2017 | United States | 0.56 (0.54, 0.58) | 1339 | 2385
Kahn RH, M 2003 | USA | 0.09 (0.08, 0.10) | 247 | 2807
Keenan 2001 | USA | 1.00 (0.74, 1.00) | 11 | 11
Keenan 2001 | USA | 0.68 (0.65, 0.72) | 502 | 735
Kimbrough 2009 | USA | 0.68 (0.66, 0.69) | 2152 | 3172
Lahuerta 2010 | Guatemala | 0.44 (0.41, 0.47) | 362 | 823
Lazarus L, 2016 | Canada | 0.80 (0.76, 0.83) | 366 | 458
Lessard D. 2016 | Canada | 1.00 (1.00, 1.00) | 1353 | 1353
Liang 2005 | USA | 0.61 (0.56, 0.65) | 268 | 439
Liang TS, 2005 | USA | 0.63 (0.57, 0.68) | 179 | 284
Liang TS, 2005 | USA | 0.59 (0.51, 0.66) | 92 | 156
Liebman J, 2002 | USA | 0.57 (0.51, 0.63) | 141 | 247
Lipsitz, M 2014 | Peru | 0.87 (0.85, 0.88) | 1387 | 1602
McCoy SI, 2013 | USA | 0.64 (0.58, 0.69) | 186 | 291
Mdodo 2014 | USA | 1.00 (1.00, 1.00) | 1072 | 1072
Miligan 2014 | USA | 1.00 (0.97, 1.00) | 109 | 109
Milligan C 2014 | USA | 0.45 (0.43, 0.48) | 635 | 1408
Outlaw 2010 | USA | 1.00 (0.94, 1.00) | 65 | 65
Reif LK, R 2016 | Haiti | 0.24 (0.22, 0.25) | 797 | 3348
Rose 2006 | USA | 0.90 (0.71, 0.97) | 19 | 21
Spielberg, 2005 | USA | 0.71 (0.66, 0.76) | 230 | 324
Spielberg, 2005 | USA | 1.00 (0.99, 1.00) | 437 | 437
Stein 2011 | USA | 1.00 (1.00, 1.00) | 1723 | 1723
Zulliger R 2017 | USA | 1.00 (1.00, 1.00) | 27475 | 27475
Subtotal (I^2 = 99.93%, p = 0.00) | 0.80 (0.69, 0.90)
Western Pacific
Bradshaw 2005 | Australia | 0.64 (0.58, 0.69) | 197 | 309
Des Jarlai 2016 | Vietnam | 1.00 (0.93, 1.00) | 49 | 49
Knight, V. 2014 | Australia | 1.00 (0.98, 1.00) | 182 | 182
Lister 2005 | Australia | 1.00 (0.96, 1.00) | 102 | 102
Mutch AJ, 2017 | Australia | 0.95 (0.94, 0.96) | 1144 | 1199
Pham M. 2017 | Vietnam | 0.90 (0.87, 0.92) | 543 | 603
Yan, H. et 2014 | China | 1.00 (1.00, 1.00) | 17091 | 17091
Zhang 2014 | China | 1.00 (1.00, 1.00) | 31406 | 31406
Zhang D, M 2014 | China | 0.25 (0.24, 0.26) | 5896 | 23609
Subtotal (I^2 = 99.99%, p = 0.00) | 0.93 (0.63, 1.00)
Europe
Arumainaya 2009 | United Kingdom | 1.00 (0.98, 1.00) | 168 | 168
Bailey, A. 2008 | England | 1.00 (0.99, 1.00) | 280 | 280
Belza MJ, 2015 | Spain | 0.48 (0.47, 0.49) | 2832 | 5919
Belza MJ, 2015 | Spain | 1.00 (1.00, 1.00) | 3004 | 3004
Brady M, H 2011 | England | 0.61 (0.56, 0.65) | 280 | 459
Champenois 2012 | France | 1.00 (0.99, 1.00) | 532 | 532
Fernandez- 2014 | Spain | 0.47 (0.45, 0.48) | 2326 | 4993
Fernandez- 2014 | Spain | 1.00 (1.00, 1.00) | 2559 | 2559
FernÃ nd 2016 | Europe | 0.65 (0.64, 0.66) | 6057 | 9266
FernÃ¡nd 2015 | Spain | 0.73 (0.72, 0.74) | 17628 | 24151
FernÃ¡nd 2015 | Spain | 0.75 (0.73, 0.76) | 1619 | 2168
Ferrer L, 2015 | Spain | 1.00 (1.00, 1.00) | 3544 | 3544
Gill 2014 | United Kingdom | 0.40 (0.20, 0.64) | 6 | 15
Kakalou 2014 | Greece | 0.74 (0.66, 0.81) | 87 | 117
Lorente 2013 | France | 1.00 (0.98, 1.00) | 211 | 211
Marcus U, 2015 | Germany | 1.00 (1.00, 1.00) | 1413 | 1413
Meulbroek 2013 | Spain | 1.00 (1.00, 1.00) | 14453 | 14453
Okpo E, Co 2015 | United Kingdom | 0.46 (0.39, 0.53) | 88 | 191
Qvist, T. 2014 | Denmark | 1.00 (1.00, 1.00) | 3012 | 3012
Ribas Balt 2017 | Spain | 0.67 (0.66, 0.68) | 3602 | 5385
Robert E B 2016 | Ukraine | 0.75 (0.72, 0.77) | 899 | 1200
Smyrnov, P 2017 | Ukraine | 0.69 (0.69, 0.70) | 9669 | 13936
Wood M, El 2015 | England | 1.00 (0.87, 1.00) | 25 | 25
de la Fuen 2009 | Spain | 0.61 (0.59, 0.62) | 4325 | 7138
Subtotal (I^2 = 99.93%, p = 0.00) | 0.87 (0.78, 0.94)
Heterogeneity between groups: p = 0.000
Overall (I^2 = 99.97%, p = 0.00); | 0.70 (0.67, 0.74)
0 .2 .4 .6 .8 1
Percentage %

% new HIV positive - Male

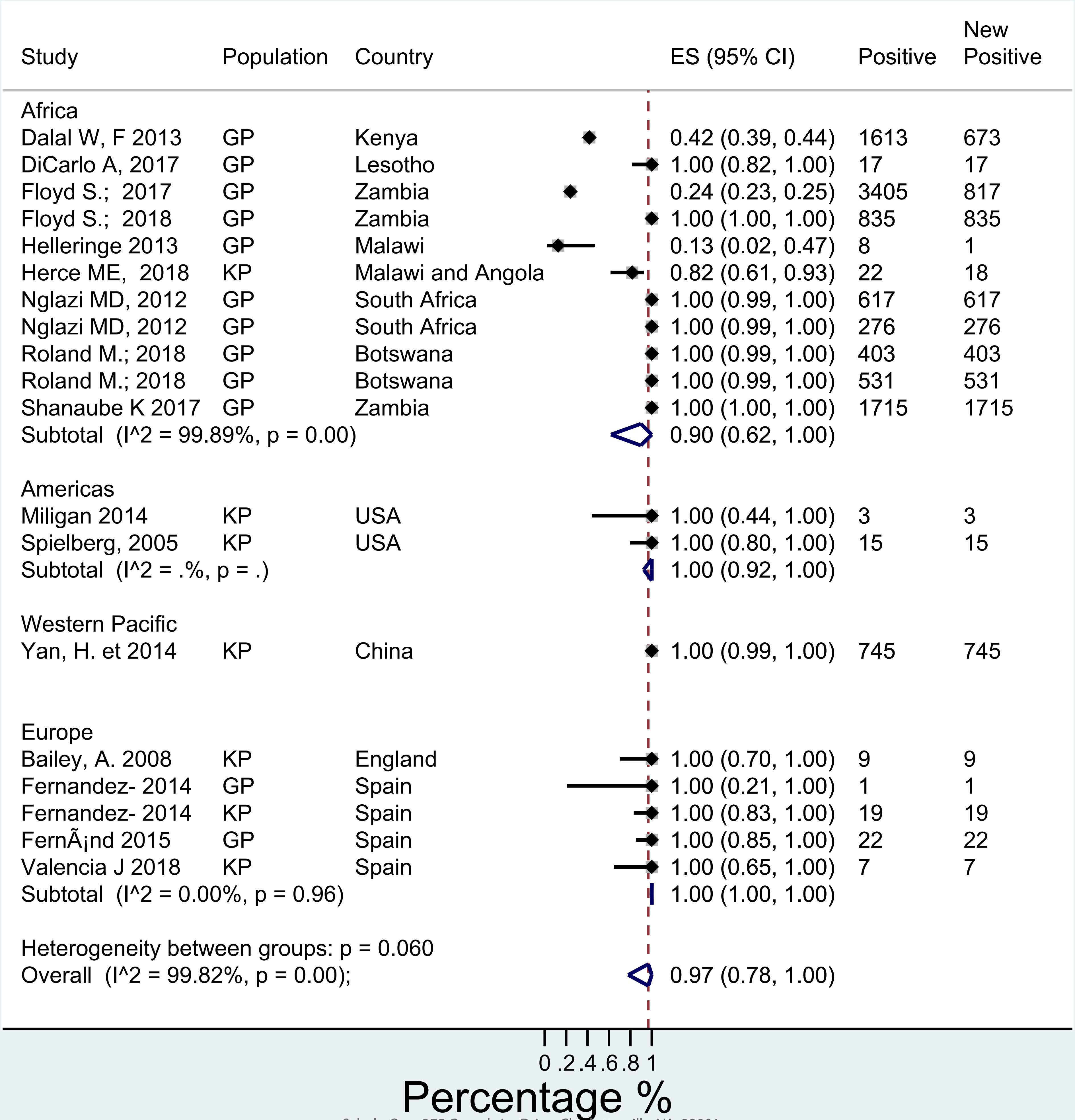

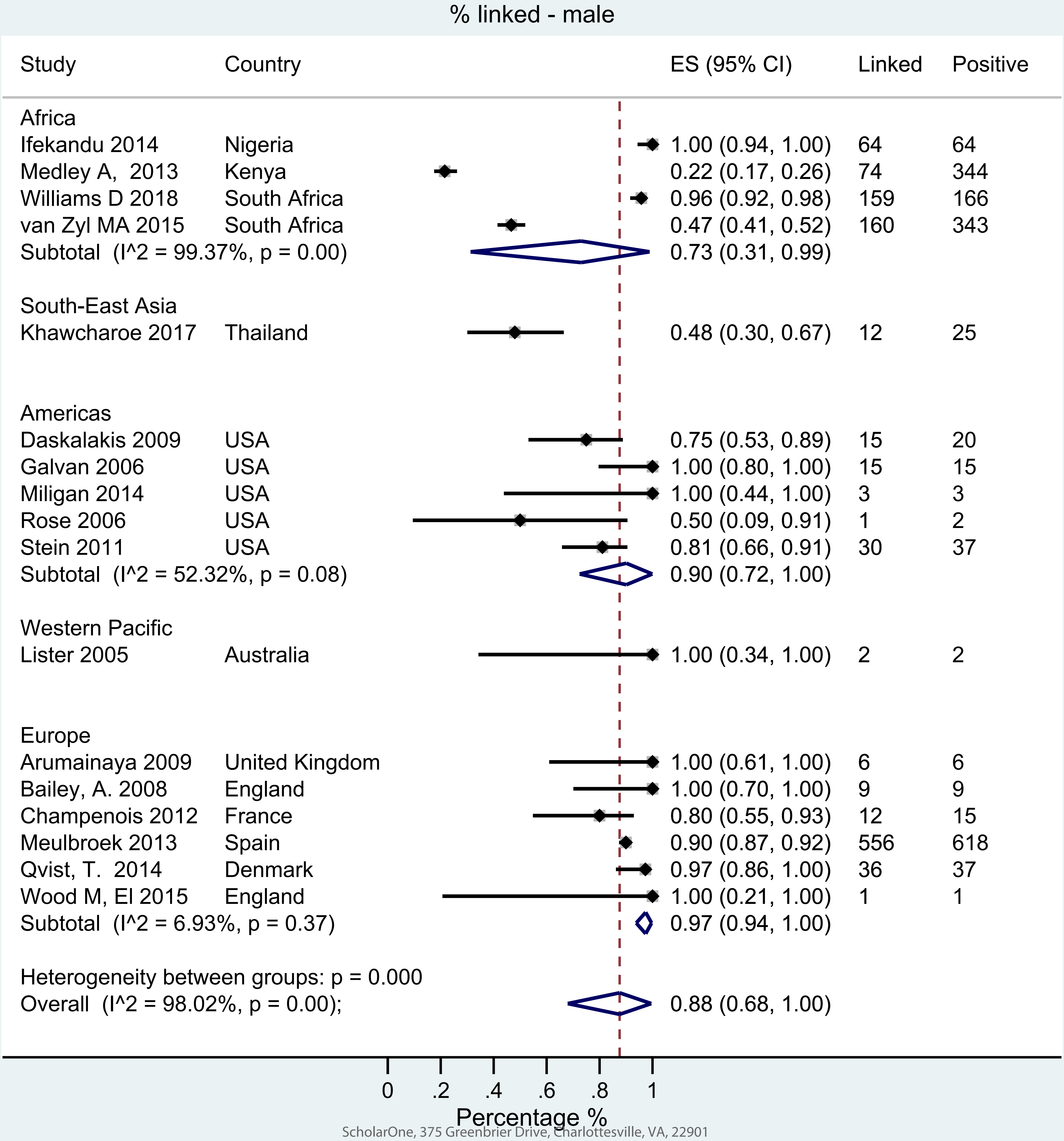
% linked - male
Study
Country
ES (95% CI)
Linked
Positive
Africa
Ifekandu 2014 Nigeria 1.00 (0.94, 1.00) 64 64
Medley A, 2013 Kenya 0.22 (0.17, 0.26) 74 344
Williams D 2018 South Africa 0.96 (0.92, 0.98) 159 166
van Zyl MA 2015 South Africa 0.47 (0.41, 0.52) 160 343
Subtotal (I^2 = 99.37%, p = 0.00) 0.73 (0.31, 0.99)
South-East Asia
Khawcharoe 2017 Thailand 0.48 (0.30, 0.67) 12 25
Americas
Daskalakis 2009 USA 0.75 (0.53, 0.89) 15 20
Galvan 2006 USA 1.00 (0.80, 1.00) 15 15
Miligan 2014 USA 1.00 (0.44, 1.00) 3 3
Rose 2006 USA 0.50 (0.09, 0.91) 1 2
Stein 2011 USA 0.81 (0.66, 0.91) 30 37
Subtotal (I^2 = 52.32%, p = 0.08) 0.90 (0.72, 1.00)
Western Pacific
Lister 2005 Australia 1.00 (0.34, 1.00) 2 2
Europe
Arumainaya 2009 United Kingdom 1.00 (0.61, 1.00) 6 6
Bailey, A. 2008 England 1.00 (0.70, 1.00) 9 9
Champenois 2012 France 0.80 (0.55, 0.93) 12 15
Meulbroek 2013 Spain 0.90 (0.87, 0.92) 556 618
Qvist, T. 2014 Denmark 0.97 (0.86, 1.00) 36 37
Wood M, El 2015 England 1.00 (0.21, 1.00) 1 1
Subtotal (I^2 = 6.93%, p = 0.37) 0.97 (0.94, 1.00)
Heterogeneity between groups: p = 0.000
Overall (I^2 = 98.02%, p = 0.00); 0.88 (0.68, 1.00)
0
.2
.4
.6
.8
1
Percentage %

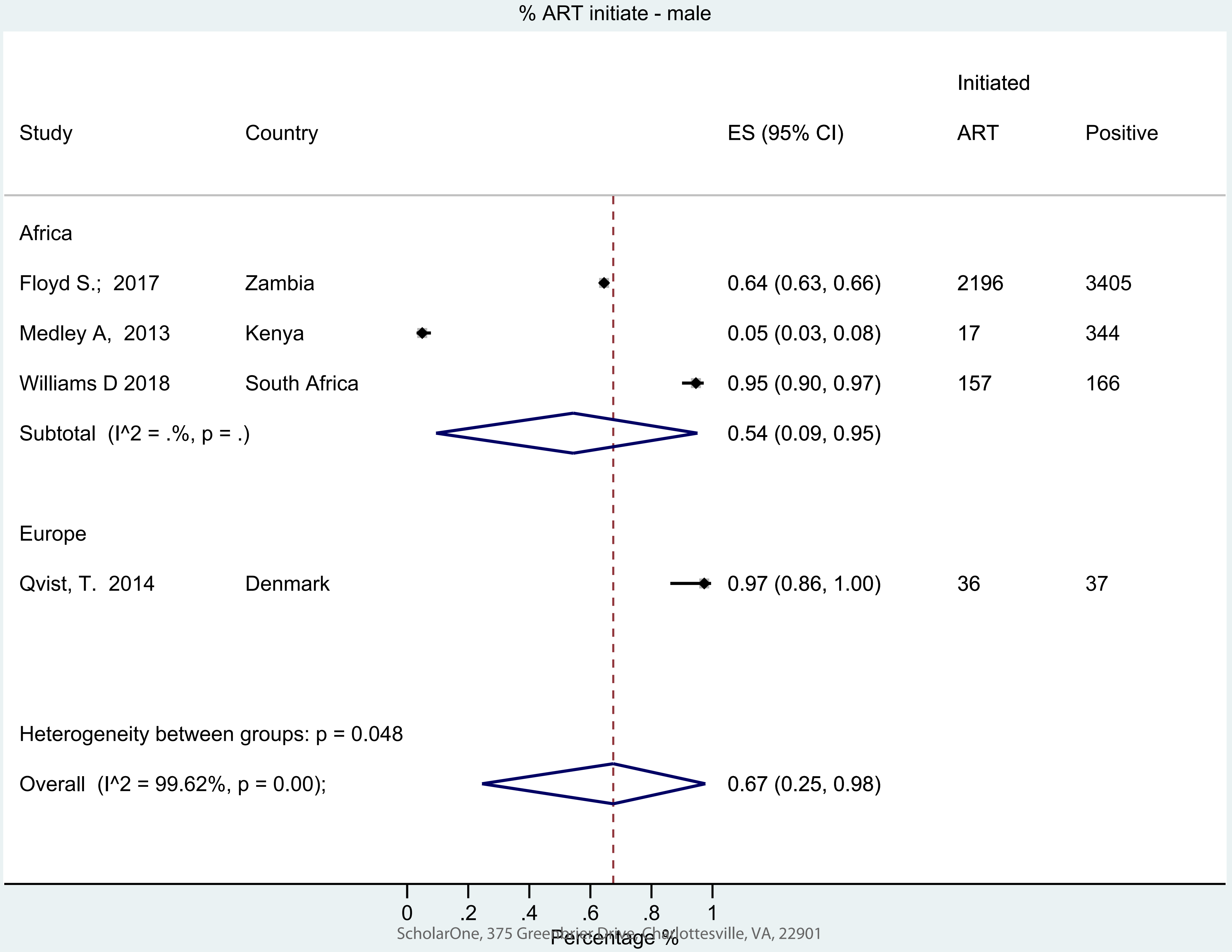
% ART initiate - male
Study
Country
ES (95% CI)
Initiated ART
Positive
Africa
Floyd S.; 2017
Zambia
0.64 (0.63, 0.66)
2196
3405
Medley A, 2013
Kenya
0.05 (0.03, 0.08)
17
344
Williams D 2018
South Africa
0.95 (0.90, 0.97)
157
166
Subtotal (I^2 = .%, p = .)
0.54 (0.09, 0.95)
Europe
Qvist, T. 2014
Denmark
0.97 (0.86, 1.00)
36
37
Heterogeneity between groups: p = 0.048
Overall (I^2 = 99.62%, p = 0.00);
0.67 (0.25, 0.98)
0
.2
.4
.6
.8
1
Percentage %

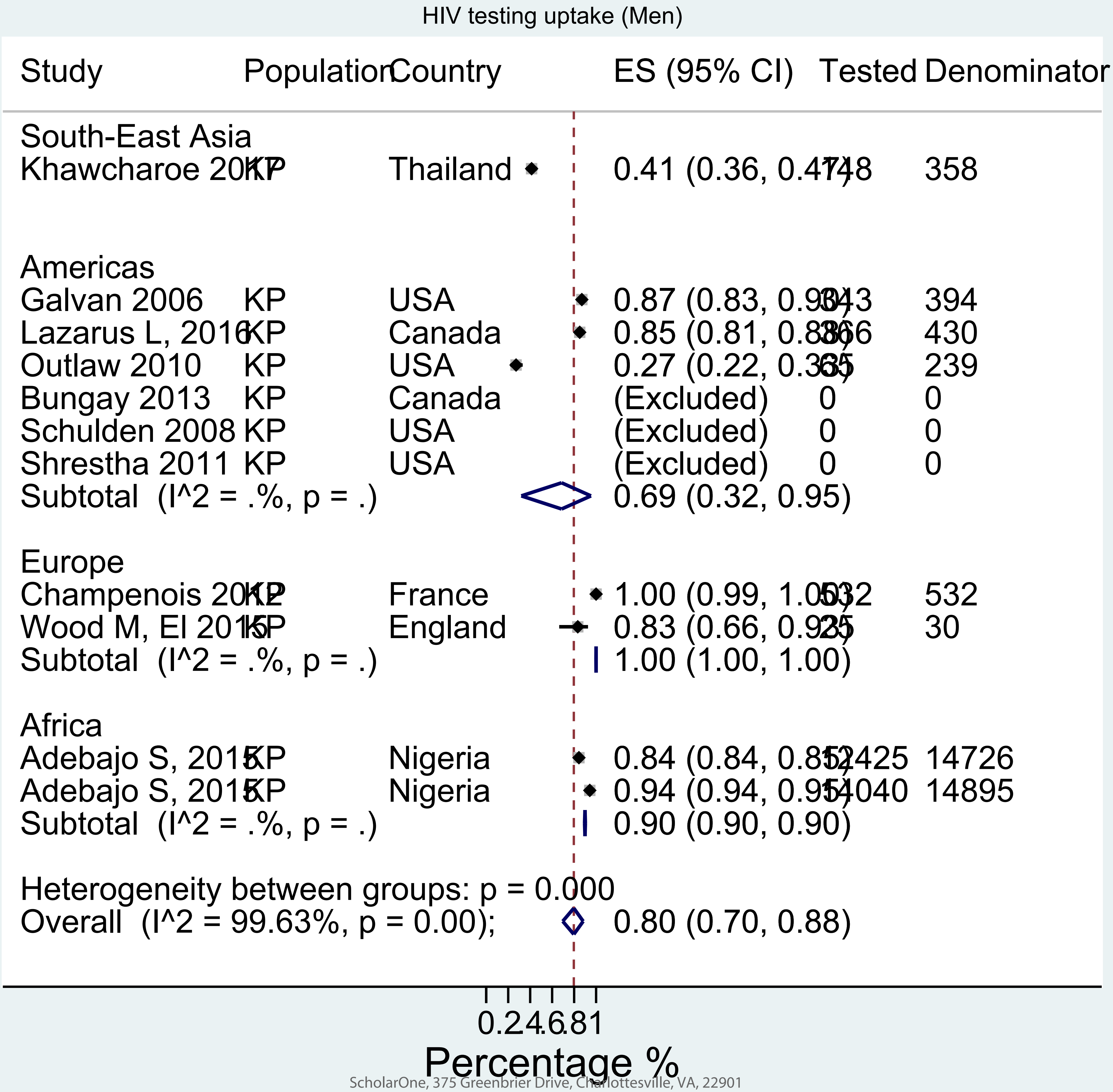
HIV testing uptake (Men)
Study
Population
Country
ES (95% CI)
Tested
Denominator
South-East Asia
Thailand
358
Americas
Galvan 2006
KP
USA
394
KP
Canada
430
Outlaw 2010
KP
USA
239
Bungay 2013
KP
Canada
(Excluded)
0
0
Schulden 2008
KP
USA
(Excluded)
0
0
Shrestha 2011
KP
USA
(Excluded)
0
0
Subtotal (I^2 = .%, p = .)
0.69 (0.32, 0.95)
Europe
France
532
England
30
Subtotal (I^2 = .%, p = .)
1.00 (1.00, 1.00)
Africa
Nigeria
14726
Nigeria
14895
Subtotal (I^2 = .%, p = .)
0.90 (0.90, 0.90)
Heterogeneity between groups: p = 0.000
Overall (I^2 = 99.63%, p = 0.00);
0.80 (0.70, 0.88)
Percentage %

% tested male

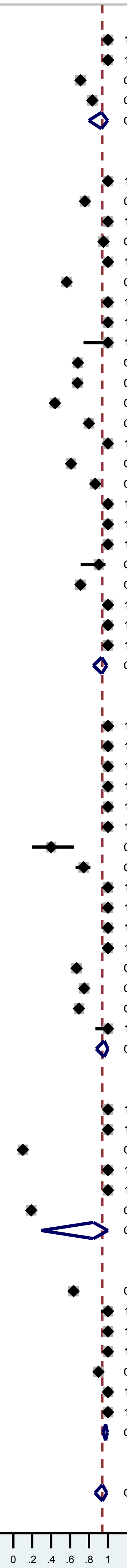

Study	Country	ES (95% CI)	Tested	Tested
South-East Asia				
Khawcharoe 2017	Thailand	1.00 (0.97, 1.00)	148	148
Krisintu, 2017	Thailand	1.00 (0.98, 1.00)	224	224
Vannakit R 2016	Thailand	0.71 (0.68, 0.74)	731	1029
Wasantioop 2018	Thailand	0.84 (0.82, 0.85)	1606	1923
Subtotal (I^2 = 99.03%, p = 0.00)		0.93 (0.80, 1.00)		
Americas				
Bingham 2008	USA	1.00 (0.99, 1.00)	458	458
Bucher 2007	USA	0.76 (0.73, 0.78)	919	1213
Castro R, 2016	Brazil	1.00 (0.99, 1.00)	756	756
Castro R, 2016	Brazil	0.95 (0.94, 0.97)	756	793
Daskalakis 2009	USA	1.00 (0.99, 1.00)	493	493
DiFranceis 1998	USA	0.56 (0.56, 0.57)	6868	12171
Galvan 2006	USA	1.00 (0.99, 1.00)	343	343
Hoenigl M, 2016	United States	1.00 (1.00, 1.00)	8926	8926
Keenan 2001	USA	1.00 (0.74, 1.00)	11	11
Keenan 2001	USA	0.68 (0.65, 0.72)	502	735
Kimbrough 2009	USA	0.68 (0.66, 0.69)	2152	3172
Lahuerta 2010	Guatemala	0.44 (0.41, 0.47)	362	823
Lazarus L, 2016	Canada	0.80 (0.76, 0.83)	366	458
Lessard D. 2016	Canada	1.00 (1.00, 1.00)	1353	1353
Liang 2005	USA	0.61 (0.56, 0.65)	268	439
Lipsitz, M 2014	Peru	0.87 (0.85, 0.88)	1387	1602
Mdodo 2014	USA	1.00 (1.00, 1.00)	1072	1072
Miligan 2014	USA	1.00 (0.97, 1.00)	109	109
Outlaw 2010	USA	1.00 (0.94, 1.00)	65	65
Rose 2006	USA	0.90 (0.71, 0.97)	19	21
Spielberg, 2005	USA	0.71 (0.66, 0.76)	230	324
Spielberg, 2005	USA	1.00 (0.99, 1.00)	437	437
Stein 2011	USA	1.00 (1.00, 1.00)	1723	1723
Zulliger R 2017	USA	1.00 (1.00, 1.00)	27475	27475
Subtotal (I^2 = 99.91%, p = 0.00)		0.93 (0.85, 0.99)		
Europe				
Arumainaya 2009	United Kingdom	1.00 (0.98, 1.00)	168	168
Bailey, A. 2008	England	1.00 (0.99, 1.00)	280	280
Belza MJ, 2015	Spain	1.00 (1.00, 1.00)	3004	3004
Champenois 2012	France	1.00 (0.99, 1.00)	532	532
Fernandez- 2014	Spain	1.00 (1.00, 1.00)	2559	2559
Ferrer L, 2015	Spain	1.00 (1.00, 1.00)	3544	3544
Gill 2014	United Kingdom	0.40 (0.20, 0.64)	6	15
Kakalou 2014	Greece	0.74 (0.66, 0.81)	87	117
Lorente 2013	France	1.00 (0.98, 1.00)	211	211
Marcus U, 2015	Germany	1.00 (1.00, 1.00)	1413	1413
Meulbroek 2013	Spain	1.00 (1.00, 1.00)	14453	14453
Qvist, T. 2014	Denmark	1.00 (1.00, 1.00)	3012	3012
Ribas Balt 2017	Spain	0.67 (0.66, 0.68)	3602	5385
Robert E B 2016	Ukraine	0.75 (0.72, 0.77)	899	1200
Smyrnov, P 2017	Ukraine	0.69 (0.69, 0.70)	9669	13936
Wood M, El 2015	England	1.00 (0.87, 1.00)	25	25
Subtotal (I^2 = 99.91%, p = 0.00)		0.96 (0.88, 1.00)		
Africa				
Adebajo S, 2015	Nigeria	1.00 (1.00, 1.00)	12425	12425
Adebajo S, 2015	Nigeria	1.00 (1.00, 1.00)	14040	14040
Casalini, 2017	Tanzania	0.10 (0.10, 0.11)	3995	39180
Herce ME, 2018	Malawi and Angola	1.00 (1.00, 1.00)	832	832
Ifekandu 2014	Nigeria	1.00 (0.99, 1.00)	356	356
Ngunu-Gitu 2017	Kenya	0.19 (0.18, 0.20)	1255	6602
Subtotal (I^2 = 100.00%, p = 0.00)		0.85 (0.30, 1.00)		
Western Pacific				
Bradshaw 2005	Australia	0.64 (0.58, 0.69)	197	309
Des Jarlai 2016	Vietnam	1.00 (0.93, 1.00)	49	49
Knight, V. 2014	Australia	1.00 (0.98, 1.00)	182	182
Lister 2005	Australia	1.00 (0.96, 1.00)	102	102
Pham M. 2017	Vietnam	0.90 (0.87, 0.92)	543	603
Yan, H. et 2014	China	1.00 (1.00, 1.00)	17091	17091
Zhang 2014	China	1.00 (1.00, 1.00)	31406	31406
Subtotal (I^2 = 99.20%, p = 0.00)		0.98 (0.95, 0.99)		
Heterogeneity between groups: p = 0.629				
Overall (I^2 = 99.98%, p = 0.00);		0.94 (0.86, 0.99)		

% new HIV positive - Male

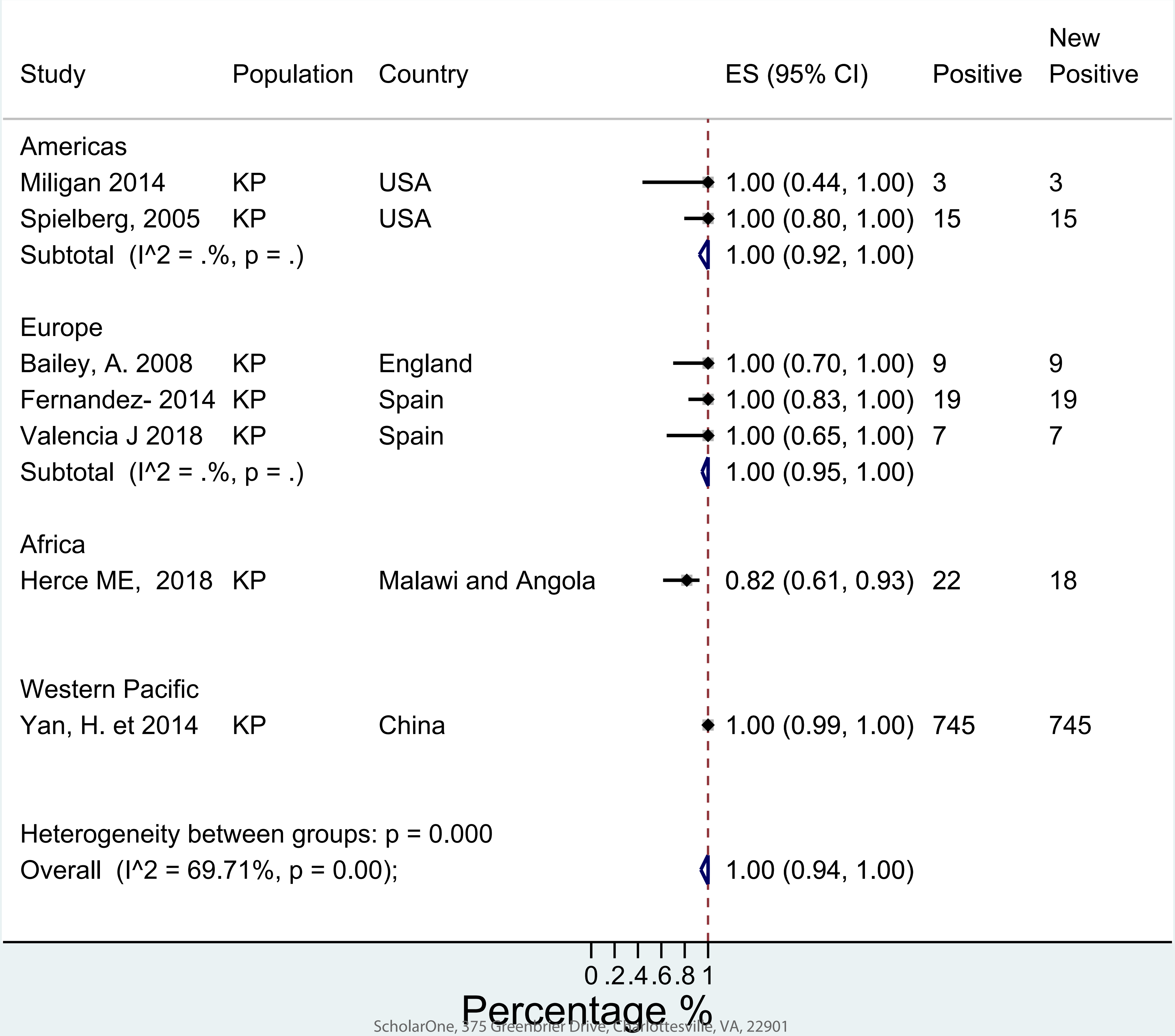

Supplement: Supplemental Material - A systematic review and meta-analysis of the evidence for community-based HIV testing on men’s engagement in the HIV care cascade [file sj-pdf-1-std-10.1177_09564624221111277.pdf]
